# Supplementary material for: Generalizable anchor aptamer strategy for loading nucleic acid therapeutics on exosomes
Source: EMBO Mol Med. 2024 Mar 6;16(4):24. doi: 10.1038/s44321-024-00049-7 (PMC11018858; doi:10.1038/s44321-024-00049-7)
Supplement: Supplementary file 1 — Appendix [file 44321_2024_49_MOESM1_ESM.pdf]

Generalizable anchor aptamer strategy for loading nucleic acid therapeutics on exosomes

Gang Han[1], Yao Zhang[1], Li Zhong[1], Biaobiao Wang[1], Shuai Qiu[1], Jun Song[1], Caorui Lin[1], Fangdi Zou[2], Jingqiao Wu[1], Huanan Yu[1], Chao Liang[3], Ke Wen[4], Yiqi Seow[5], HaiFang Yin[1,6]*

APPENDIX

Contents: Appendix Figures S1 and S2, Appendix Table S1

Appendix Figure S1- Page 2

Representative gating strategy for flow cytometric analysis of DiR- labeled exosomes and DiR- and FITC-positive exosomes.

Appendix Figure S2 - Page 2

Flow cytometric analysis of DNA aptamer enrichment for round 3, 9 and 10 during SELEX.

Appendix Table S1 - Page 3-4

Nomenclature and sequences for the DNA aptamer library, primers and tested aptamers.

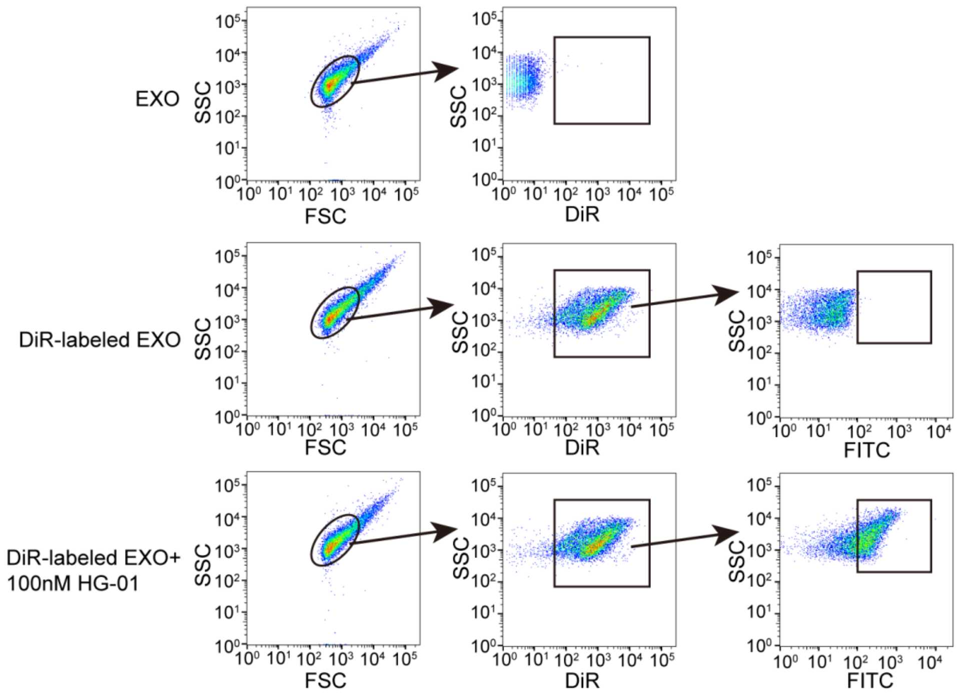

Appendix Figure S1. Representative gating strategy for flow cytometric analysis of DiR-labeled exosomes and DiR- and FITC-positive exosomes. Exosomes were derived from differentiated C2C12 cells and labelled with DiR. HG-01 DNA aptamer was labelled with FITC.

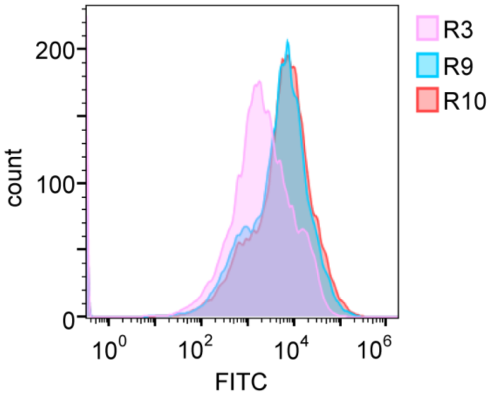

Appendix Figure S2. Flow cytometric analysis of DNA aptamer enrichment for round 3, 9 and 10 during SELEX.

Appendix Table S1

Nomenclature	Sequence (from 5' to 3' end)
sDNA aptamer library	ATCCAGAGTGACGCAGCA-40N-TGGACACGGTGGCTTAGT
Forward Primer for ssDNA Pool	FITC-ATCCAGAGTGACGCAGCA
Reverse Primer for ssDNA Pool	biotin-ACTAAGCCACCGTGTCCA
HG-01	FITC-ATCCAGAGTGACGCAGCAGGGGGTTTGGTTTGGCGGGGTGGCTCCCCGGGGTTGGTTATGGACACGGTGGCTTAGT
HG-02	FITC-ATCCAGAGTGACGCAGCAAATGGTTGGGCCGGATAGG TTGGTTG GATACGGTTTTGCTTGGACACGGTGGCTTAGT
HG-03	ATCCAGAGTGACGCAGCAGGATTTGTGGGTGGGTGGGCGGACCGCGAGTCGAACGTTCTGGACACGGTGGCTTAGT
HG-04	ATCCAGAGTGACGCAGCATAGGTTTGGGGTTGGAGGGAGGTCGT TCGCGGATCTGGATTGGACACGGTGGCTTAGT
HG-05	ATCCAGAGTGACGCAGCAGAAGTGCGGGGGACTGCTCGGGATT GCGGATCGTTCTCGCTGGACACGGTGGCTTAGT
HG-06:	ATCCAGAGTGACGCAGCATGTGGGCGGGTGGGTGGTCTTGCGGC ATCTCGGAGGCAGGTGGACACGGTGGCTTAGT
HG-07:	ATCCAGAGTGACGCAGCACTAGGTTGGAGTAGGTTTGGTGTGCTGGTTATTTCAGTCGTGGACACGGTGGCTTAGT

HG-08:	ATCCAGAGTGACGCAGCACCGGGGATGTAGGGGCAGGGGTTCG AGTGAGGAGTATTATGGACACGGTGGCTTAGT
HG-09	FITC-ATCCAGAGTGACGCAGCAGGGTGGAACGAGAAGGCGGATCGCG CGGCCTGGACCAATGTGGACACGGTGGCTTAGT
EAA	FITC/Cy5-GGGGGTTTGGTTTGGCGGGGTGGCTCCCCGGGGT TGGTTA
CD63 aptamer	FITC/Cy5-CACCCCACCTCGCTCCCGTGACACTAATGCTA
EAA-NU172	FITC-*GGGGTTTGGTTTGGCGGGGTGGCTCCCCGGGGTTGGTTA***TGGAC**CGCCTAGGTTGGGTAGGGTGGTGGCG
NU172	FITC-CGCCTAGGTTGGGTAGGGTGGTGGCG
EAA-linker	FITC-*GGGGTTTGGTTTGGCGGGGTGGCTCCCCGGGGTTGGTTA***TGGACACGGTG**
linker-FIXa	FITC-**rCrArCrCrGrUrGrUrCrCrAr**ArUrGrGrGrGrArCrUrArUrArCrCrGrCrGrUrArArUrGrCrUrGrCrCrUrCrCrCrCrArU
EAA-PMOC	FITC-*GGGGGTTTGGTTTGGCGGGGTGGCTCCCCGGGGTTGGTTA***ATTTCAGGTAAGCCGAGGTTTGGCC**
PMO	FITC-GGCCAAACCTCGGCTTACCTGAAAT

Appendix Table S1. Nomenclature and sequences for the DNA aptamer library, primers and tested aptamers. Underlined letters refer to the 18-base linker. Italic letters refer to EAA sequences and underlined bold letters represent linker sequences for different aptamers. r means RNA. Bold letters represent the sequences complementary to PMO. FITC, Cy5 and Biotin refer to the dyes used.
