# Supplementary figures and images for: Generalizable anchor aptamer strategy for loading nucleic acid therapeutics on exosomes
Source: EMBO Mol Med. 2024 Mar 6;16(4):24. doi: 10.1038/s44321-024-00049-7 (PMC11018858; doi:10.1038/s44321-024-00049-7)

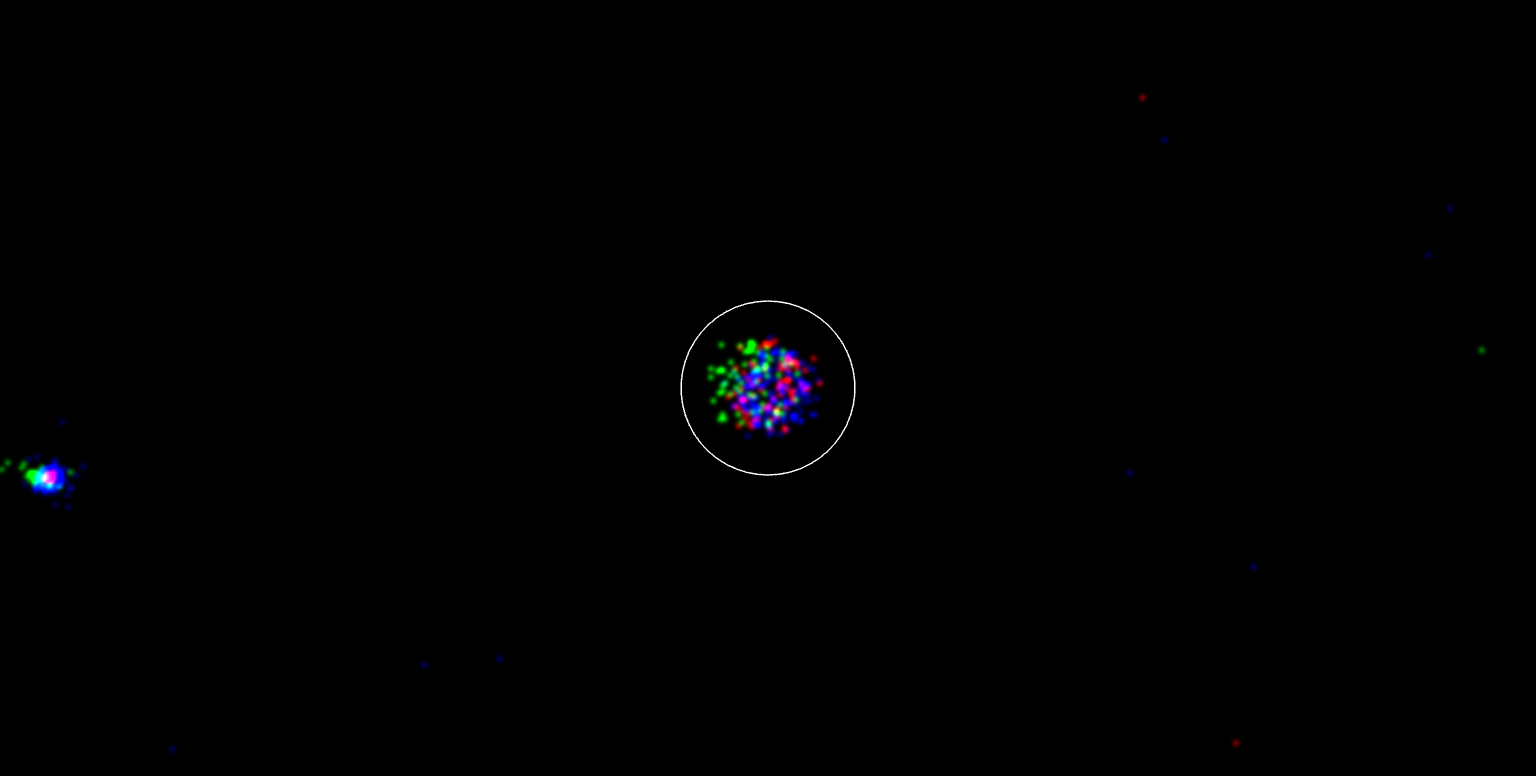

Supplement: Supplementary file 3 — Source Data Fig. 2 [file 44321_2024_49_MOESM3_ESM.zip › Figure 2/2C/CD63&81&9.tif]

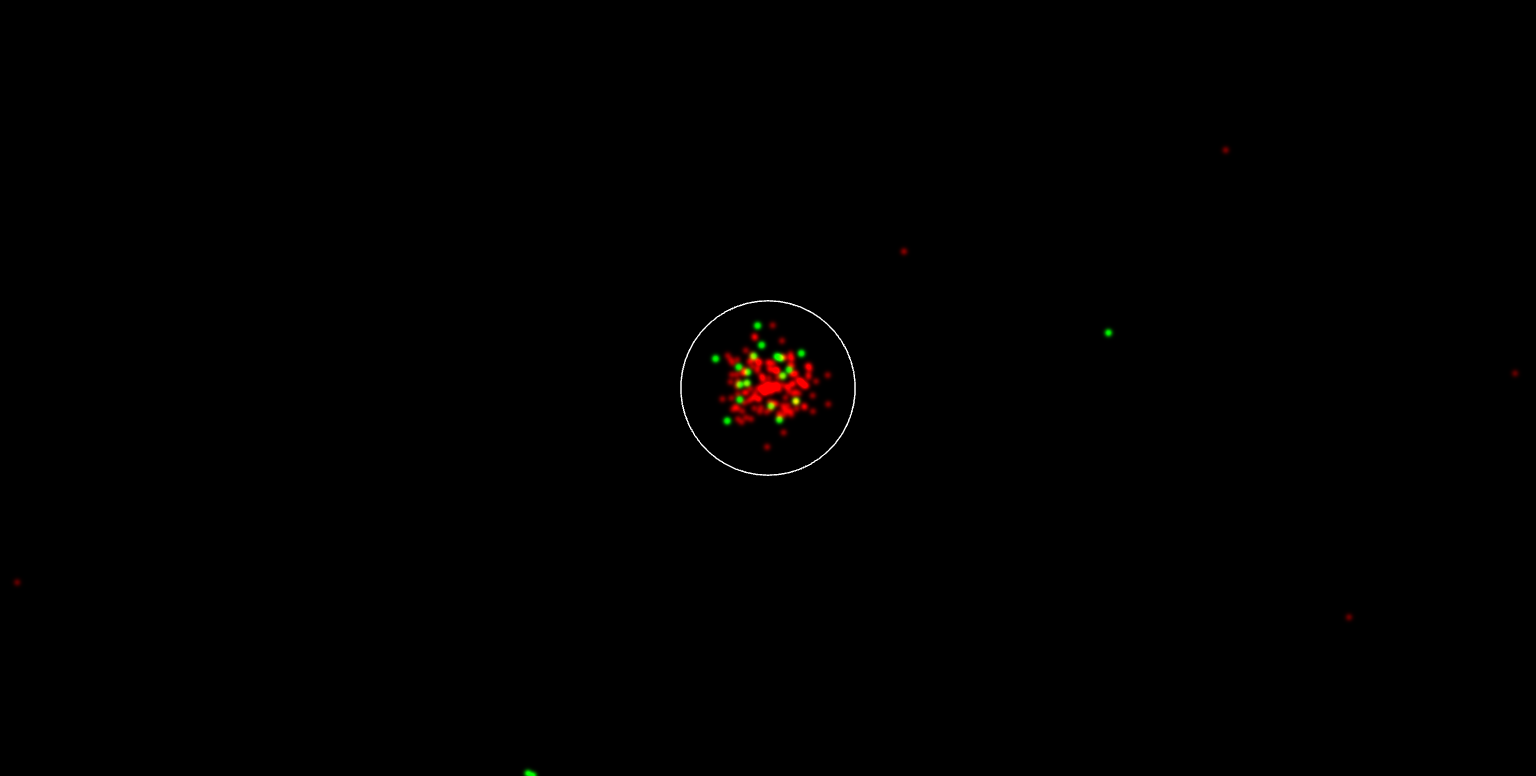

Supplement: Supplementary file 3 — Source Data Fig. 2 [file 44321_2024_49_MOESM3_ESM.zip › Figure 2/2C/EAA.tif]

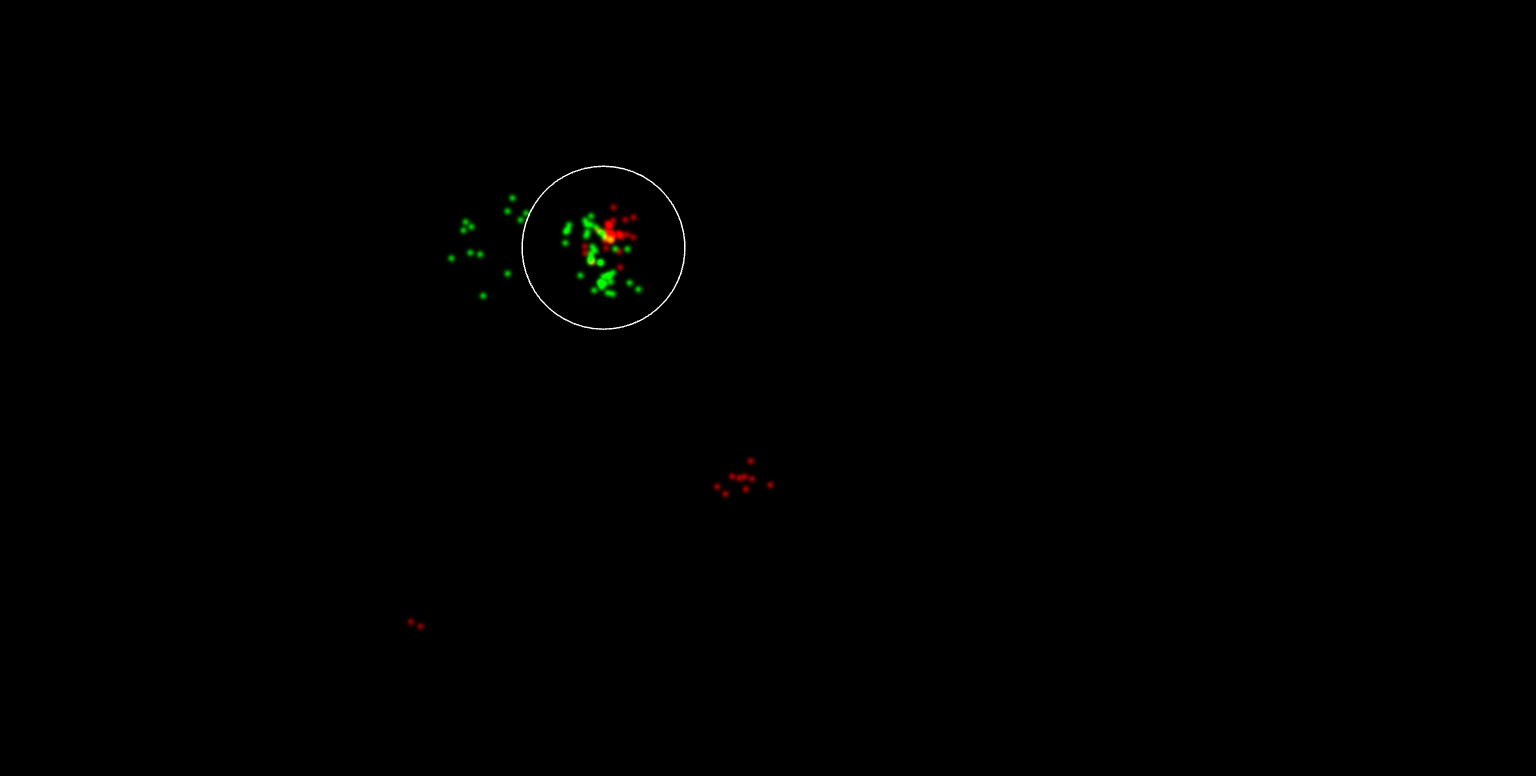

Supplement: Supplementary file 3 — Source Data Fig. 2 [file 44321_2024_49_MOESM3_ESM.zip › Figure 2/2C/CD63 apt.tif]

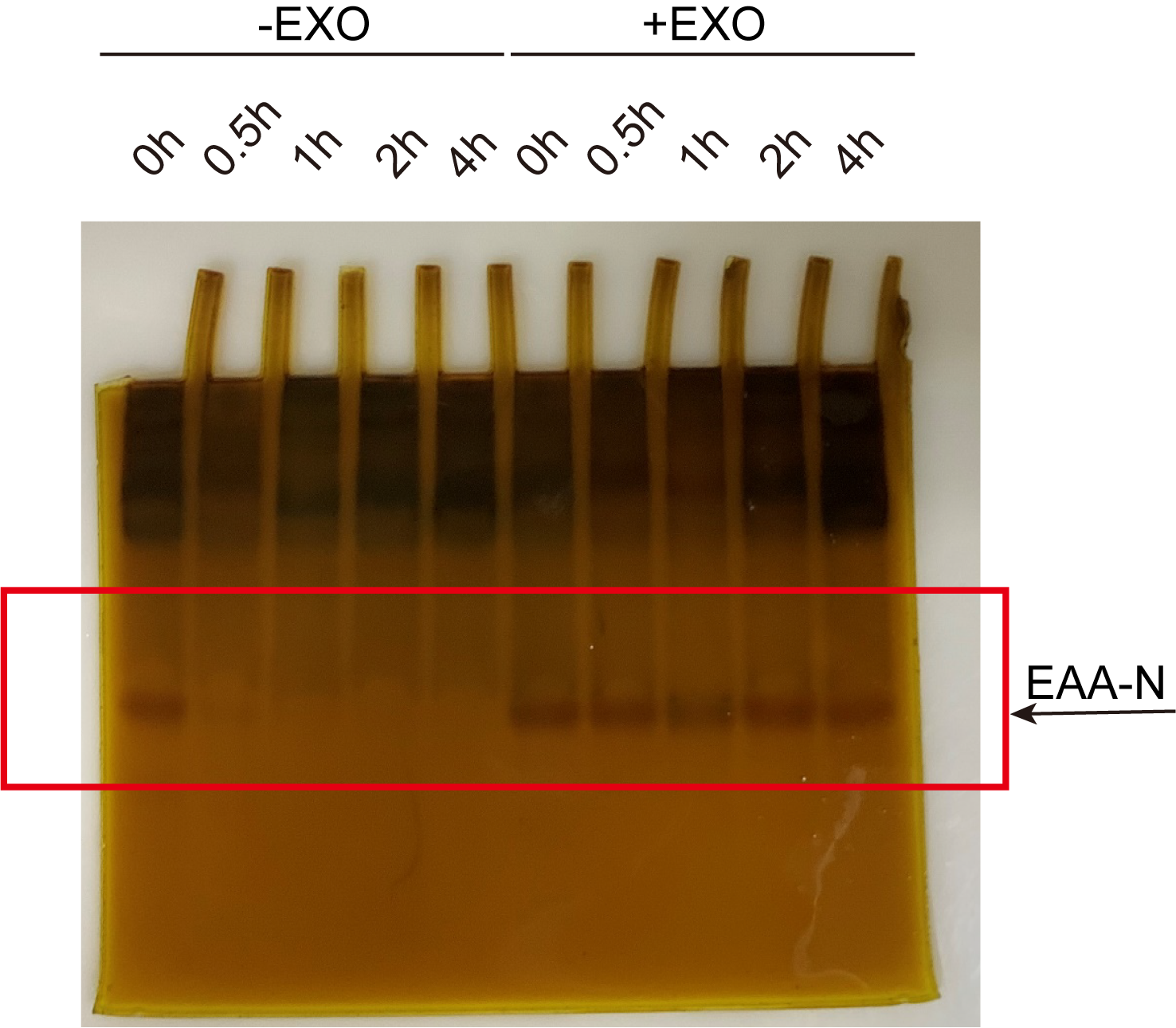

Supplement: Supplementary file 4 — Source Data Fig. 3 [file 44321_2024_49_MOESM4_ESM.zip › Figure 3/3C/3C.tif]

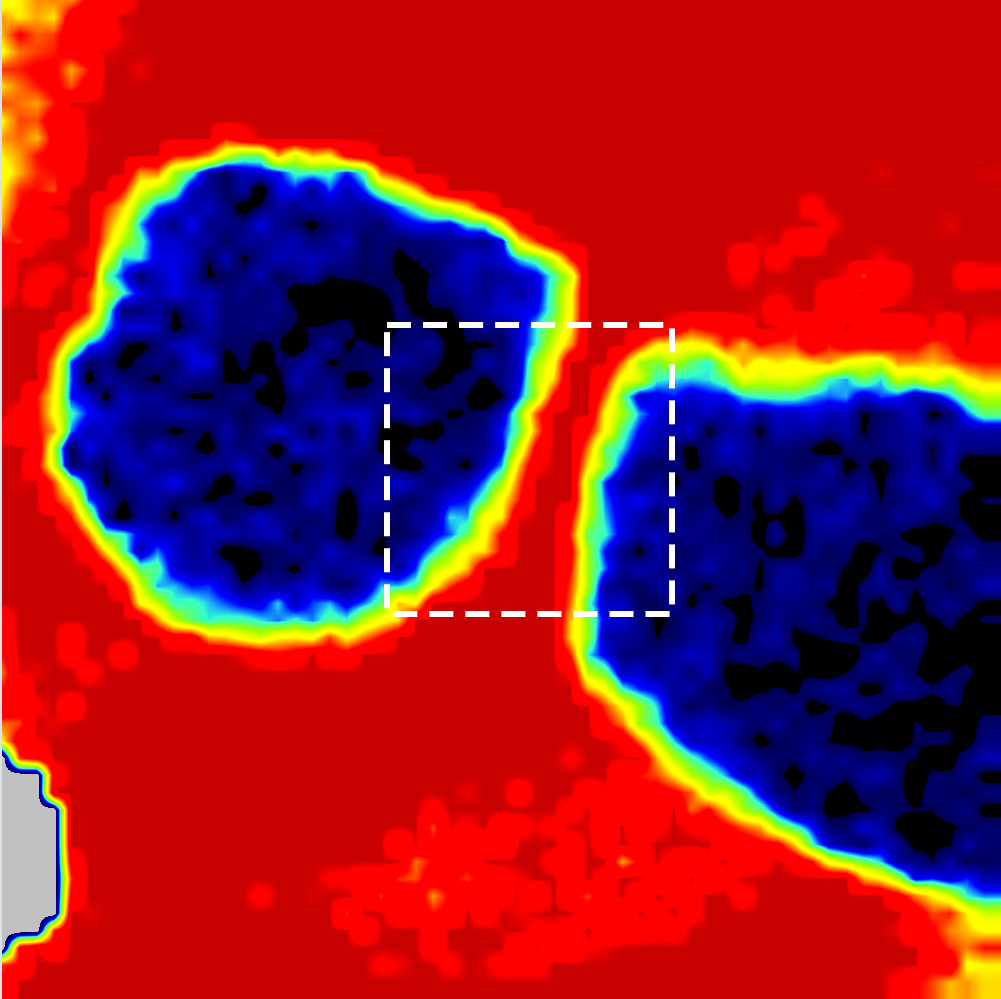

Supplement: Supplementary file 4 — Source Data Fig. 3 [file 44321_2024_49_MOESM4_ESM.zip › Figure 3/3E/sham/sham 0 min.tif]

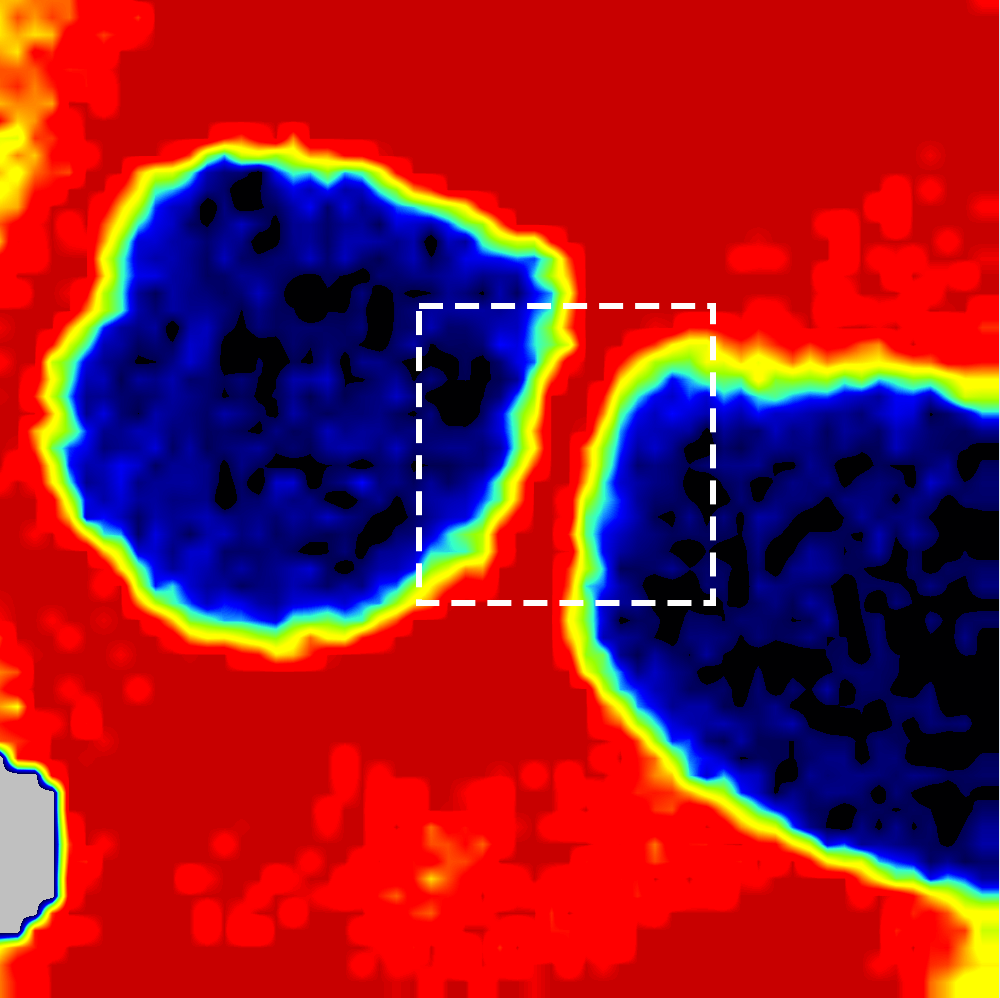

Supplement: Supplementary file 4 — Source Data Fig. 3 [file 44321_2024_49_MOESM4_ESM.zip › Figure 3/3E/sham/sham 10 min.tif]

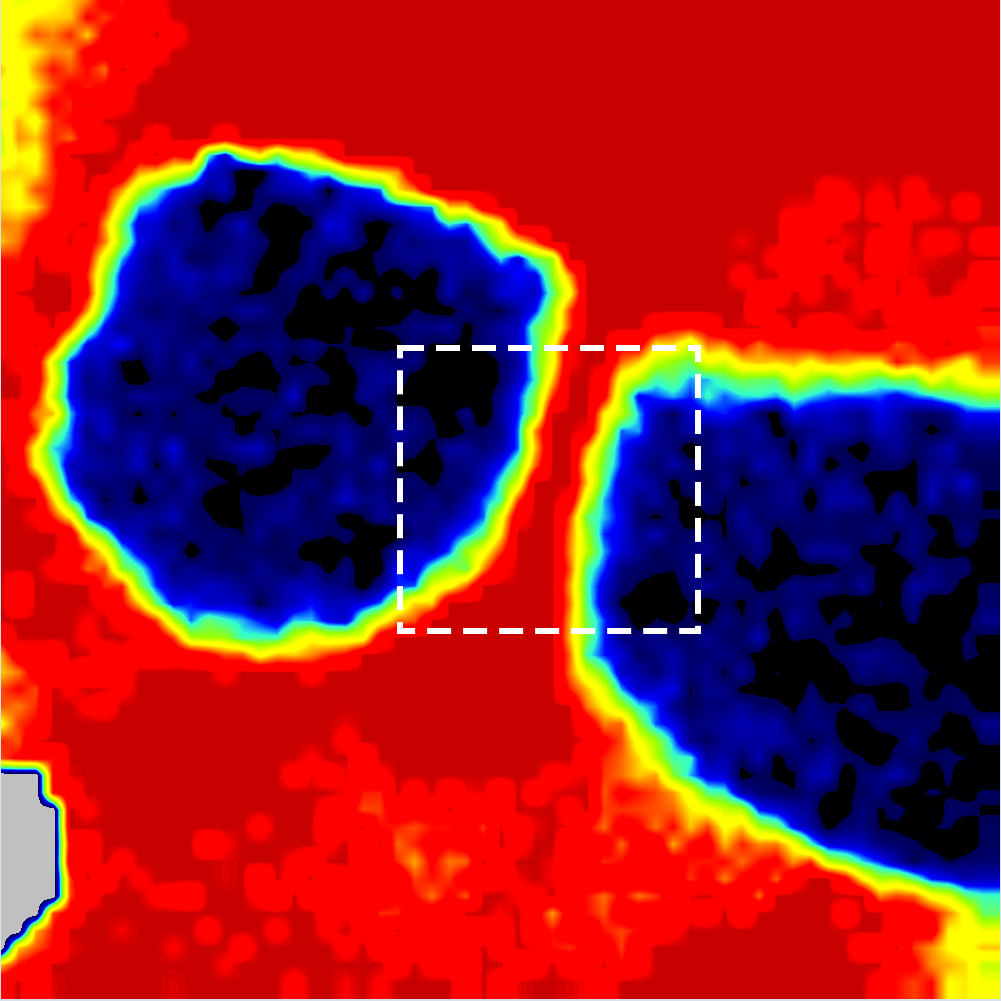

Supplement: Supplementary file 4 — Source Data Fig. 3 [file 44321_2024_49_MOESM4_ESM.zip › Figure 3/3E/sham/sham 15 min.tif]

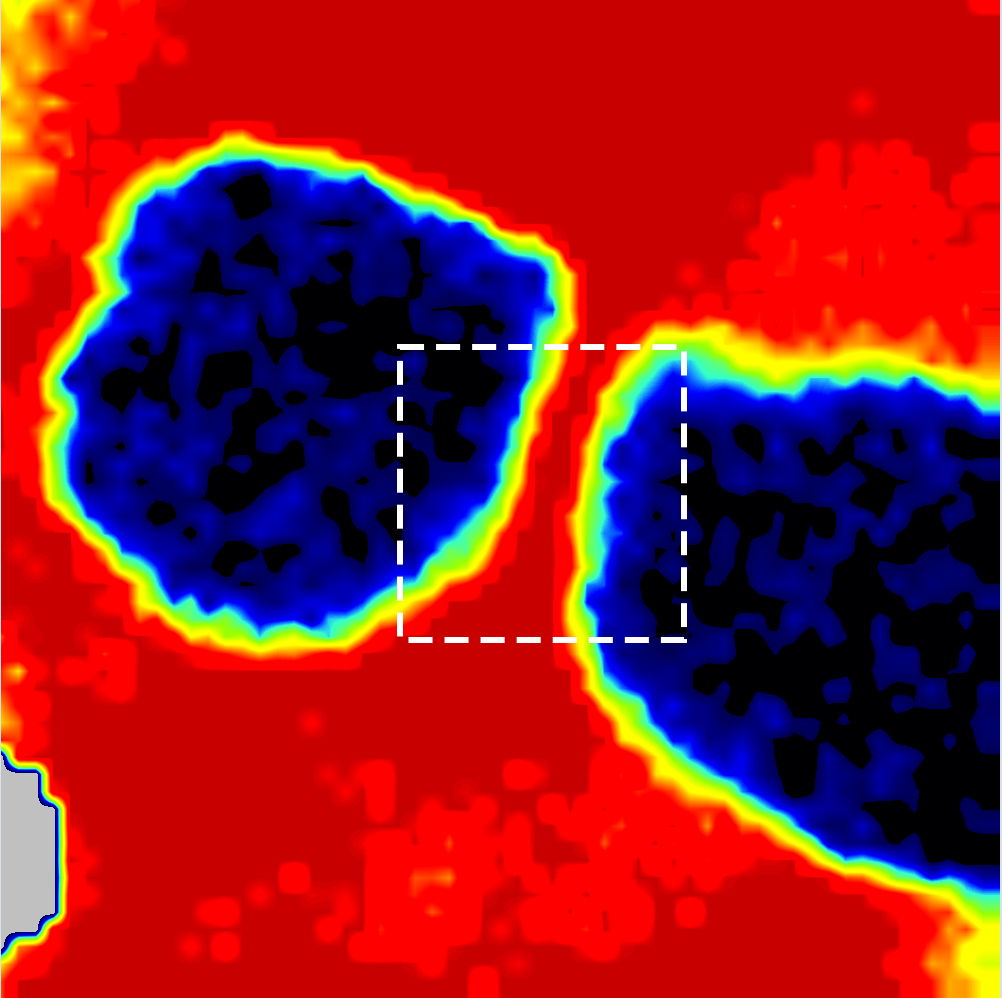

Supplement: Supplementary file 4 — Source Data Fig. 3 [file 44321_2024_49_MOESM4_ESM.zip › Figure 3/3E/sham/sham 5 min.tif]

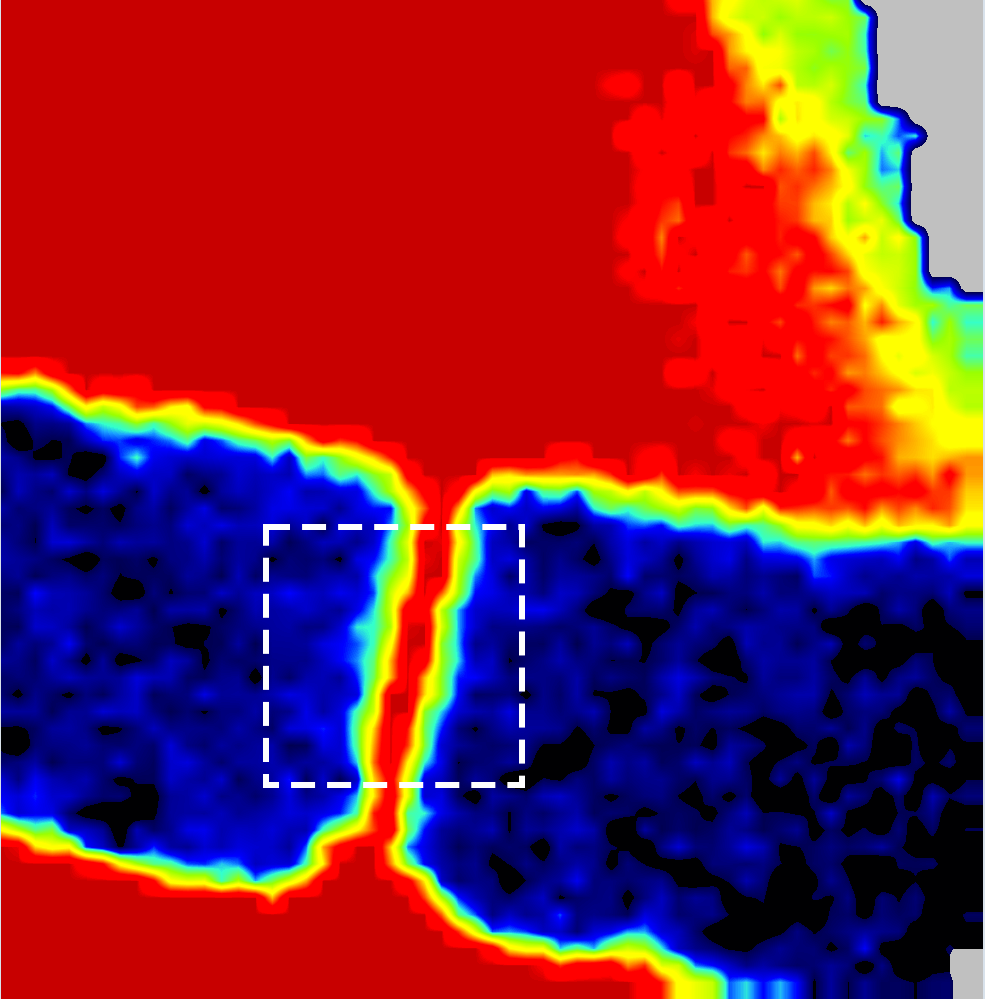

Supplement: Supplementary file 4 — Source Data Fig. 3 [file 44321_2024_49_MOESM4_ESM.zip › Figure 3/3E/N/Nu172 0min.tif]

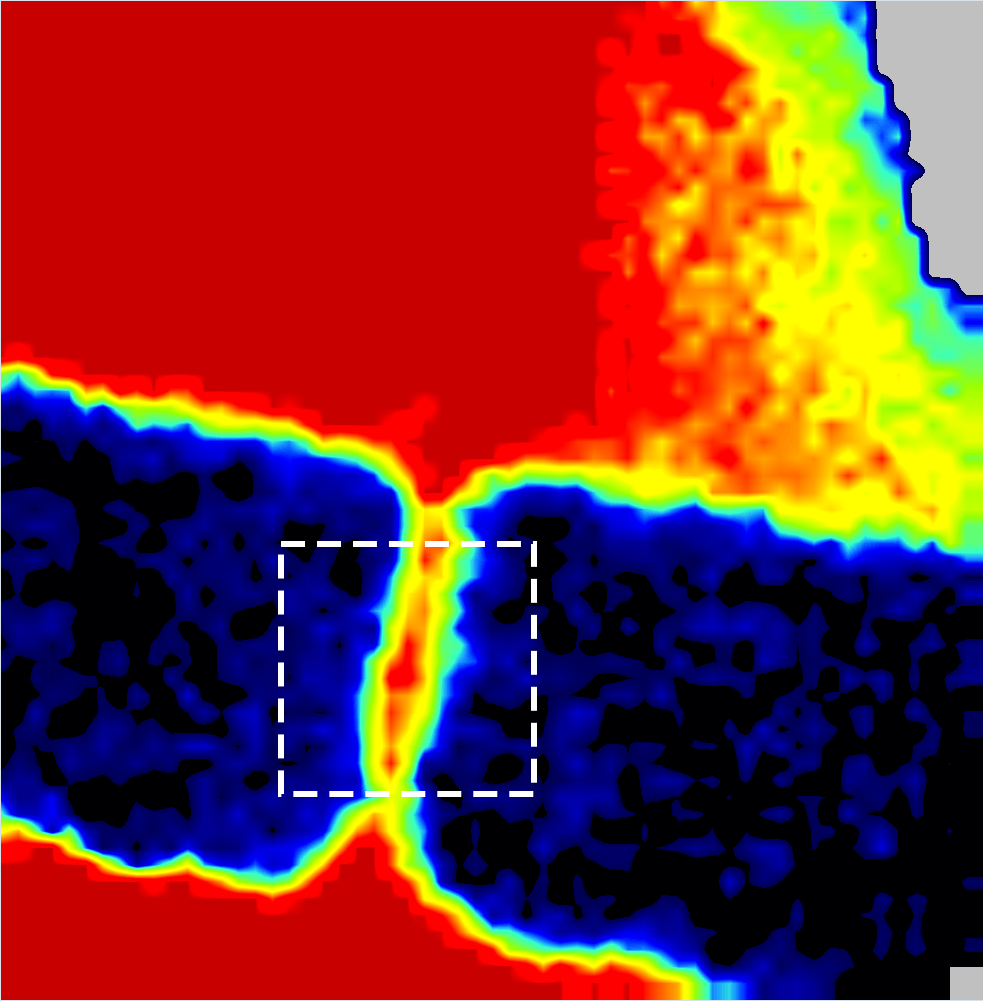

Supplement: Supplementary file 4 — Source Data Fig. 3 [file 44321_2024_49_MOESM4_ESM.zip › Figure 3/3E/N/Nu172 5 min.tif]

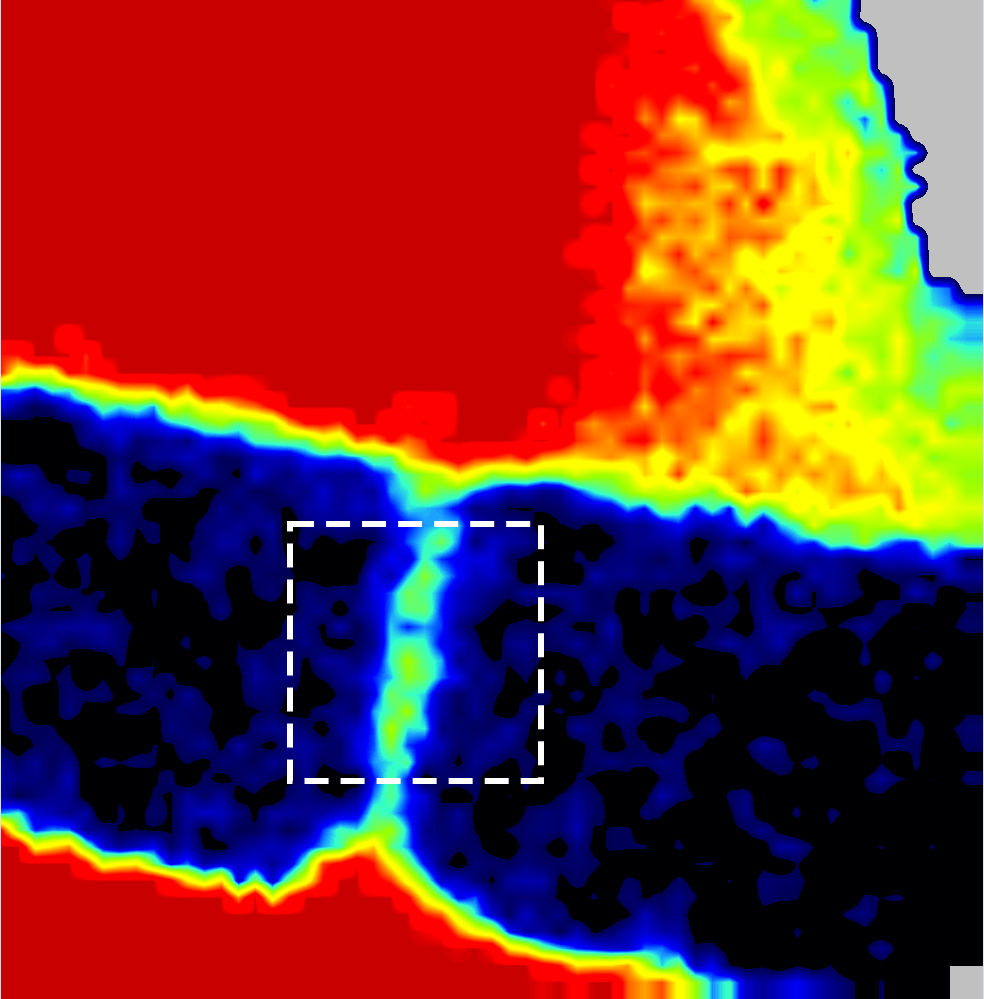

Supplement: Supplementary file 4 — Source Data Fig. 3 [file 44321_2024_49_MOESM4_ESM.zip › Figure 3/3E/N/Nu172 10 min 614-.tif]

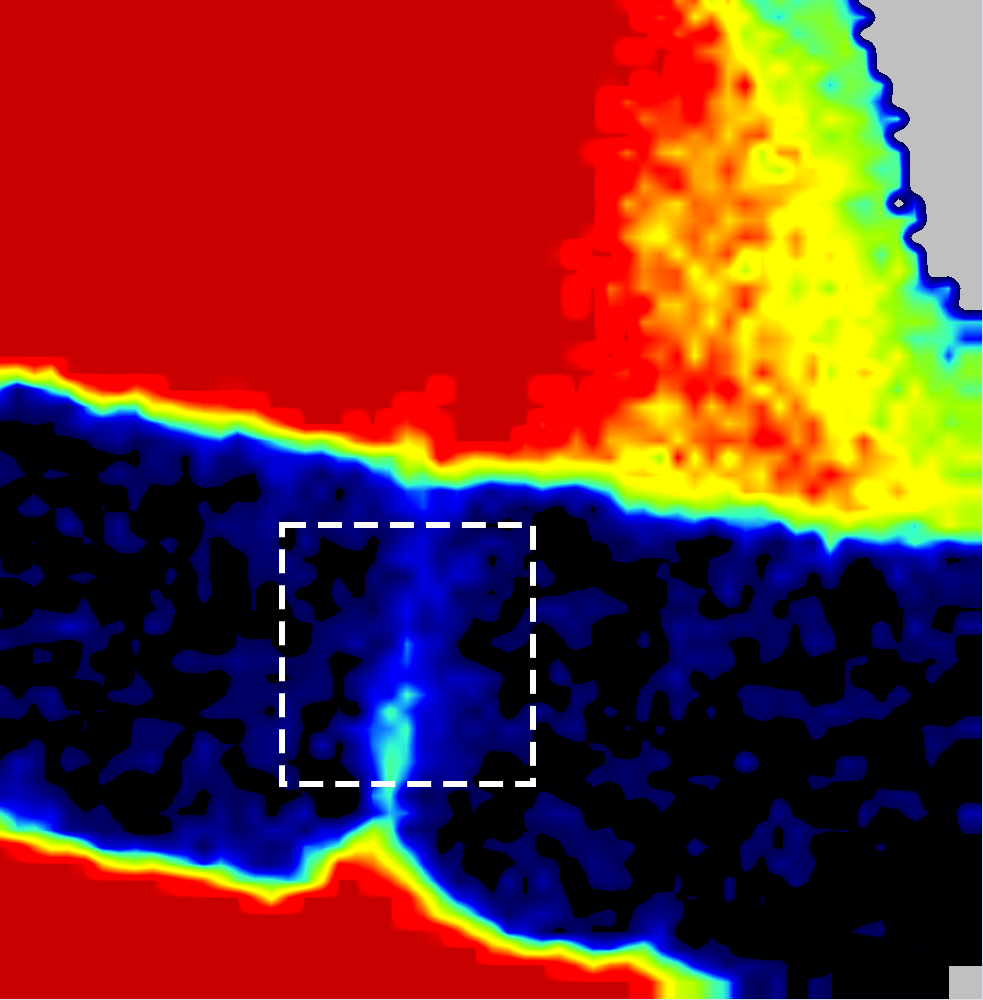

Supplement: Supplementary file 4 — Source Data Fig. 3 [file 44321_2024_49_MOESM4_ESM.zip › Figure 3/3E/N/Nu172 15 min .tif]

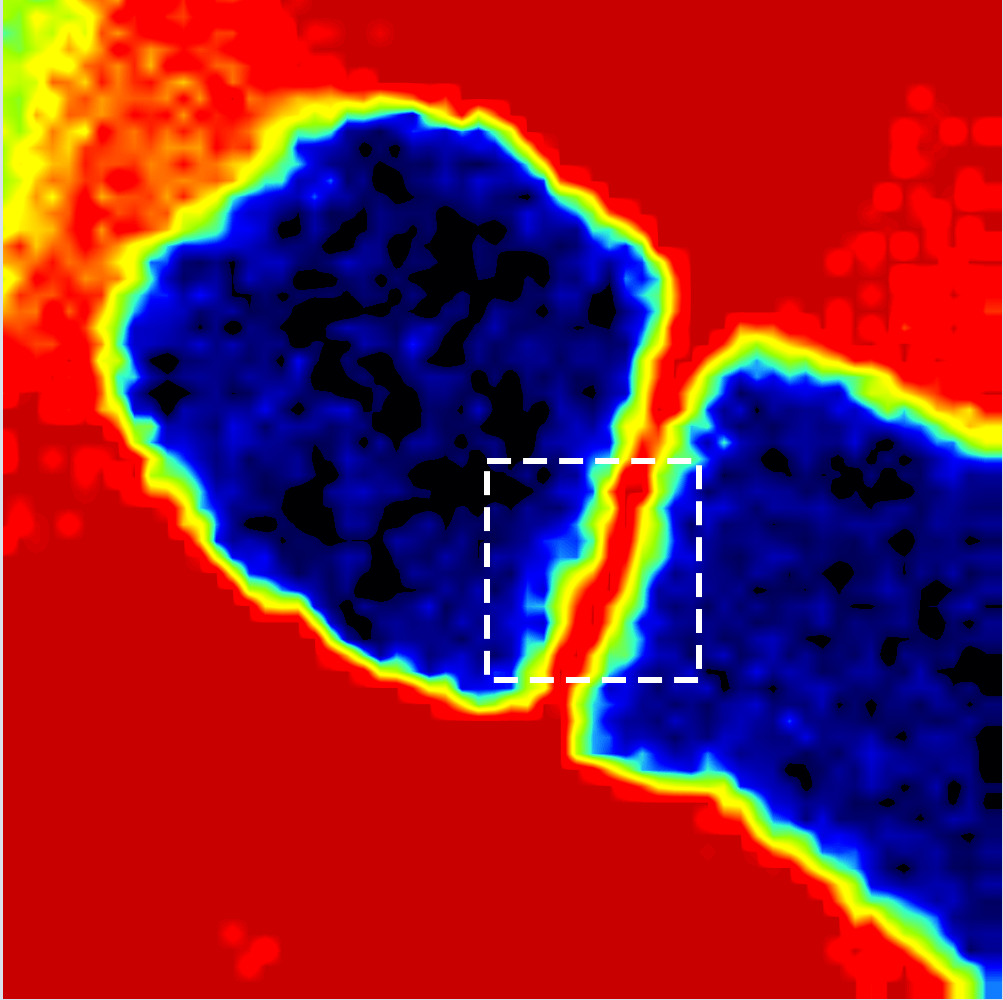

Supplement: Supplementary file 4 — Source Data Fig. 3 [file 44321_2024_49_MOESM4_ESM.zip › Figure 3/3E/Blank/blank control 0min.tif]

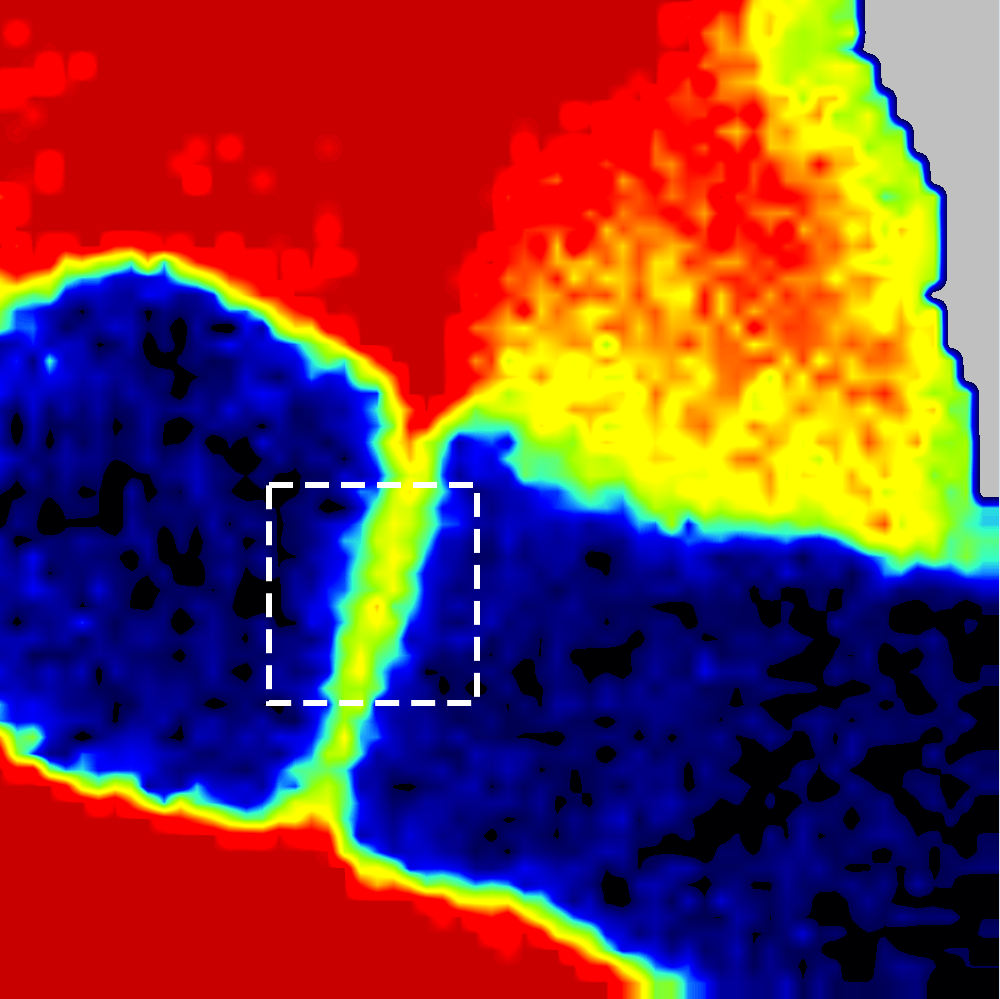

Supplement: Supplementary file 4 — Source Data Fig. 3 [file 44321_2024_49_MOESM4_ESM.zip › Figure 3/3E/Blank/blank control 10min 489.tif]

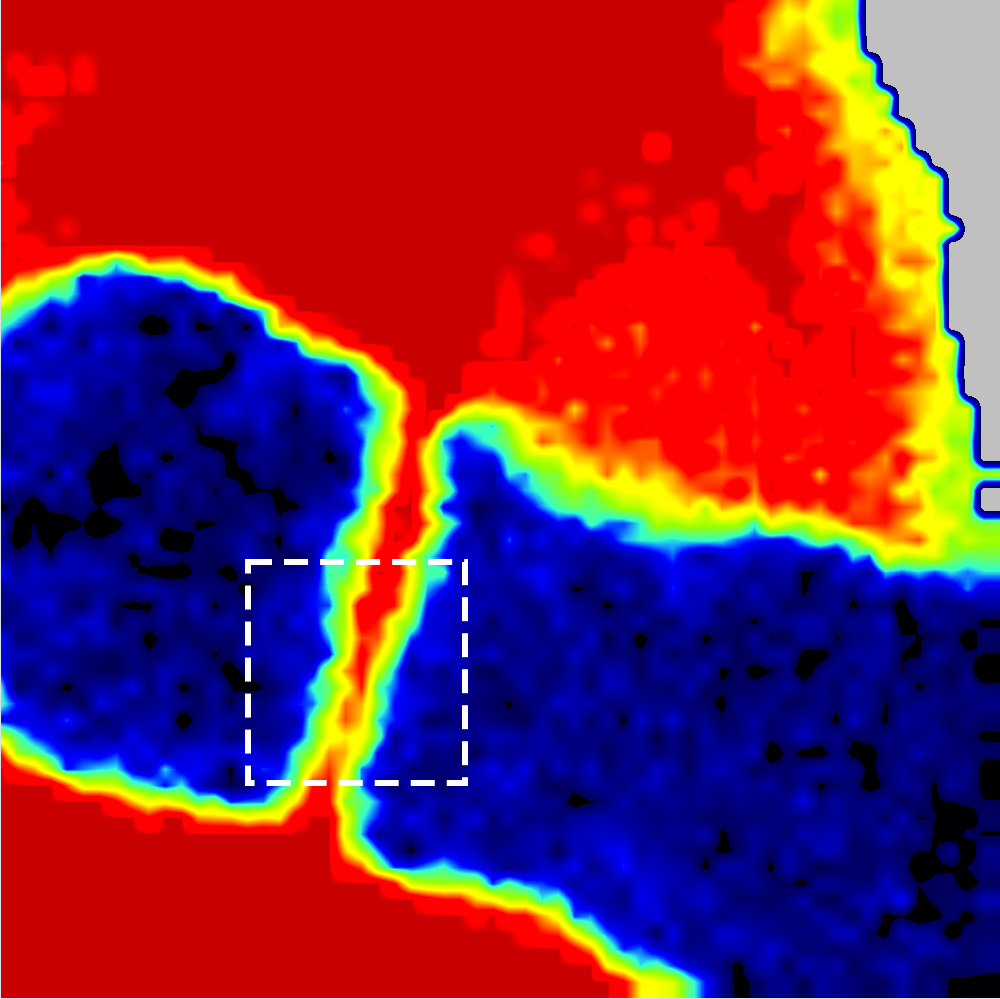

Supplement: Supplementary file 4 — Source Data Fig. 3 [file 44321_2024_49_MOESM4_ESM.zip › Figure 3/3E/Blank/blank control 5min 363.tif]

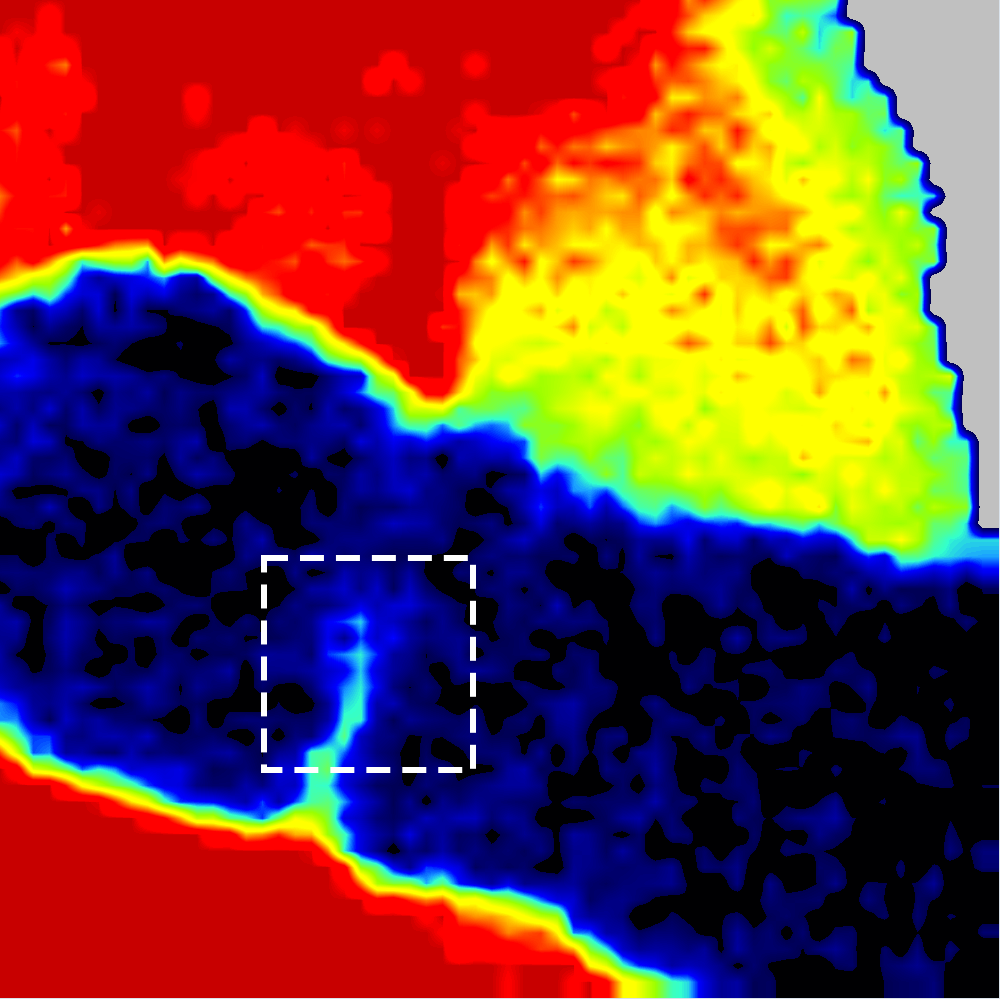

Supplement: Supplementary file 4 — Source Data Fig. 3 [file 44321_2024_49_MOESM4_ESM.zip › Figure 3/3E/Blank/blank control 15min.tif]

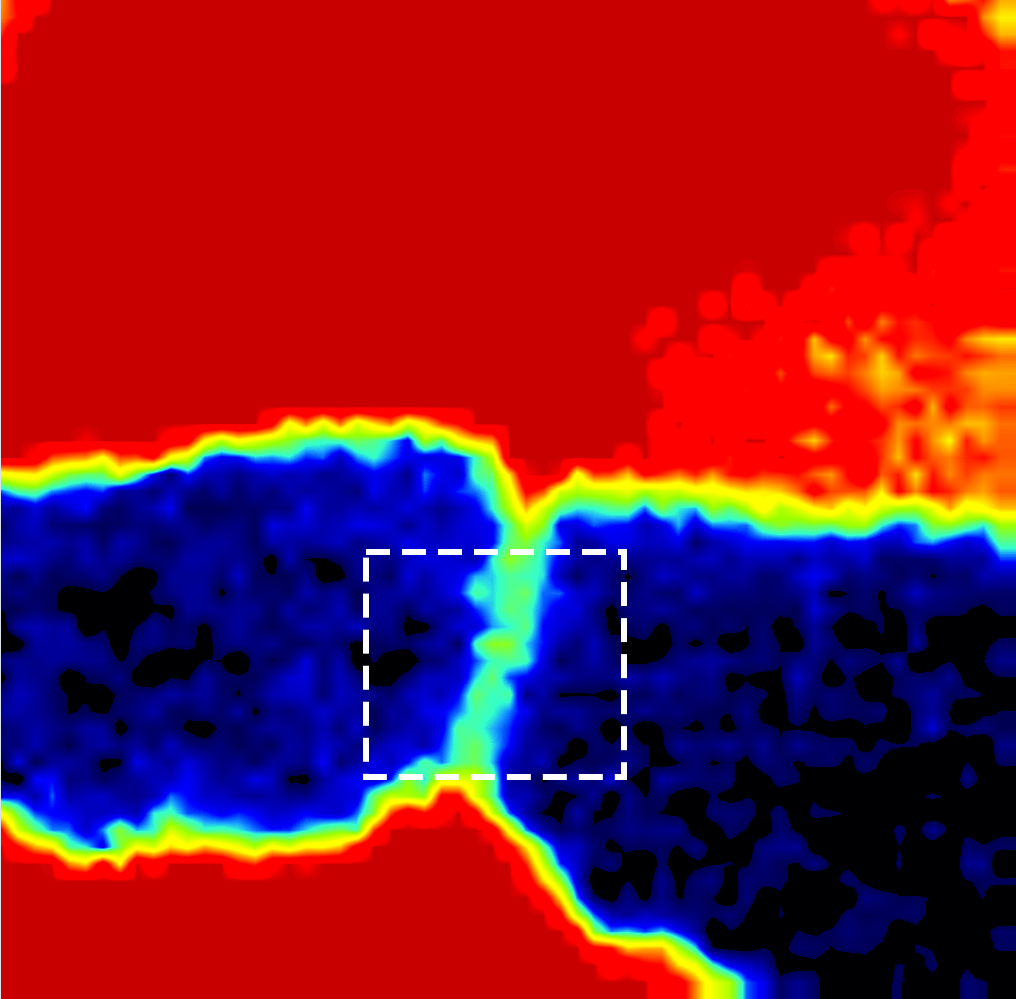

Supplement: Supplementary file 4 — Source Data Fig. 3 [file 44321_2024_49_MOESM4_ESM.zip › Figure 3/3E/EXO-N/EXO NU172 10min.tif]

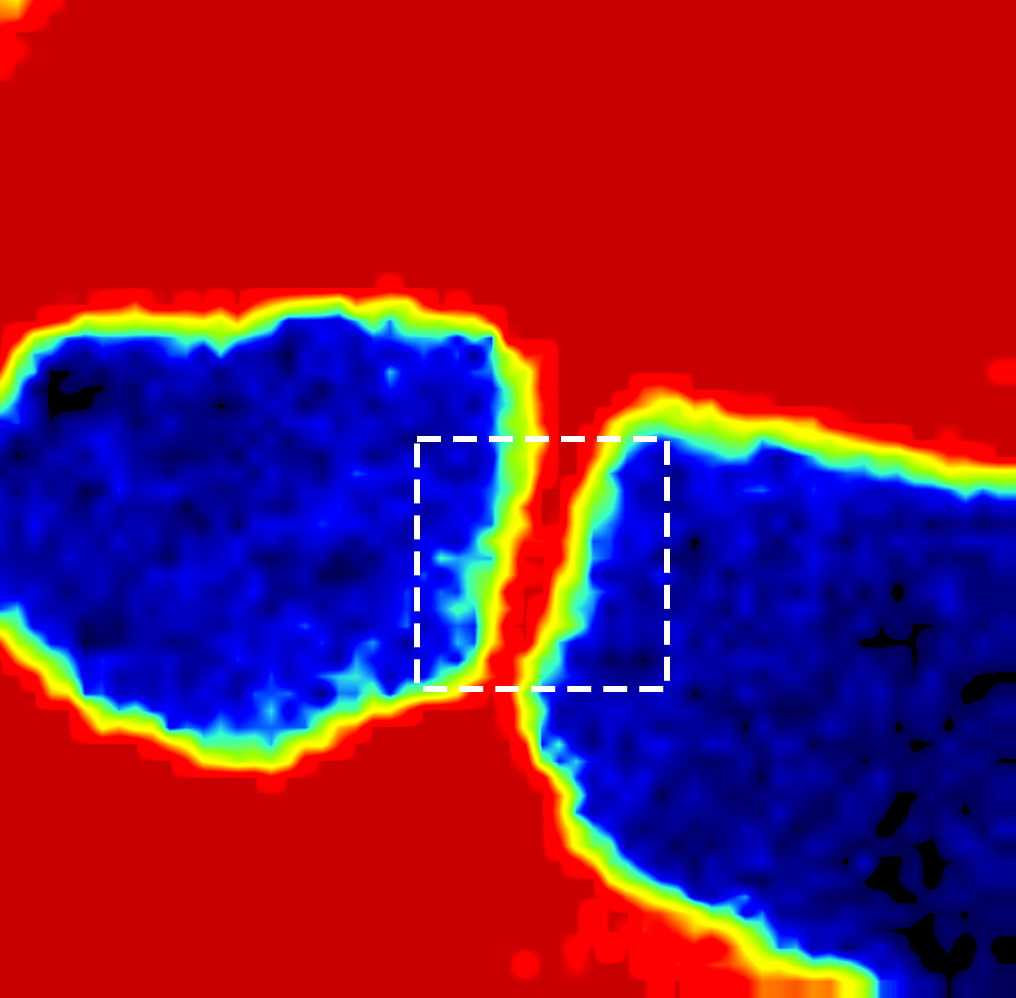

Supplement: Supplementary file 4 — Source Data Fig. 3 [file 44321_2024_49_MOESM4_ESM.zip › Figure 3/3E/EXO-N/EXO NU172 0min.tif]

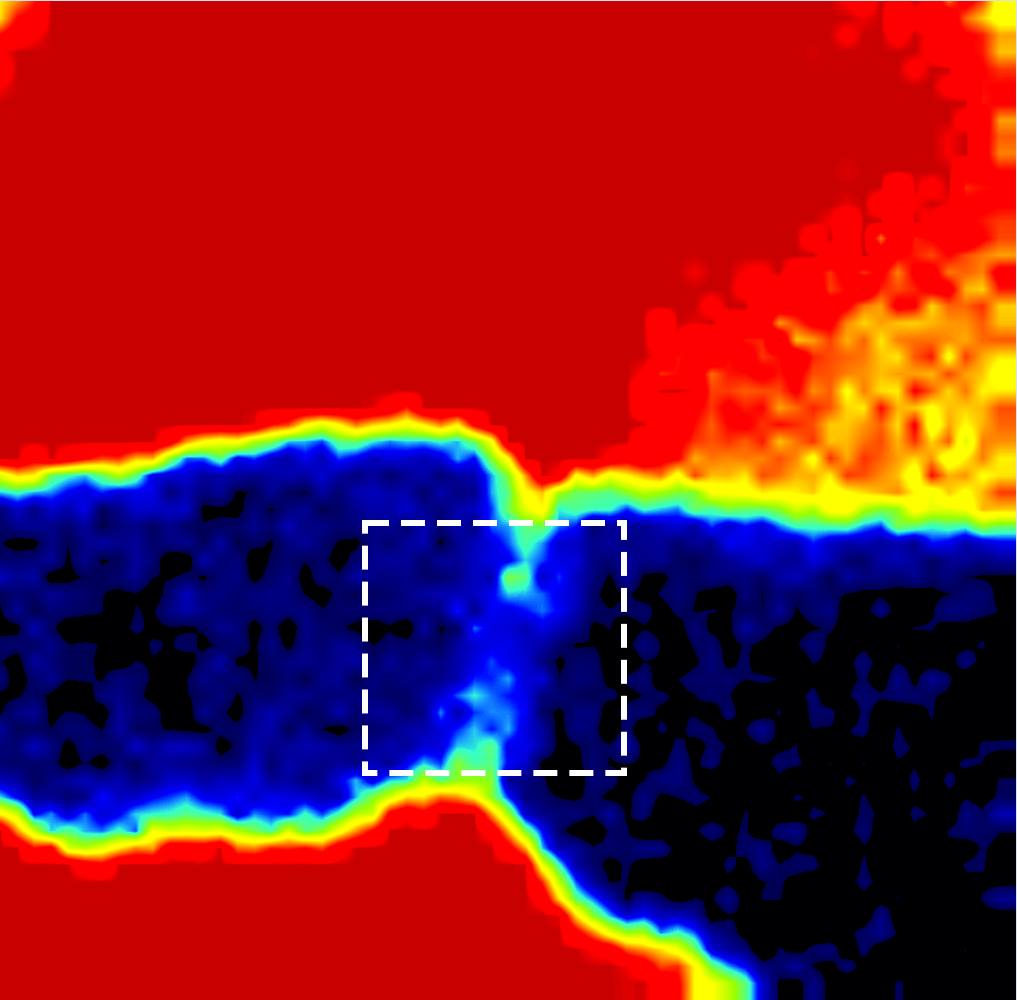

Supplement: Supplementary file 4 — Source Data Fig. 3 [file 44321_2024_49_MOESM4_ESM.zip › Figure 3/3E/EXO-N/EXO NU172 15min.tif]

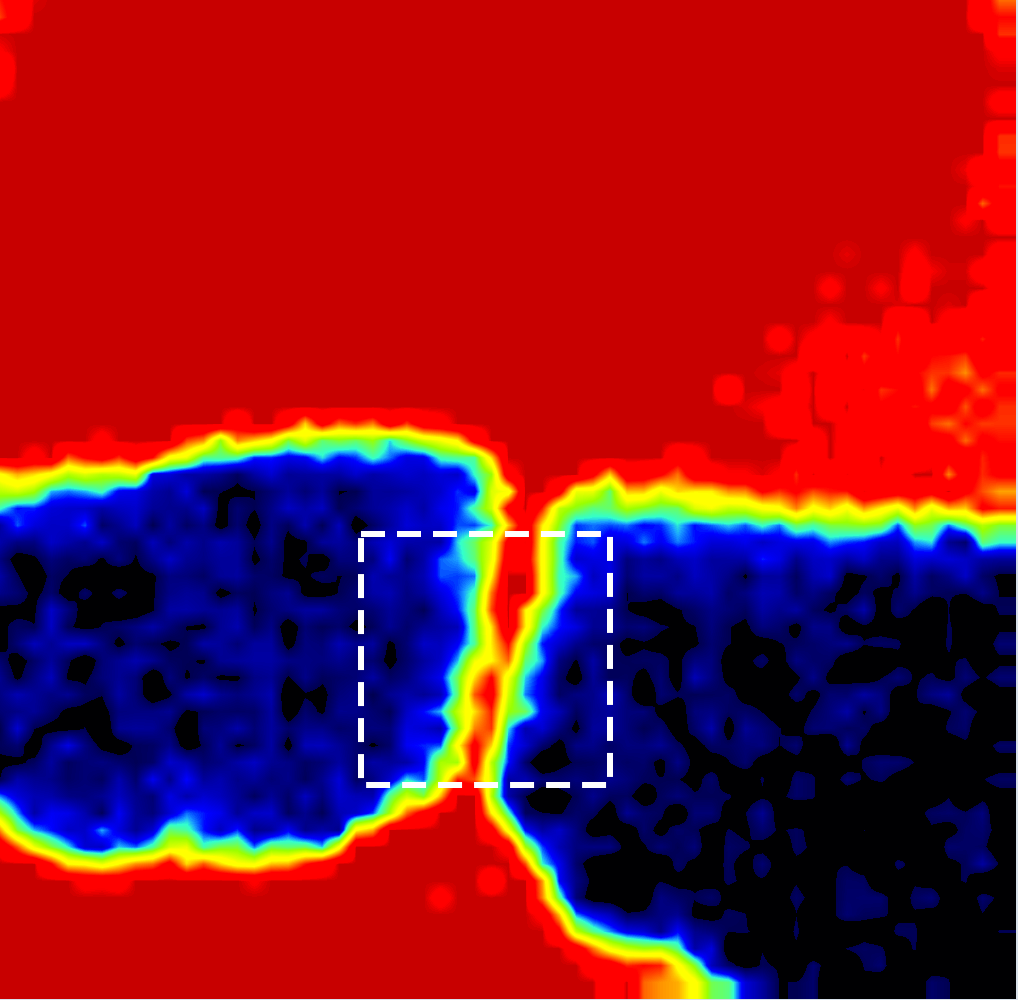

Supplement: Supplementary file 4 — Source Data Fig. 3 [file 44321_2024_49_MOESM4_ESM.zip › Figure 3/3E/EXO-N/EXO NU172 5min .tif]

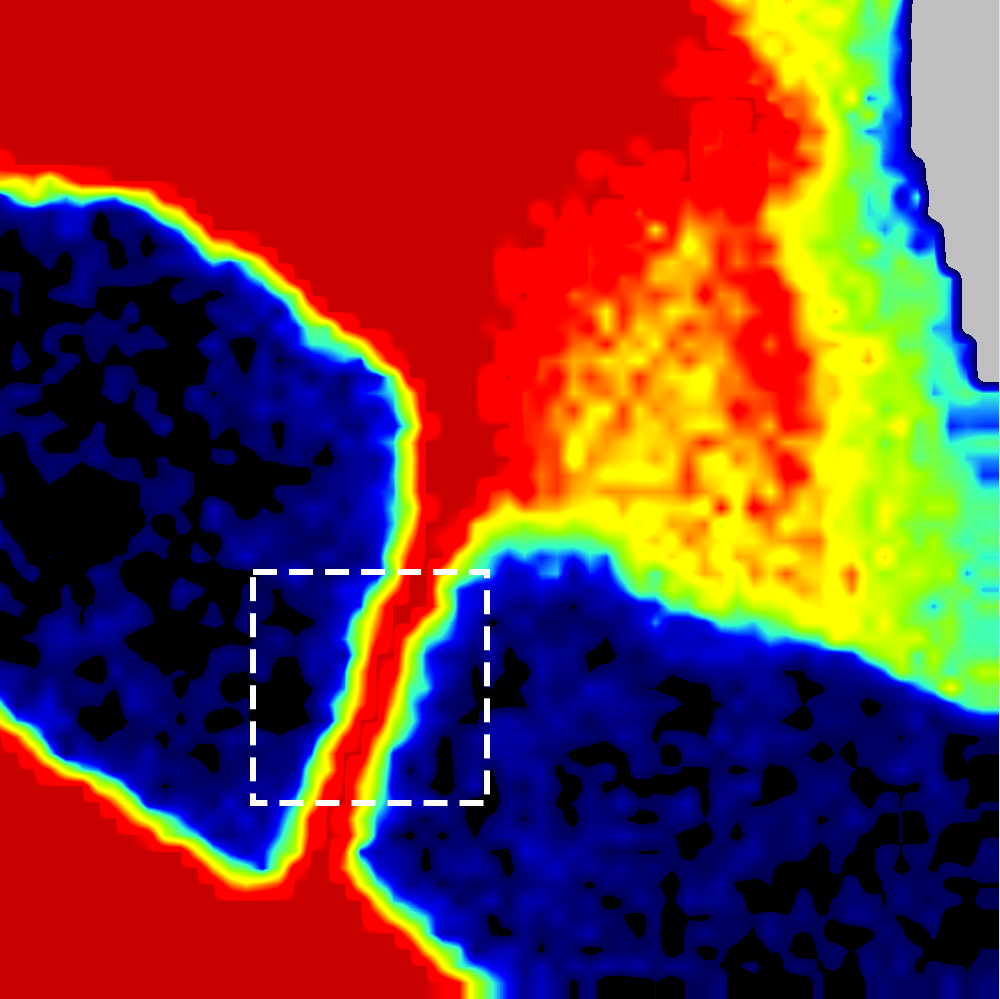

Supplement: Supplementary file 4 — Source Data Fig. 3 [file 44321_2024_49_MOESM4_ESM.zip › Figure 3/3E/EXOEAA-N/EXO-EAA-NU172 15min.tif]

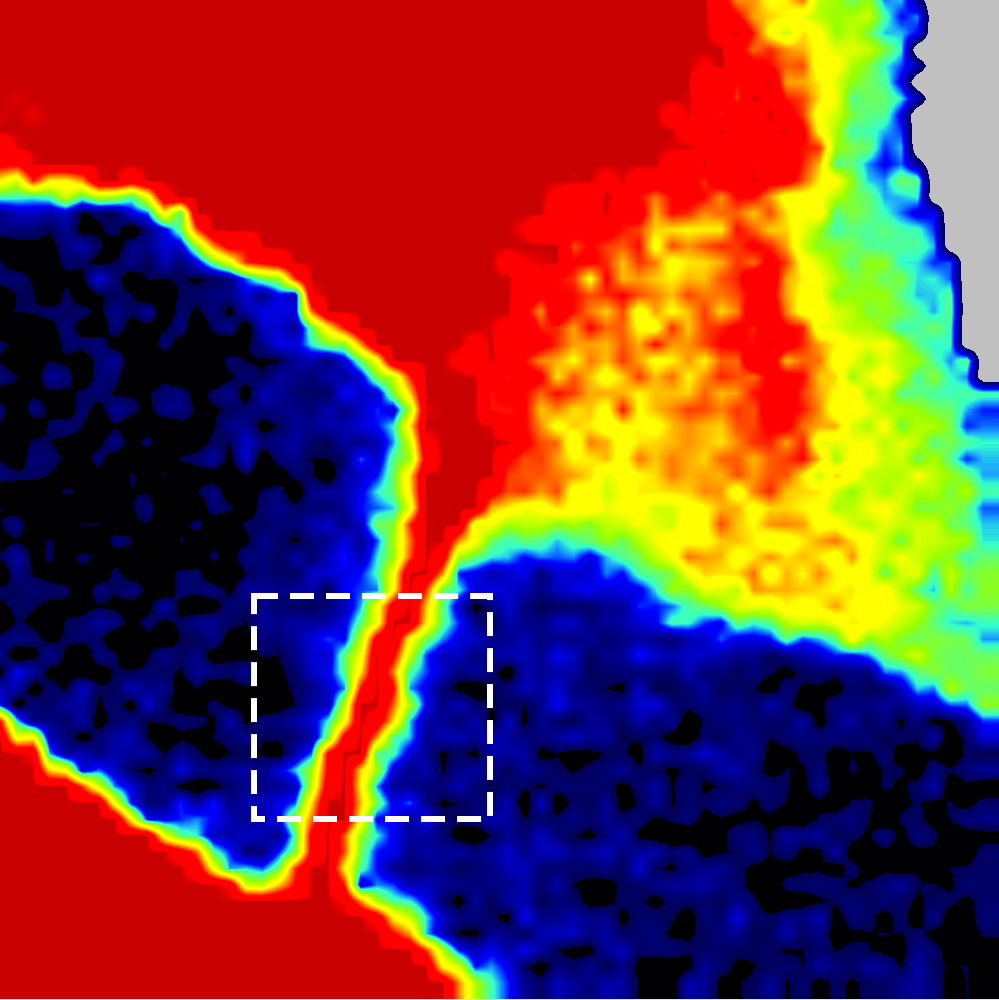

Supplement: Supplementary file 4 — Source Data Fig. 3 [file 44321_2024_49_MOESM4_ESM.zip › Figure 3/3E/EXOEAA-N/EXO-EAA-NU172 5min.tif]

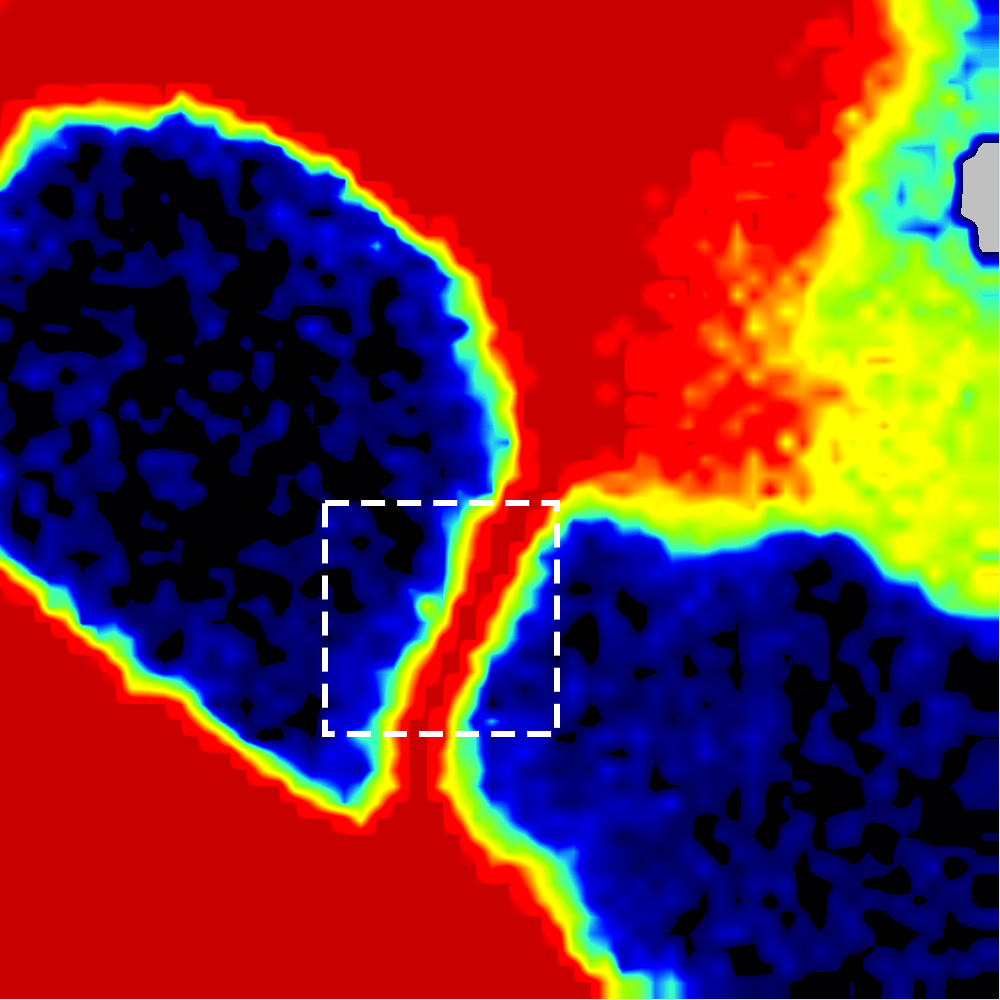

Supplement: Supplementary file 4 — Source Data Fig. 3 [file 44321_2024_49_MOESM4_ESM.zip › Figure 3/3E/EXOEAA-N/EXO-EAA-NU172 0min.tif]

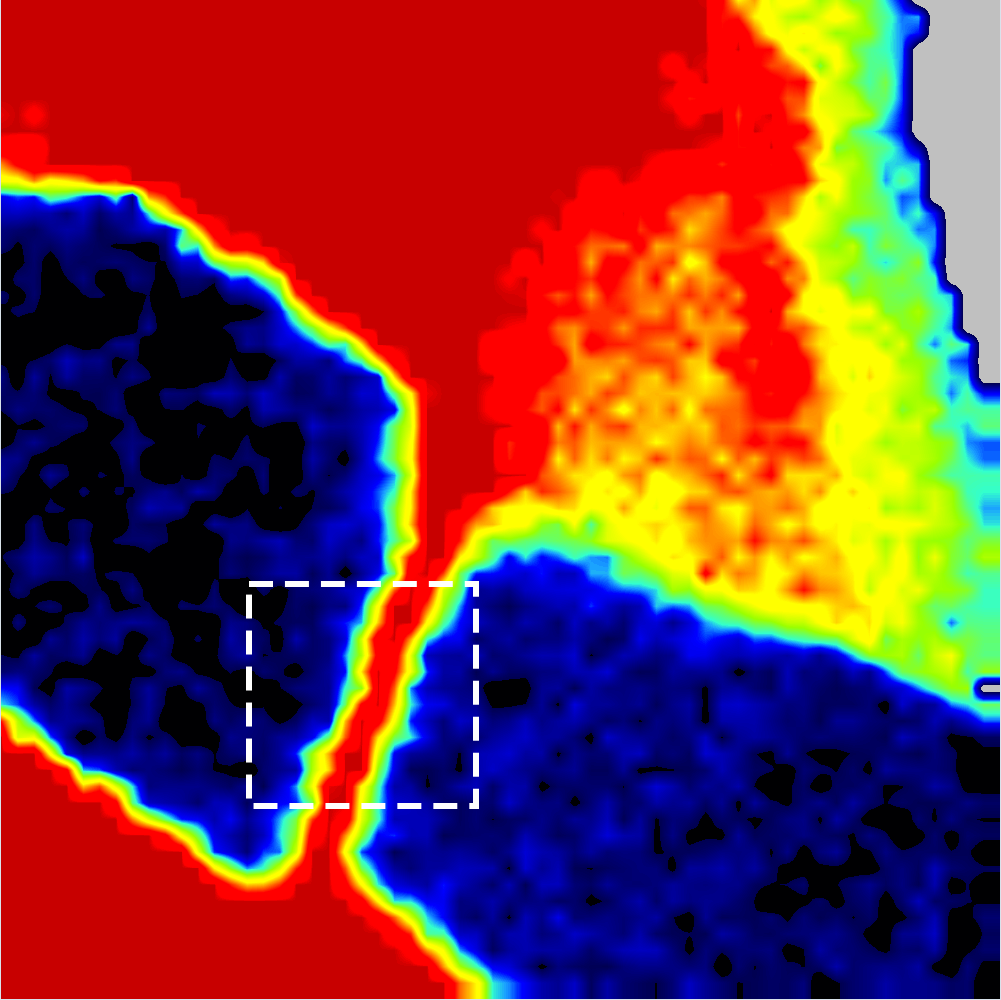

Supplement: Supplementary file 4 — Source Data Fig. 3 [file 44321_2024_49_MOESM4_ESM.zip › Figure 3/3E/EXOEAA-N/EXO-EAA-NU172 10min.tif]

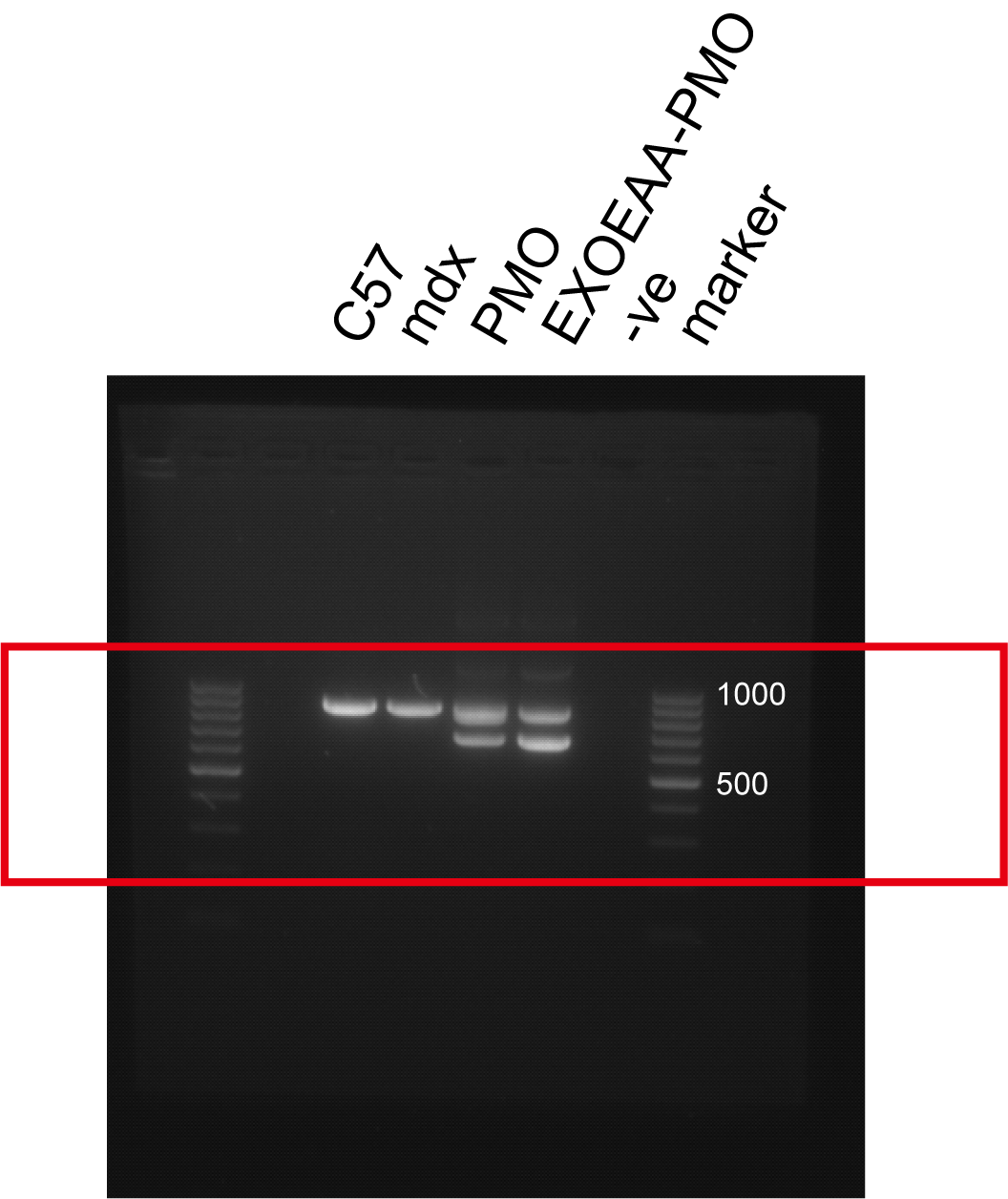

Supplement: Supplementary file 5 — Source Data Fig. 4 [file 44321_2024_49_MOESM5_ESM.zip › Figure 4/4E/4E.tif]

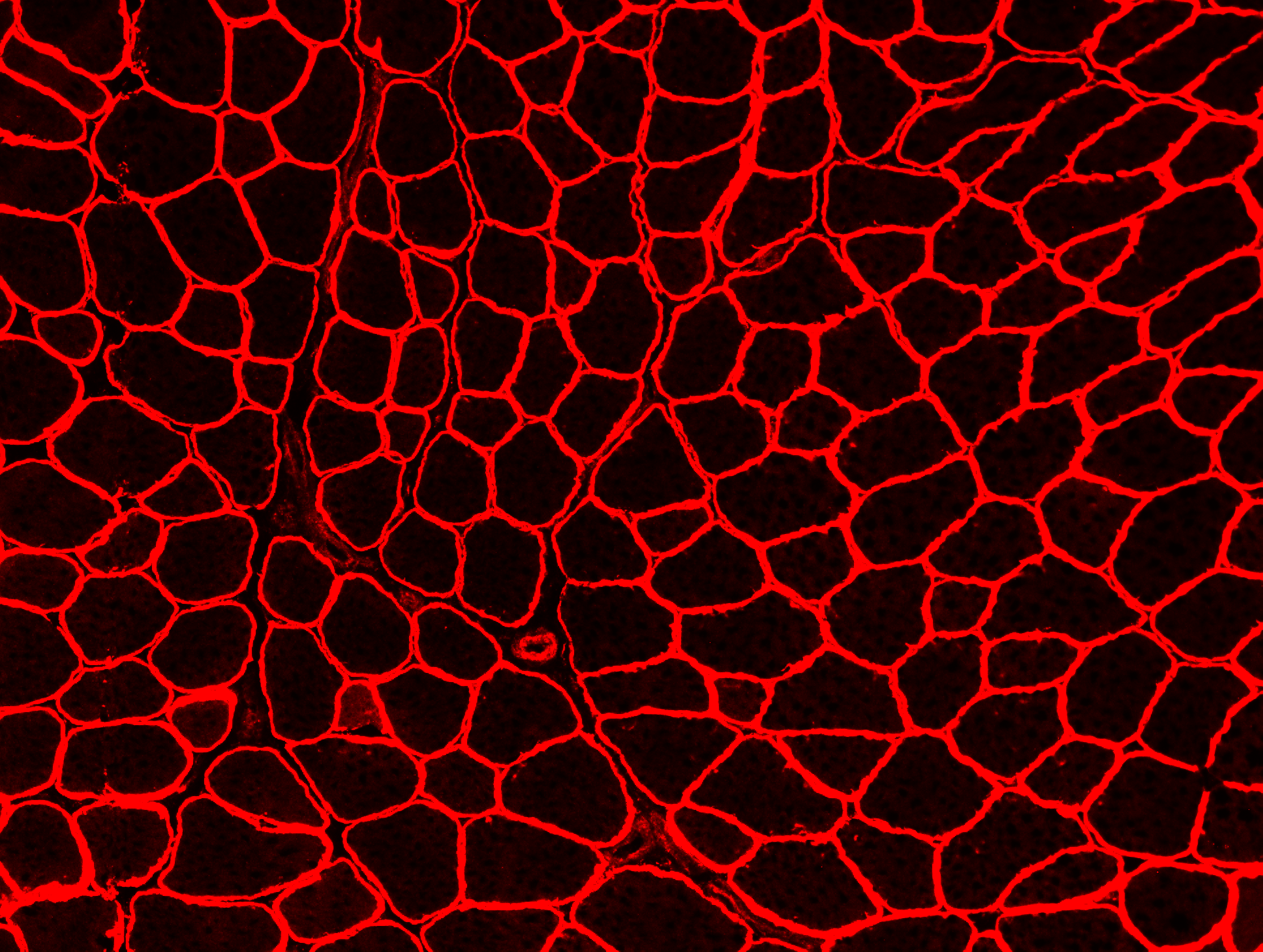

Supplement: Supplementary file 5 — Source Data Fig. 4 [file 44321_2024_49_MOESM5_ESM.zip › Figure 4/4D/C57 DYS.tif]

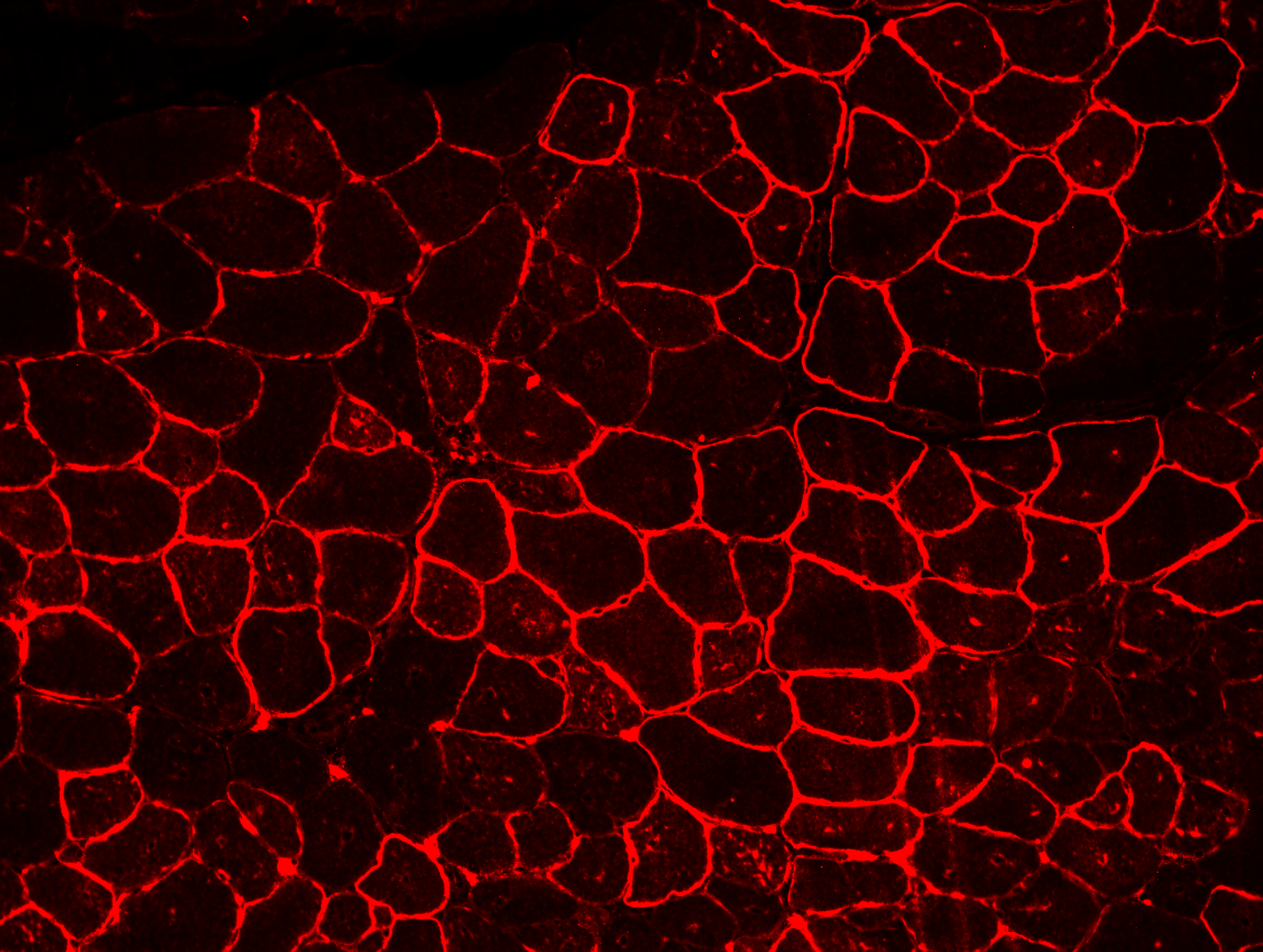

Supplement: Supplementary file 5 — Source Data Fig. 4 [file 44321_2024_49_MOESM5_ESM.zip › Figure 4/4D/EXOEAA-PMO DYS.tif]

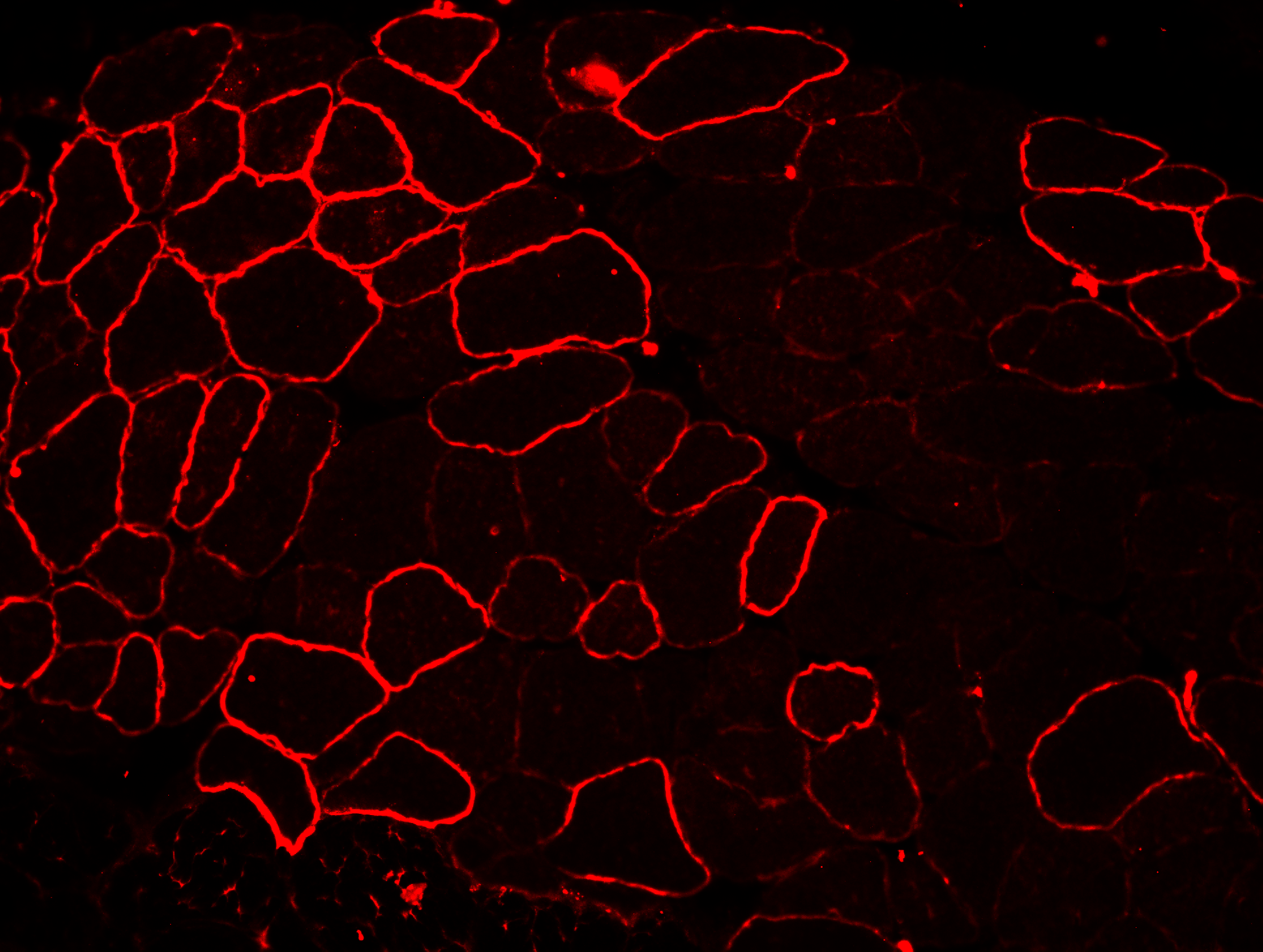

Supplement: Supplementary file 5 — Source Data Fig. 4 [file 44321_2024_49_MOESM5_ESM.zip › Figure 4/4D/PMO DYS.tif]

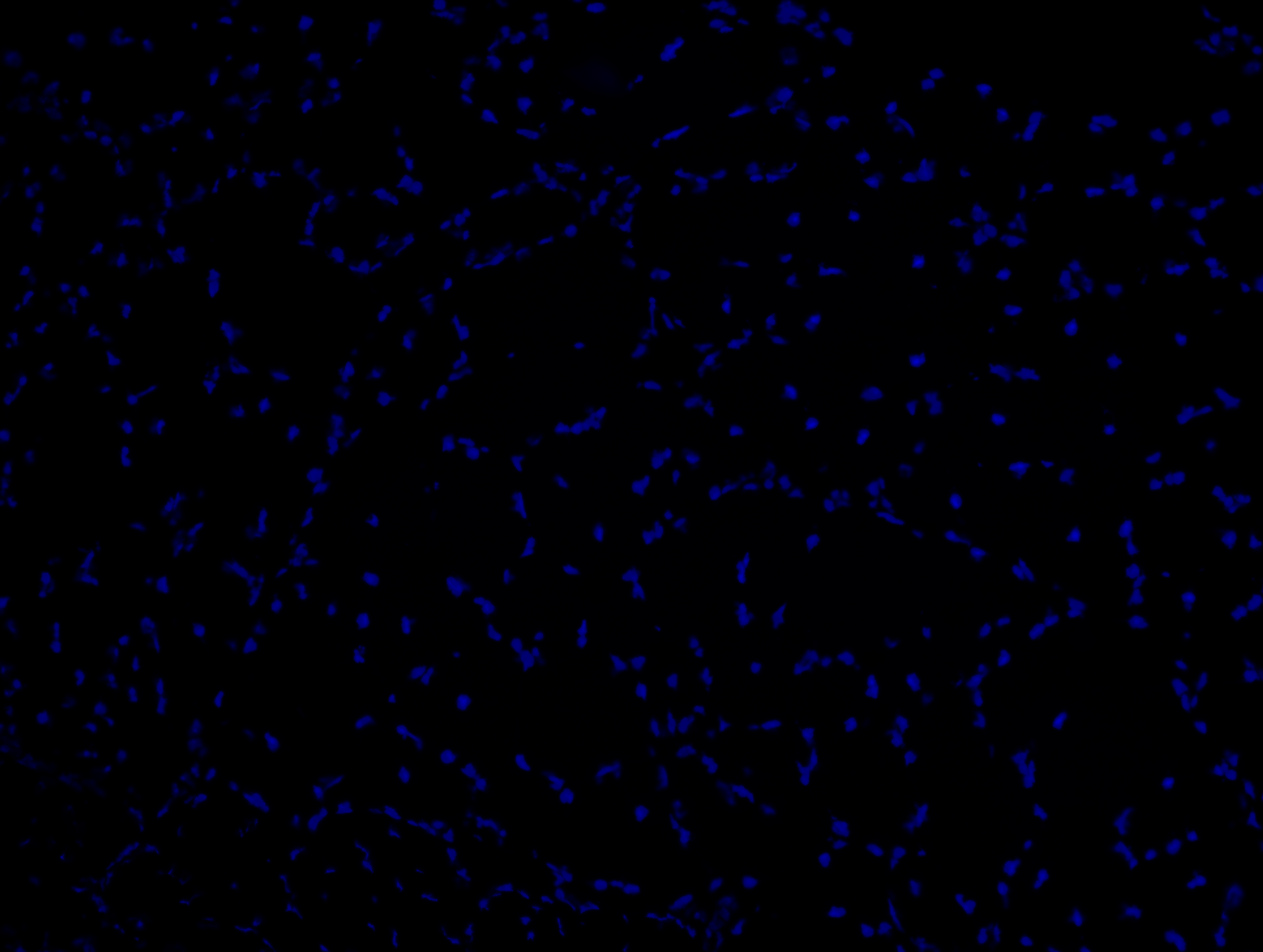

Supplement: Supplementary file 5 — Source Data Fig. 4 [file 44321_2024_49_MOESM5_ESM.zip › Figure 4/4D/PMO DAPI.tif]

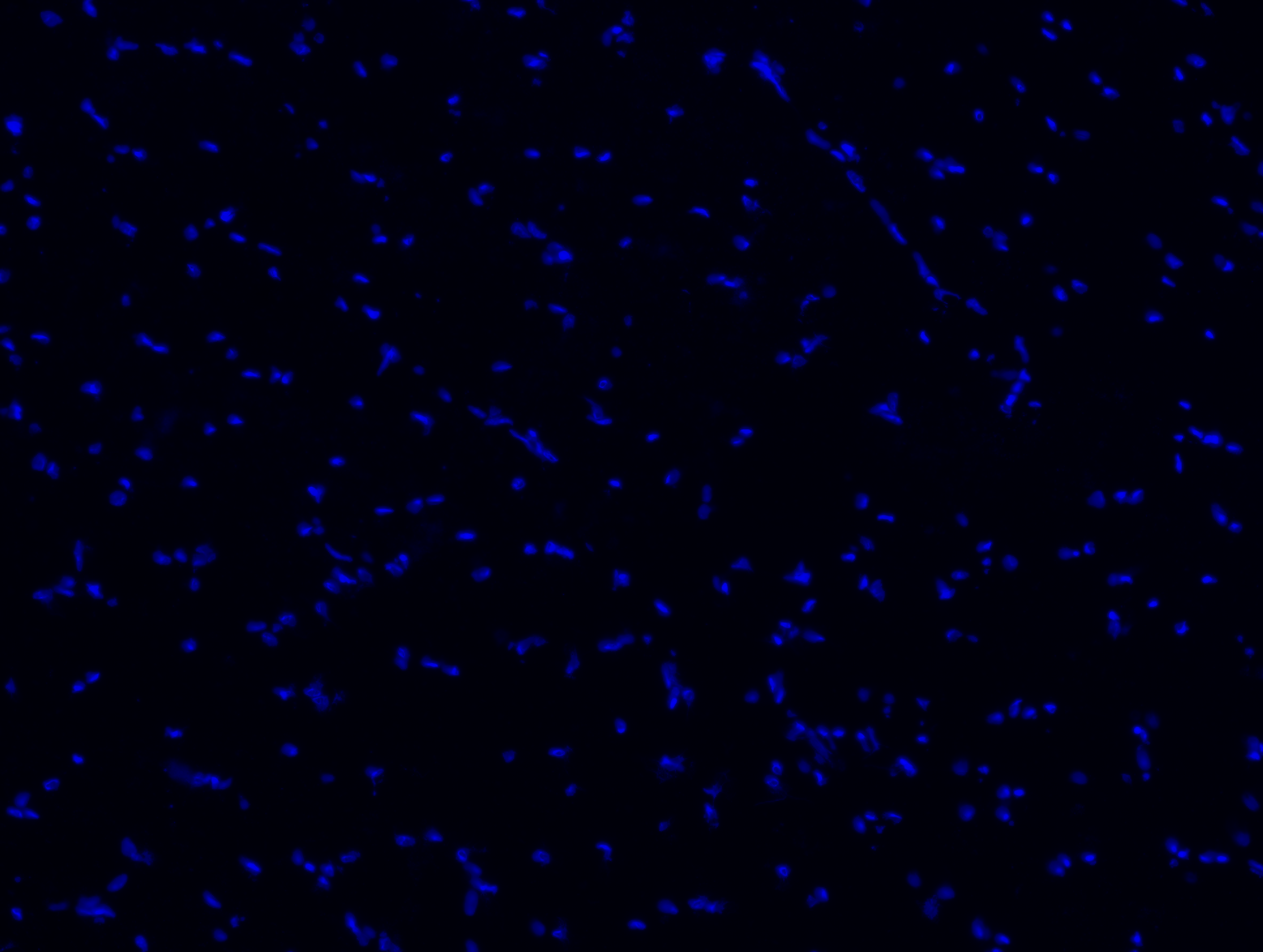

Supplement: Supplementary file 5 — Source Data Fig. 4 [file 44321_2024_49_MOESM5_ESM.zip › Figure 4/4D/mdx DYS.tif]

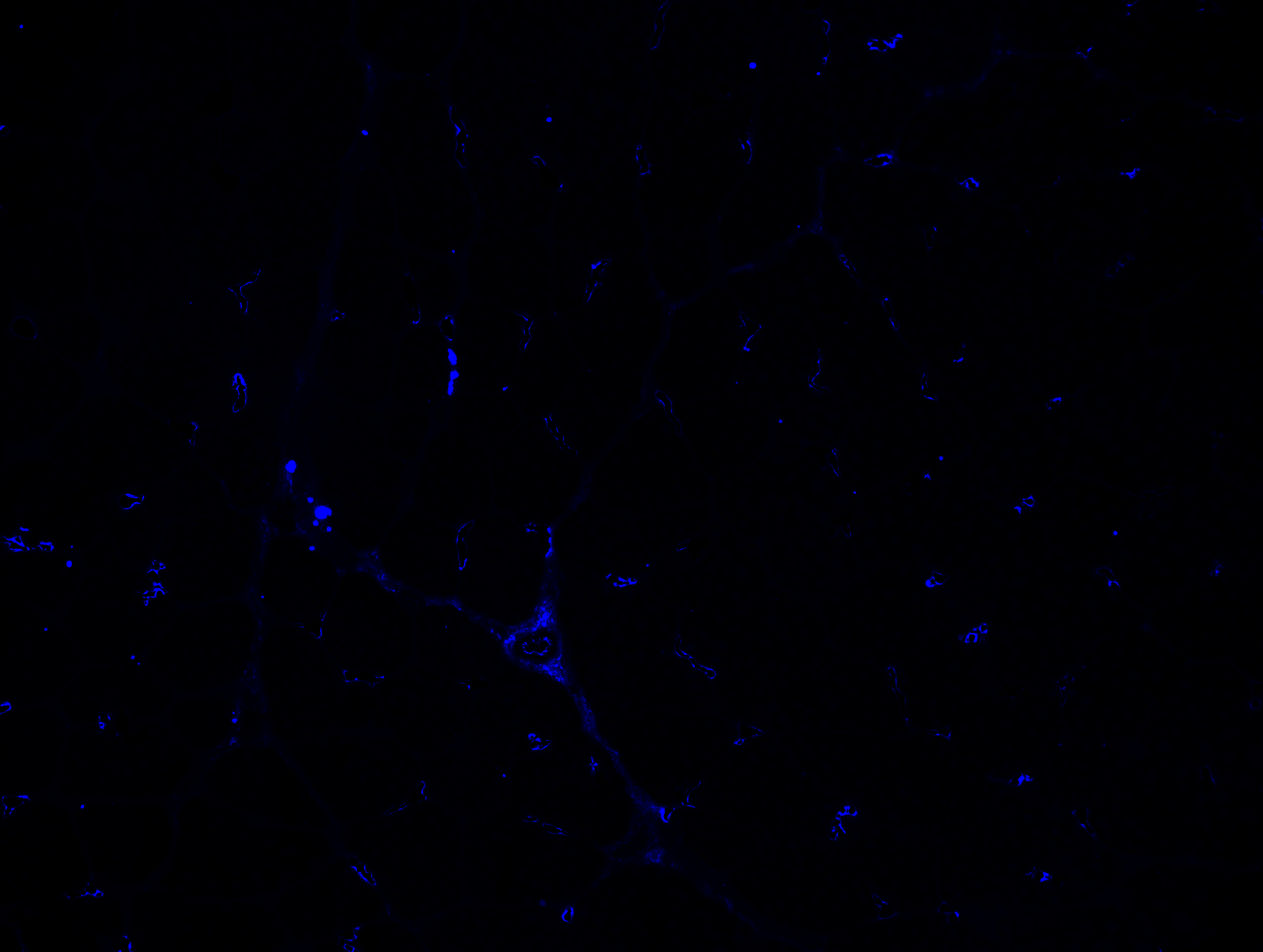

Supplement: Supplementary file 5 — Source Data Fig. 4 [file 44321_2024_49_MOESM5_ESM.zip › Figure 4/4D/C57 DAPI.tif]

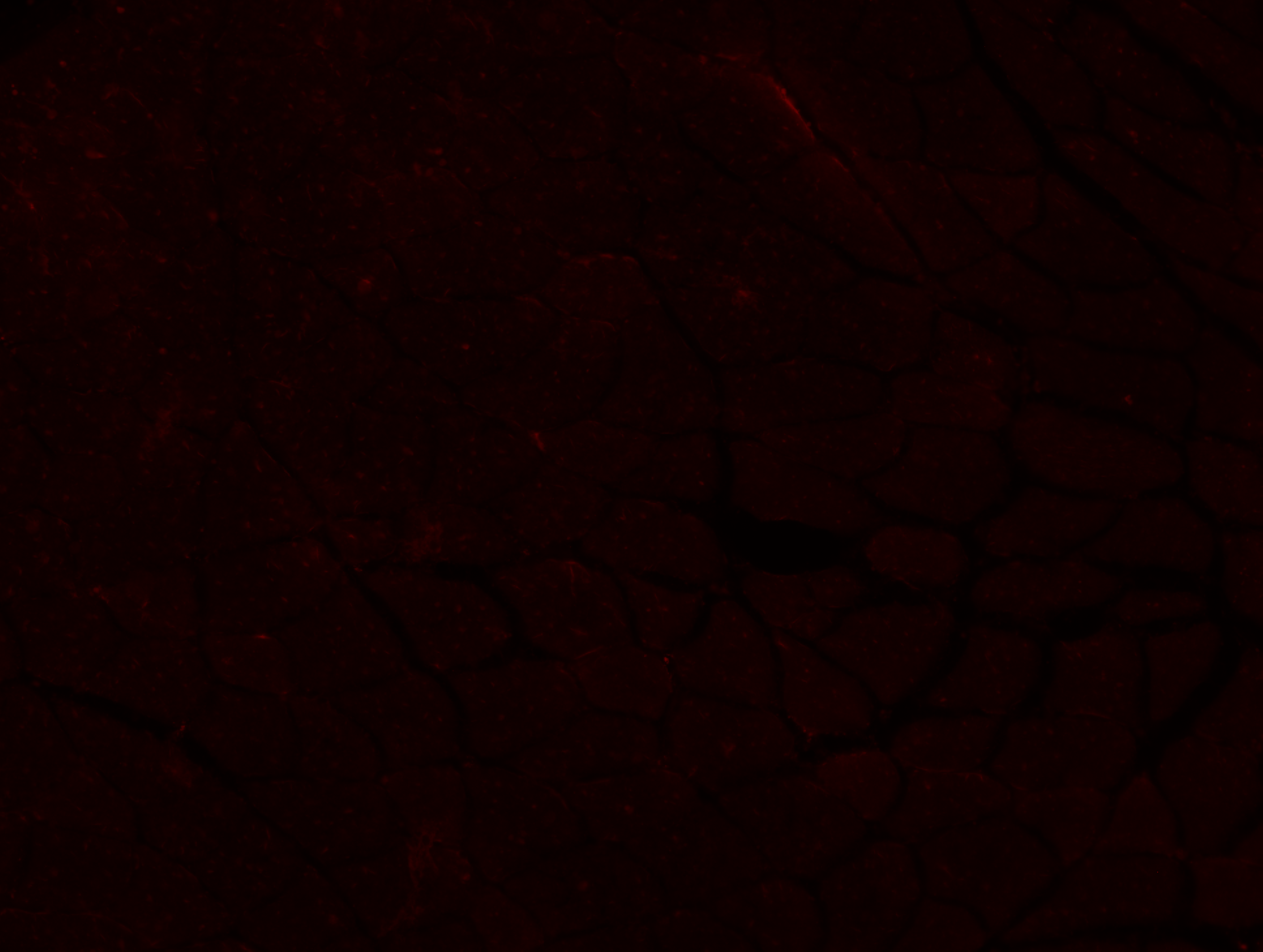

Supplement: Supplementary file 5 — Source Data Fig. 4 [file 44321_2024_49_MOESM5_ESM.zip › Figure 4/4D/mdx DAPI.tif]

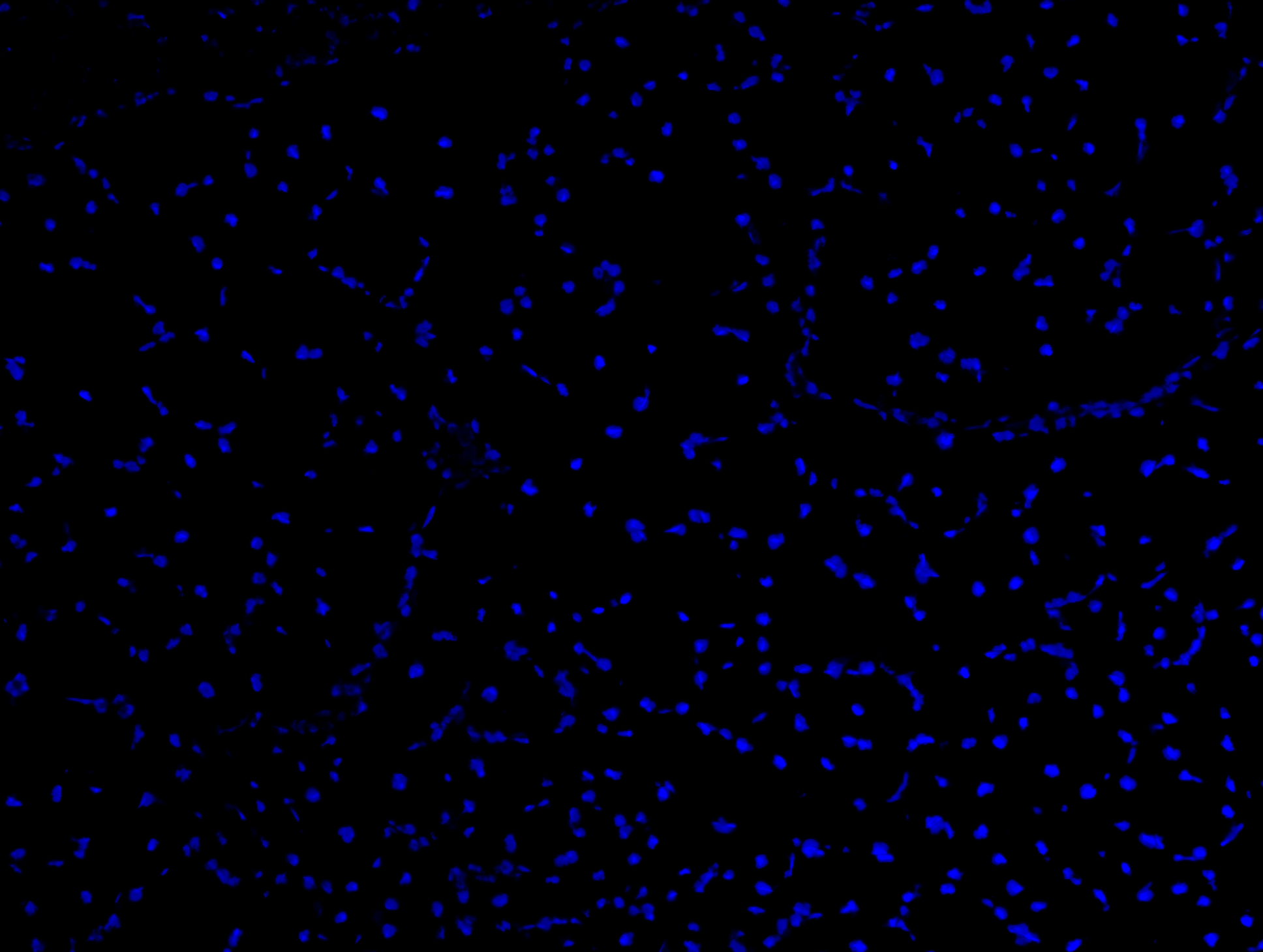

Supplement: Supplementary file 5 — Source Data Fig. 4 [file 44321_2024_49_MOESM5_ESM.zip › Figure 4/4D/EXOEAA-PMO DAPI.tif]

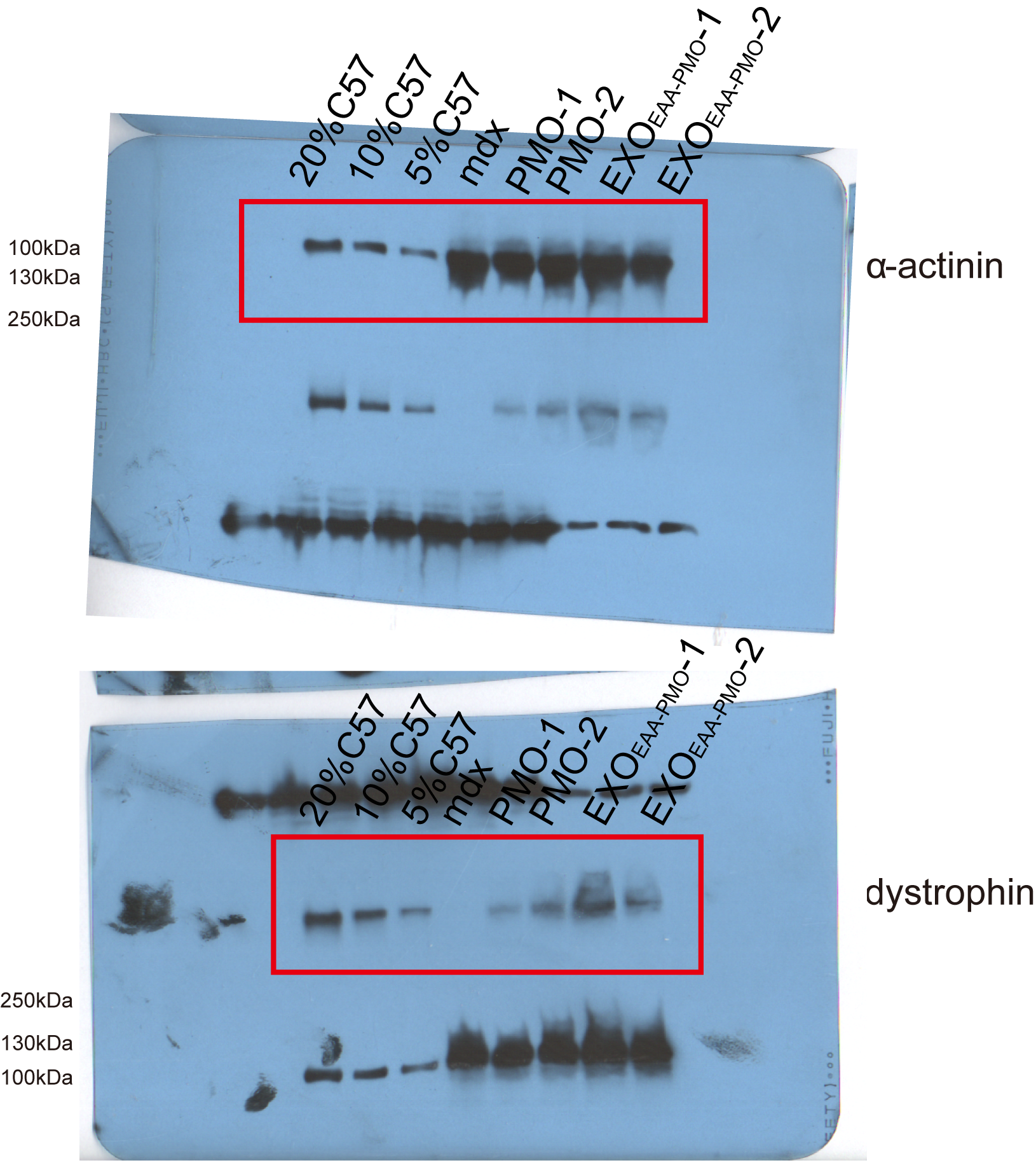

Supplement: Supplementary file 5 — Source Data Fig. 4 [file 44321_2024_49_MOESM5_ESM.zip › Figure 4/4F/4F.tif]

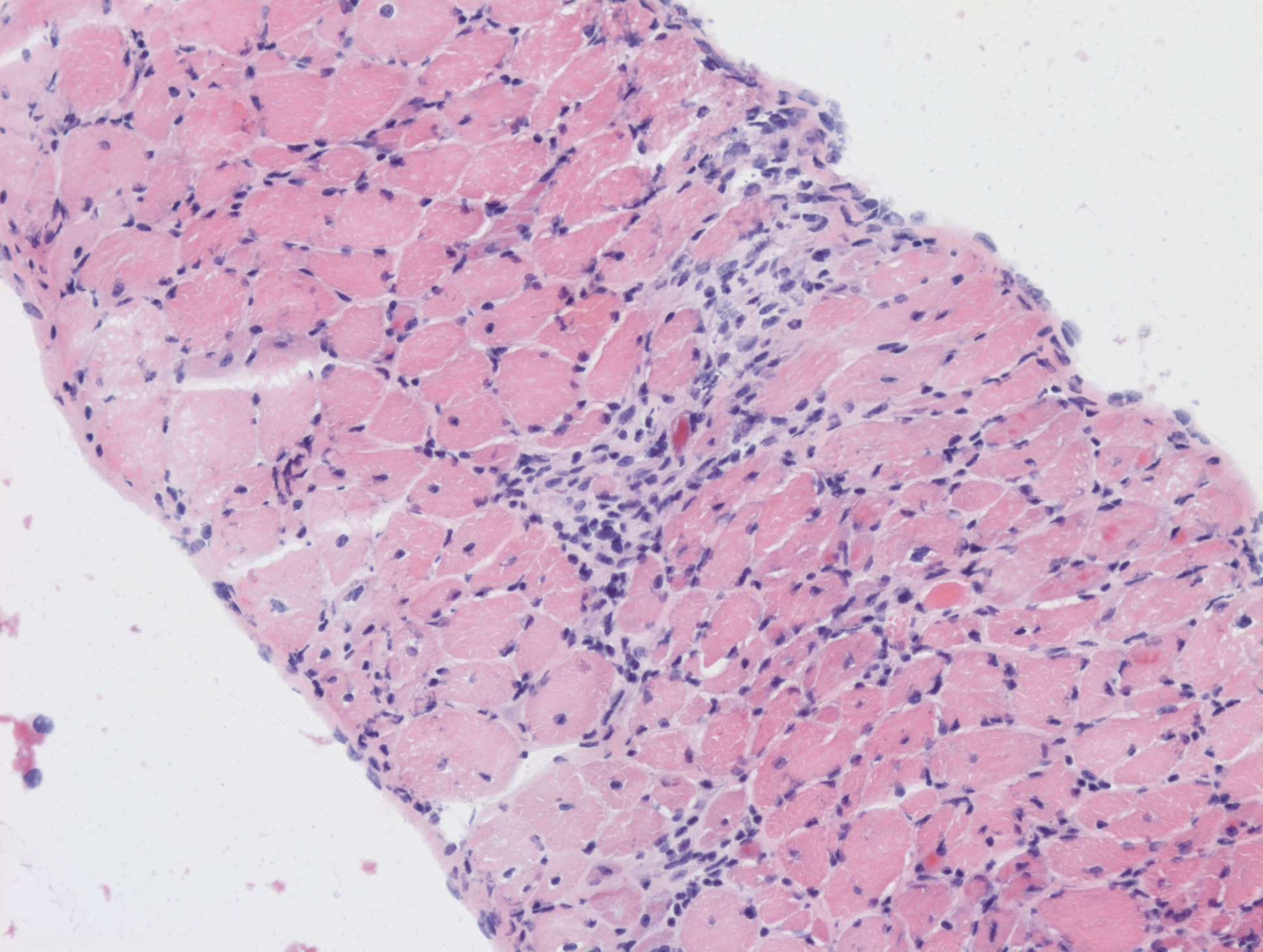

Supplement: Supplementary file 6 — Source Data Fig. 5 [file 44321_2024_49_MOESM6_ESM.zip › Figure 5/5G/PMO-D.tif]

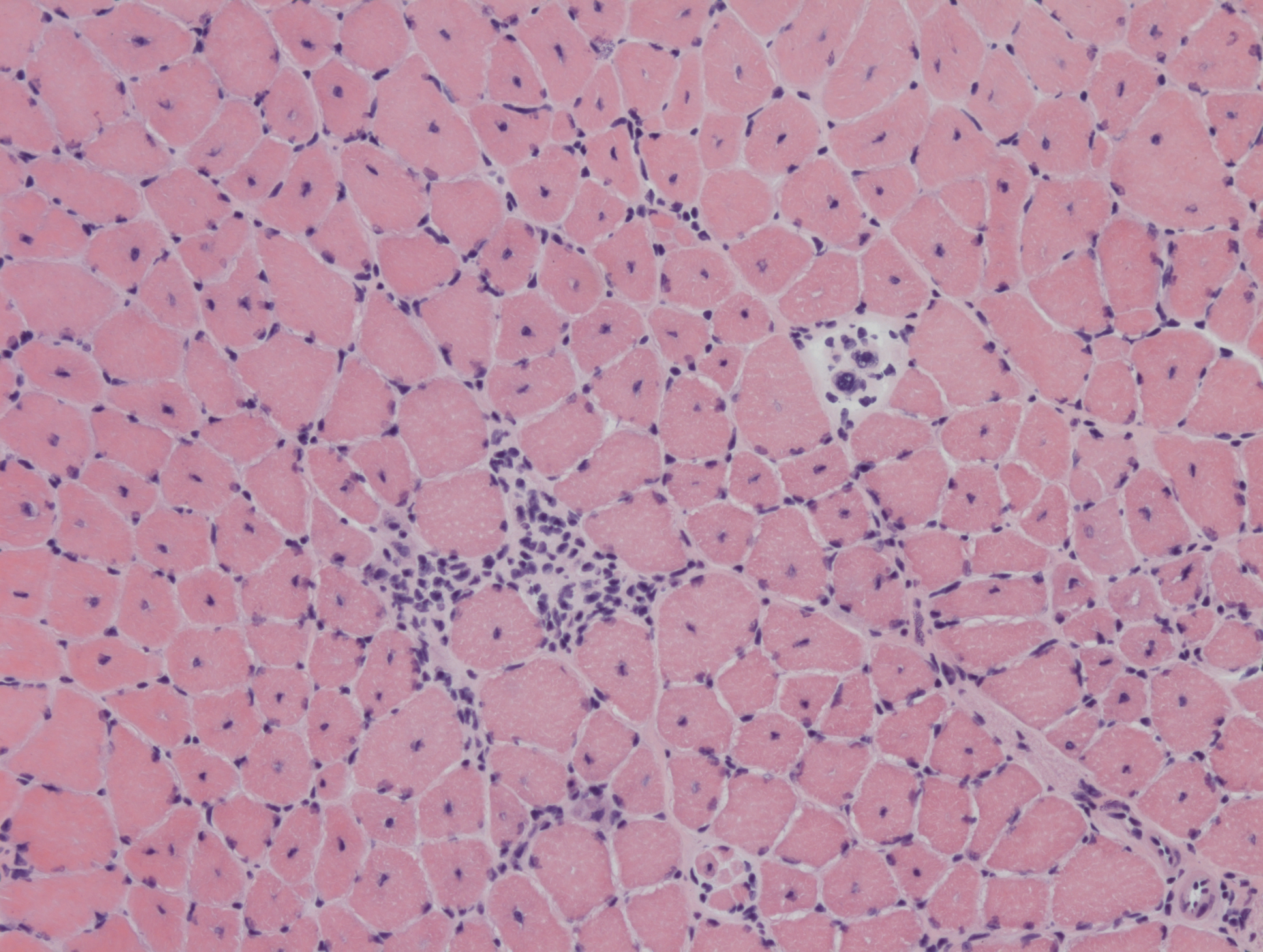

Supplement: Supplementary file 6 — Source Data Fig. 5 [file 44321_2024_49_MOESM6_ESM.zip › Figure 5/5G/PMO-G.tif]

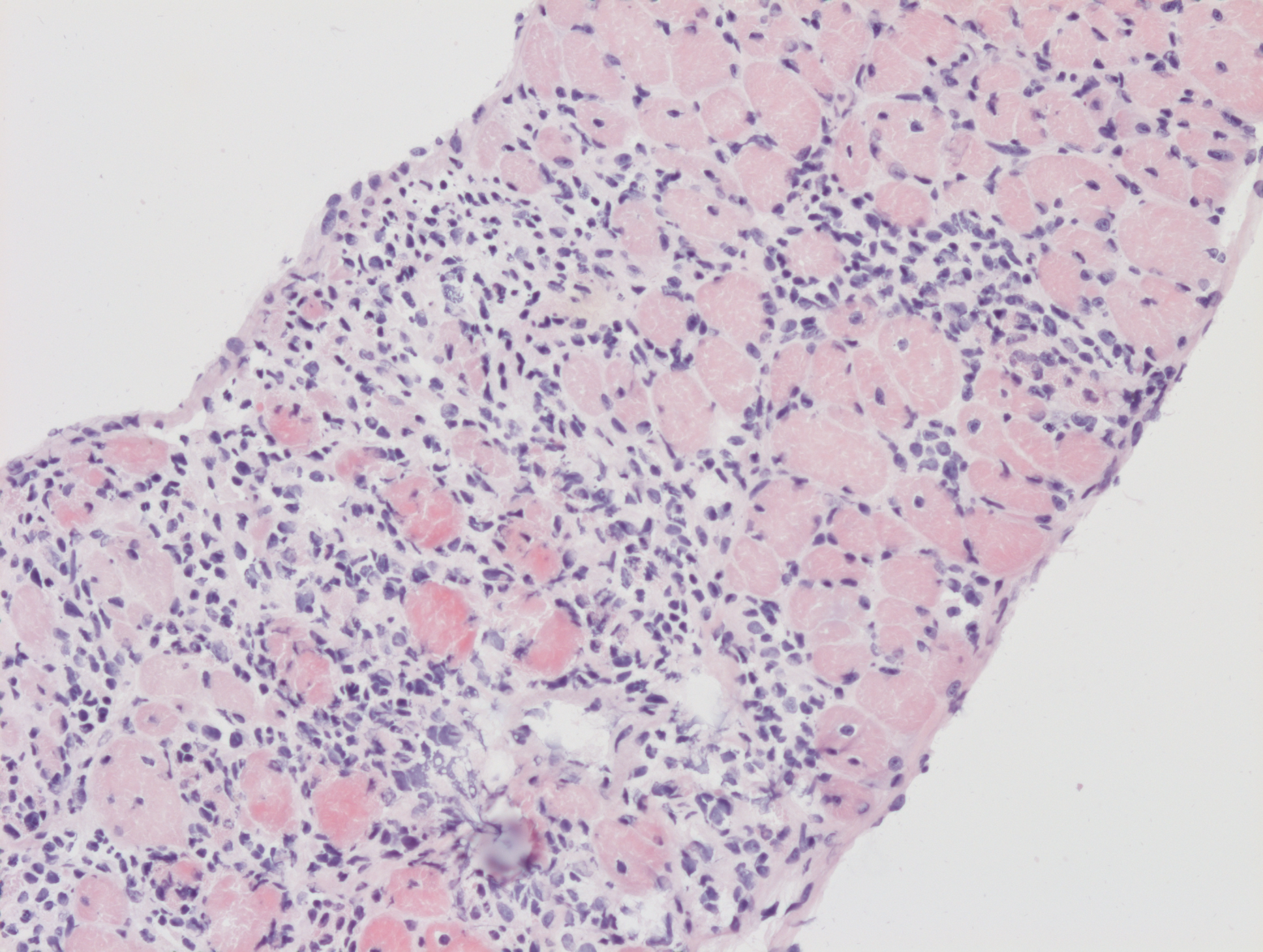

Supplement: Supplementary file 6 — Source Data Fig. 5 [file 44321_2024_49_MOESM6_ESM.zip › Figure 5/5G/mdx-D.tif]

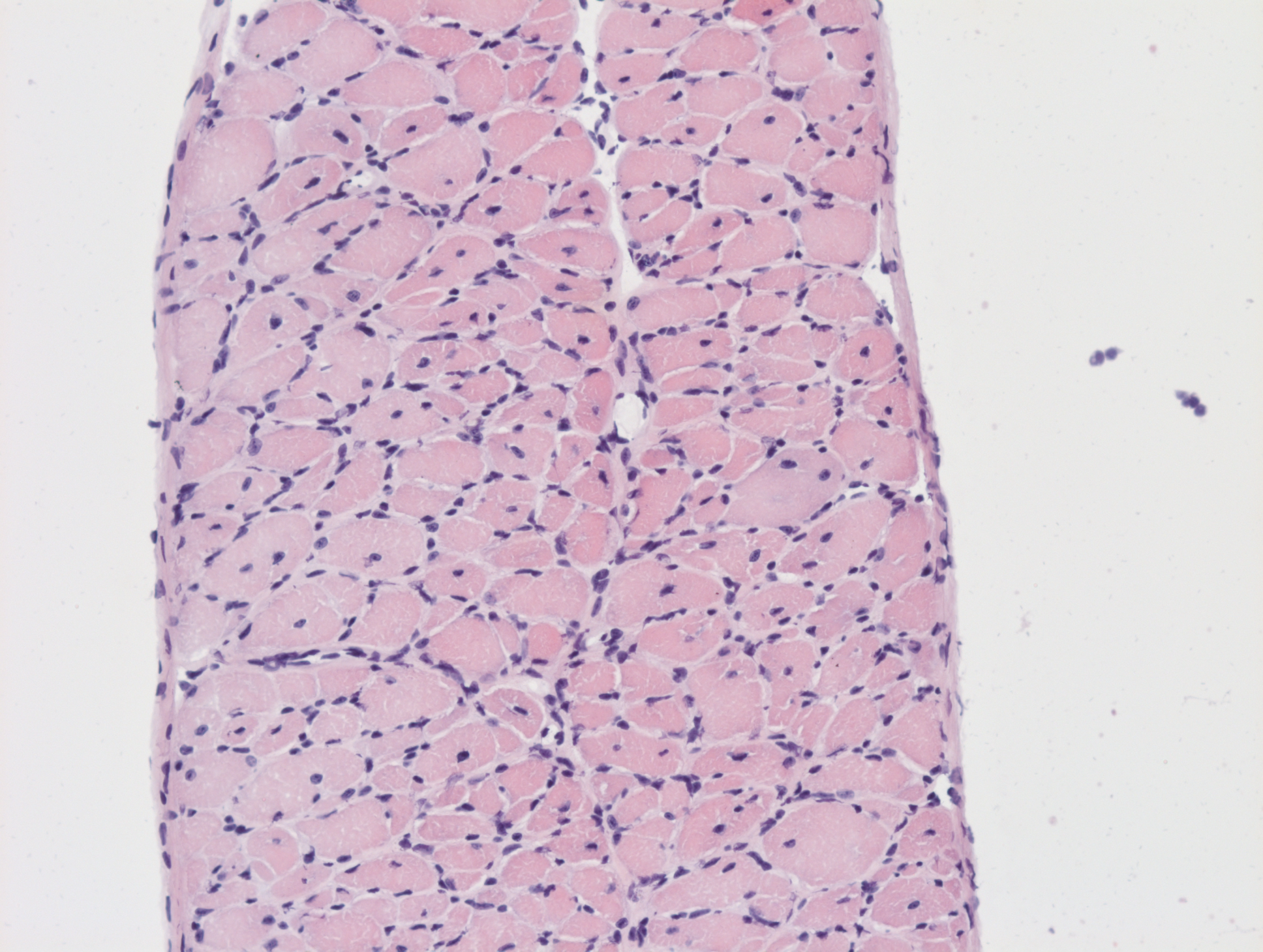

Supplement: Supplementary file 6 — Source Data Fig. 5 [file 44321_2024_49_MOESM6_ESM.zip › Figure 5/5G/EXOEAA-PMO-D.tif]

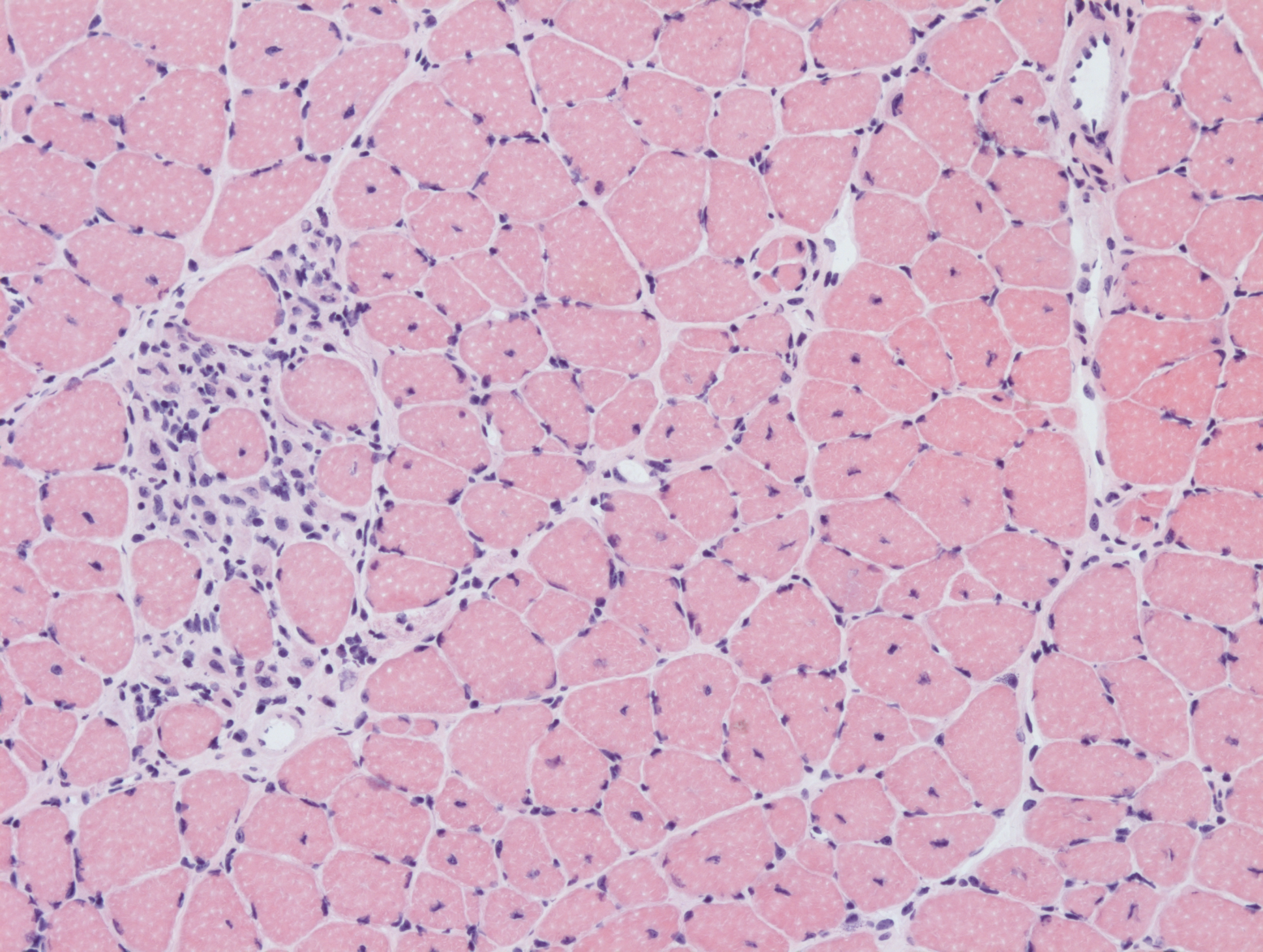

Supplement: Supplementary file 6 — Source Data Fig. 5 [file 44321_2024_49_MOESM6_ESM.zip › Figure 5/5G/mdx-G.tif]

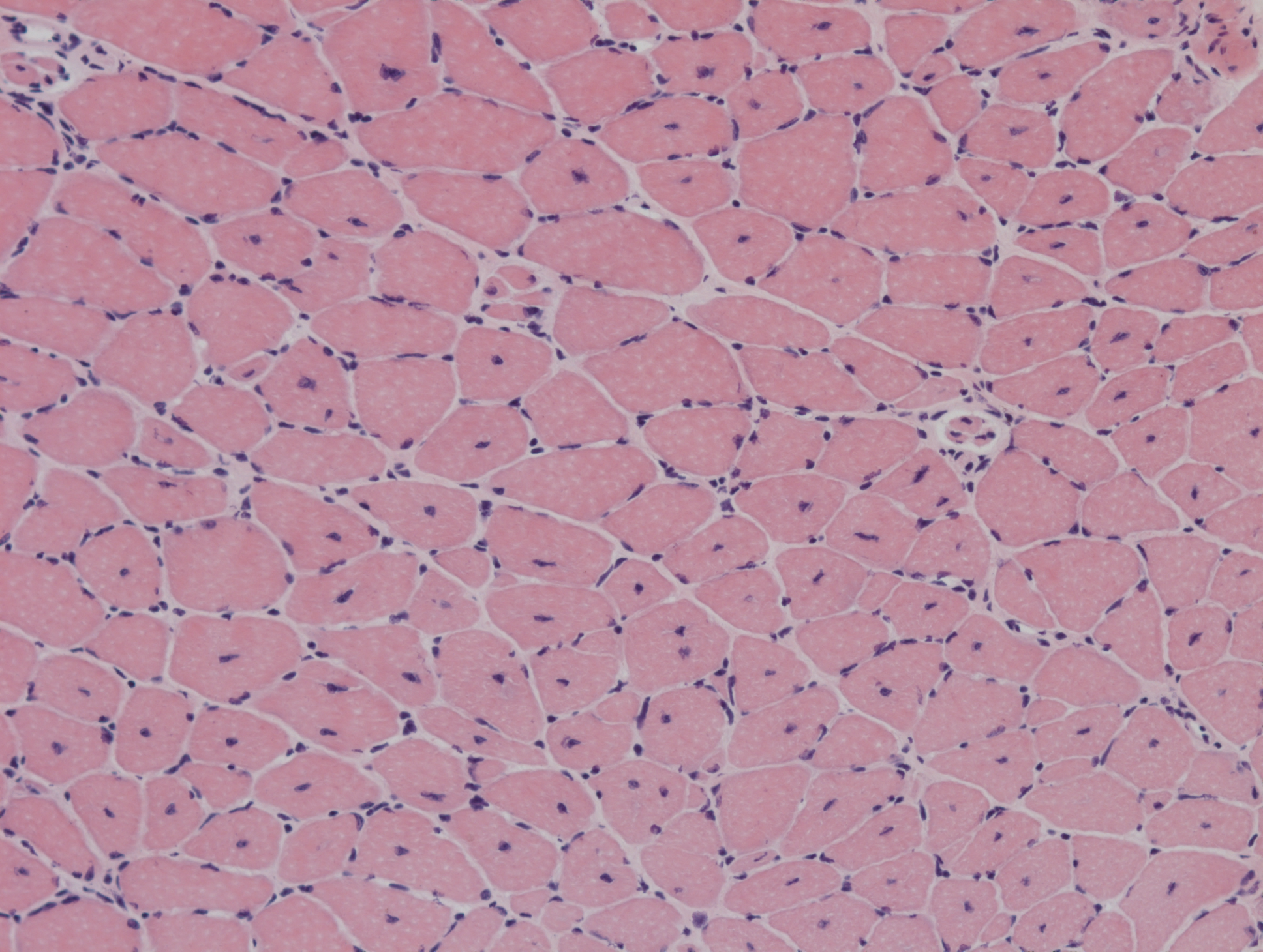

Supplement: Supplementary file 6 — Source Data Fig. 5 [file 44321_2024_49_MOESM6_ESM.zip › Figure 5/5G/EXOEAA-PMO-G.tif]

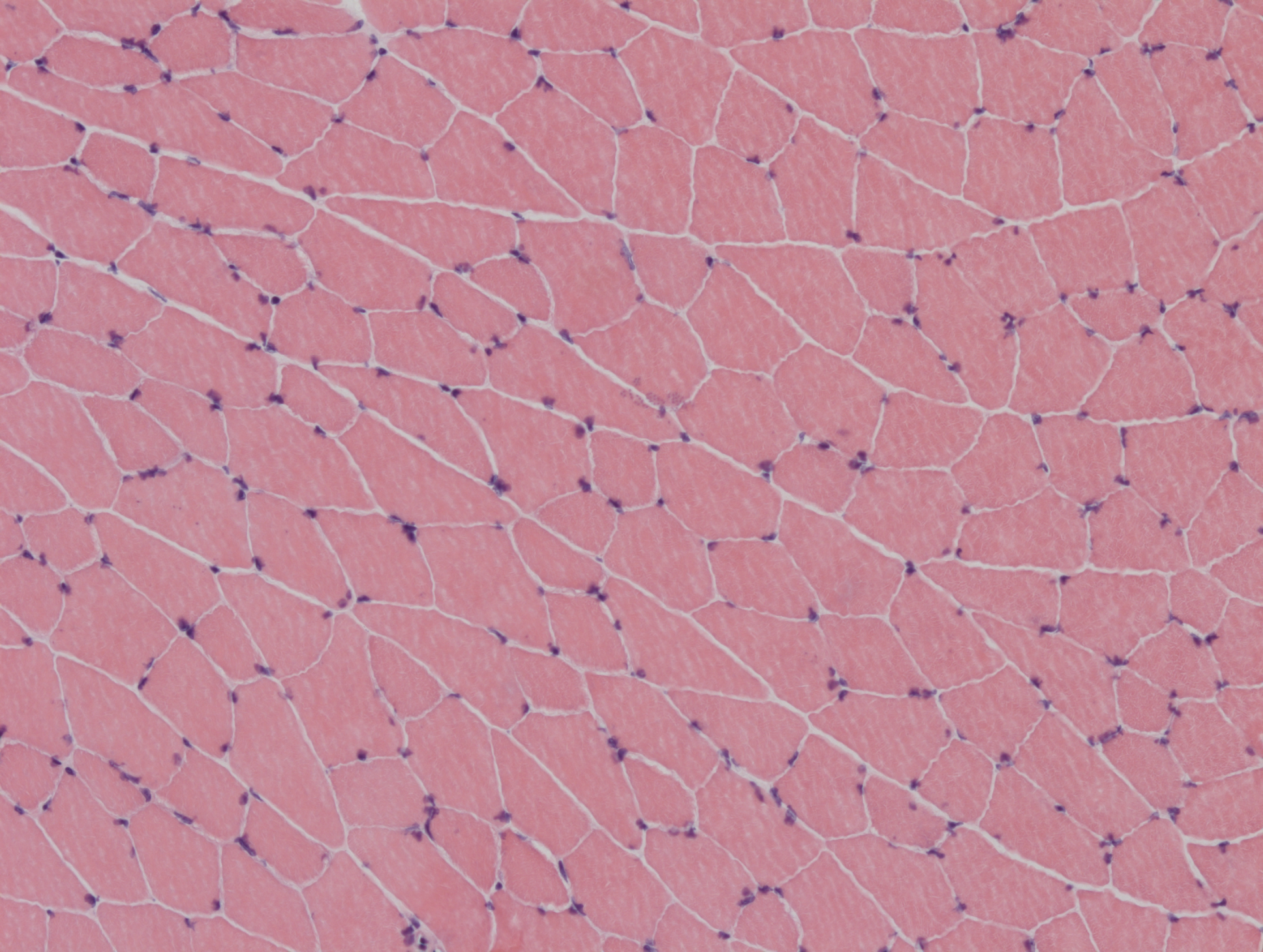

Supplement: Supplementary file 6 — Source Data Fig. 5 [file 44321_2024_49_MOESM6_ESM.zip › Figure 5/5G/C57-G.tif]

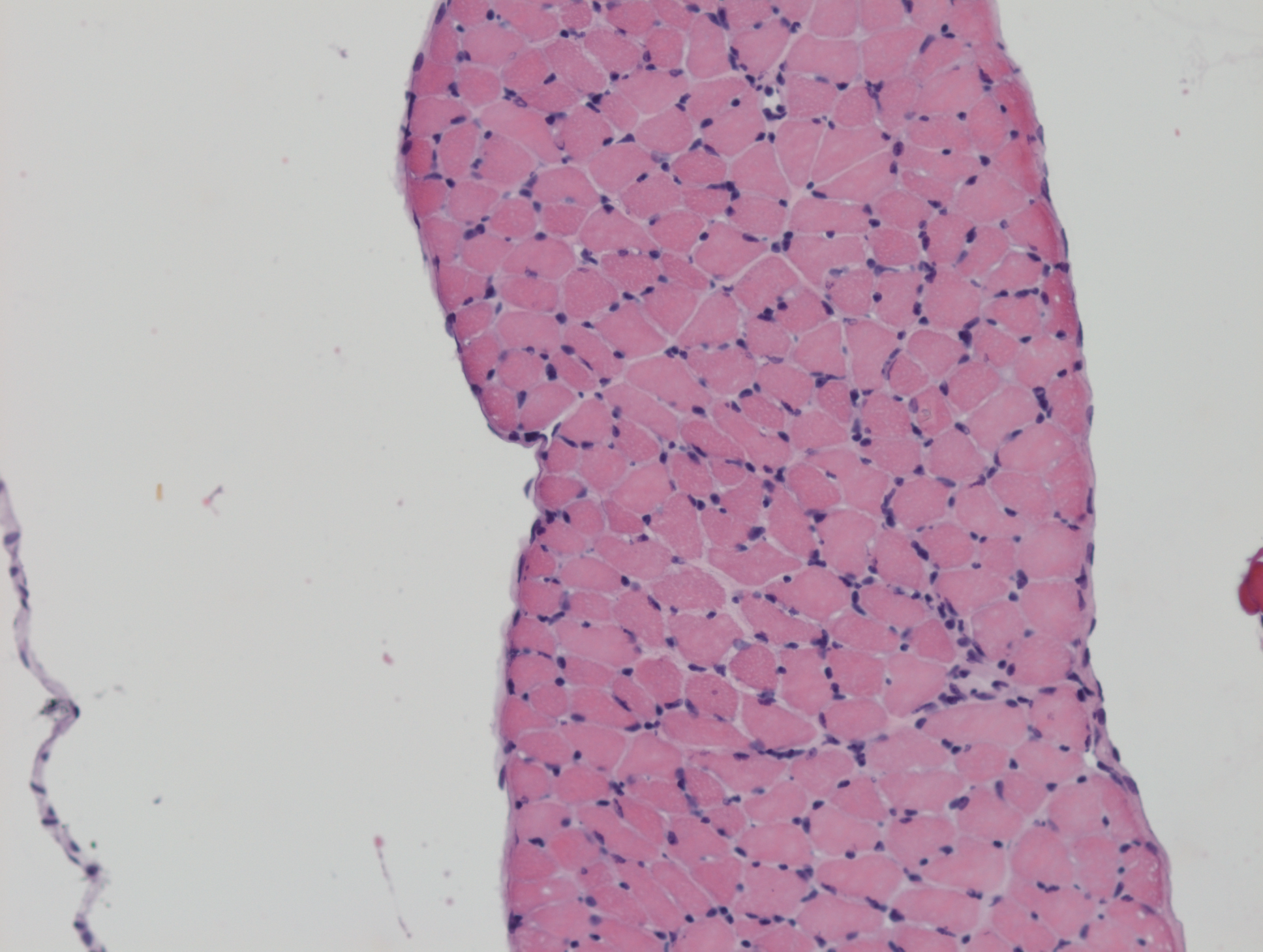

Supplement: Supplementary file 6 — Source Data Fig. 5 [file 44321_2024_49_MOESM6_ESM.zip › Figure 5/5G/C57-D.tif]

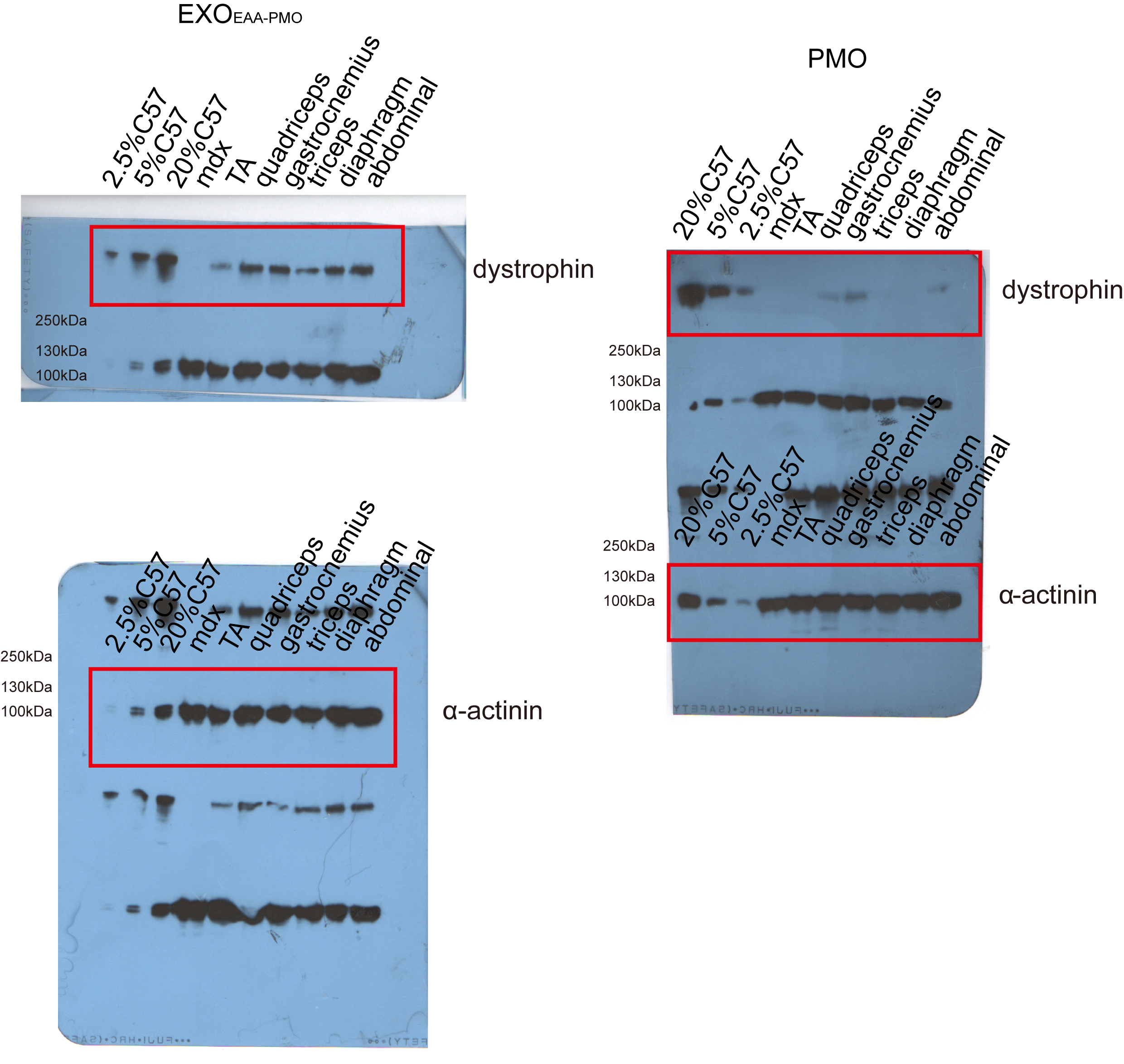

Supplement: Supplementary file 6 — Source Data Fig. 5 [file 44321_2024_49_MOESM6_ESM.zip › Figure 5/5B/5B.tif]

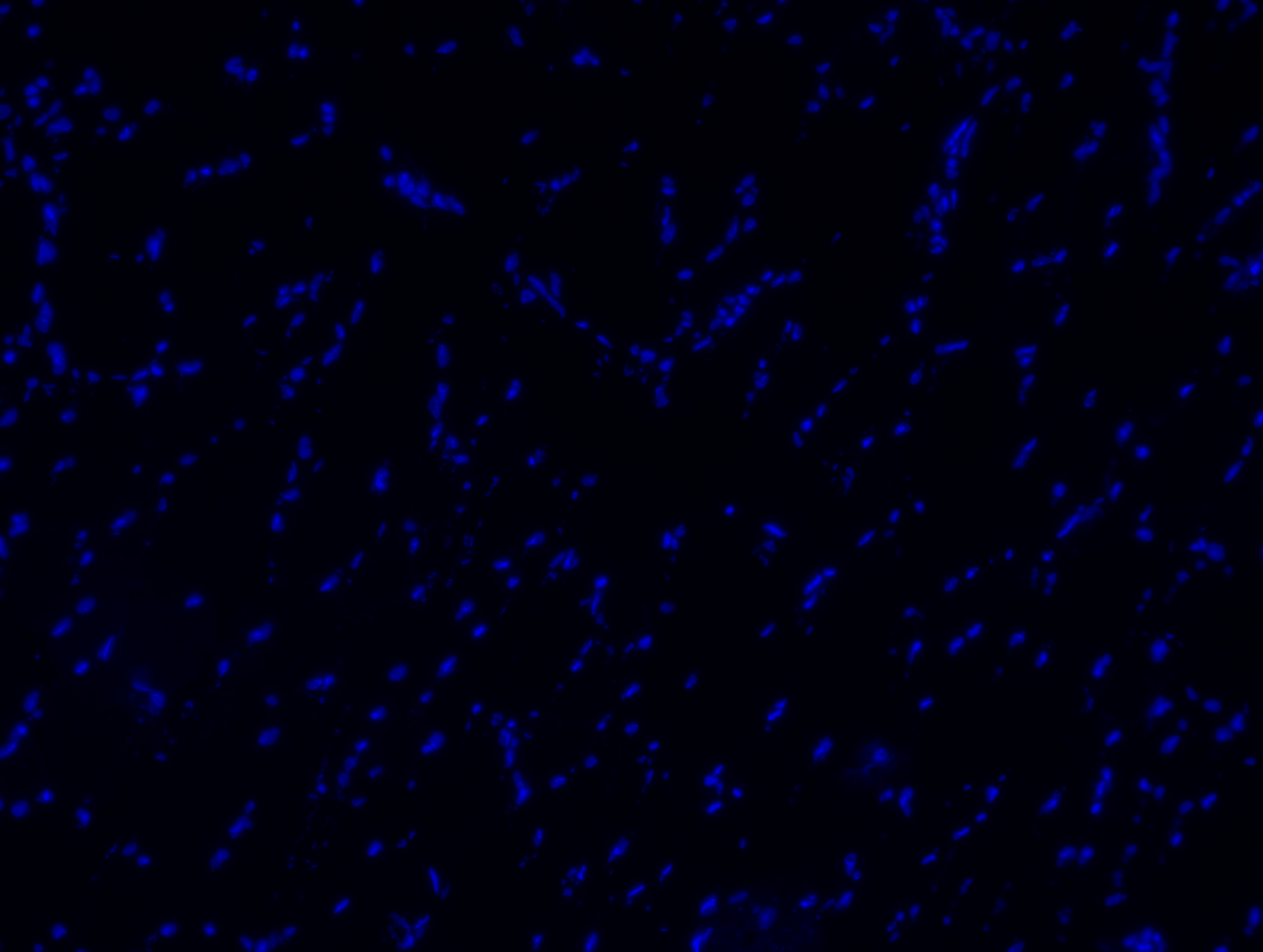

Supplement: Supplementary file 6 — Source Data Fig. 5 [file 44321_2024_49_MOESM6_ESM.zip › Figure 5/5A/mdx/T DAPI.tif]

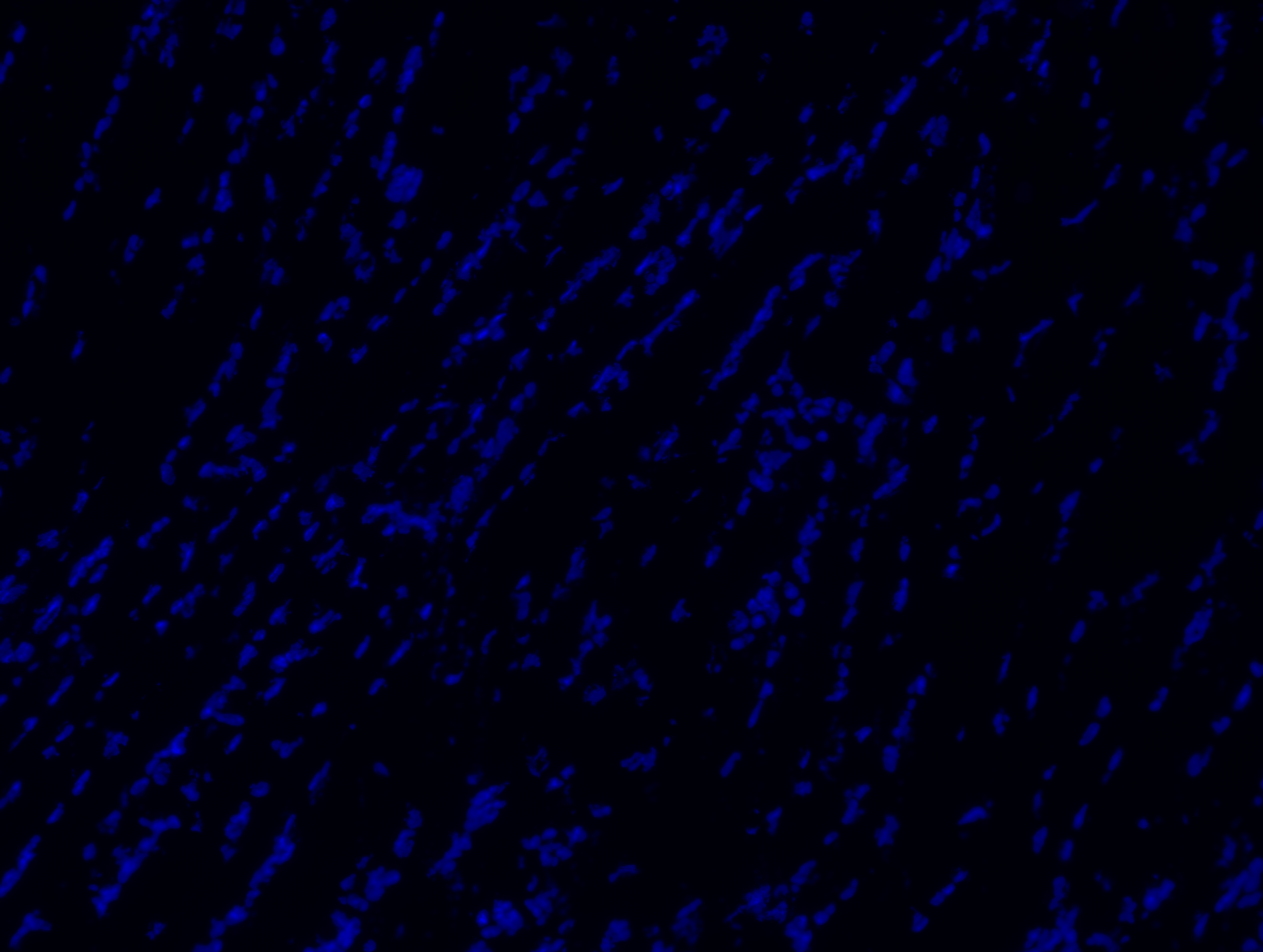

Supplement: Supplementary file 6 — Source Data Fig. 5 [file 44321_2024_49_MOESM6_ESM.zip › Figure 5/5A/mdx/A DAPI.tif]

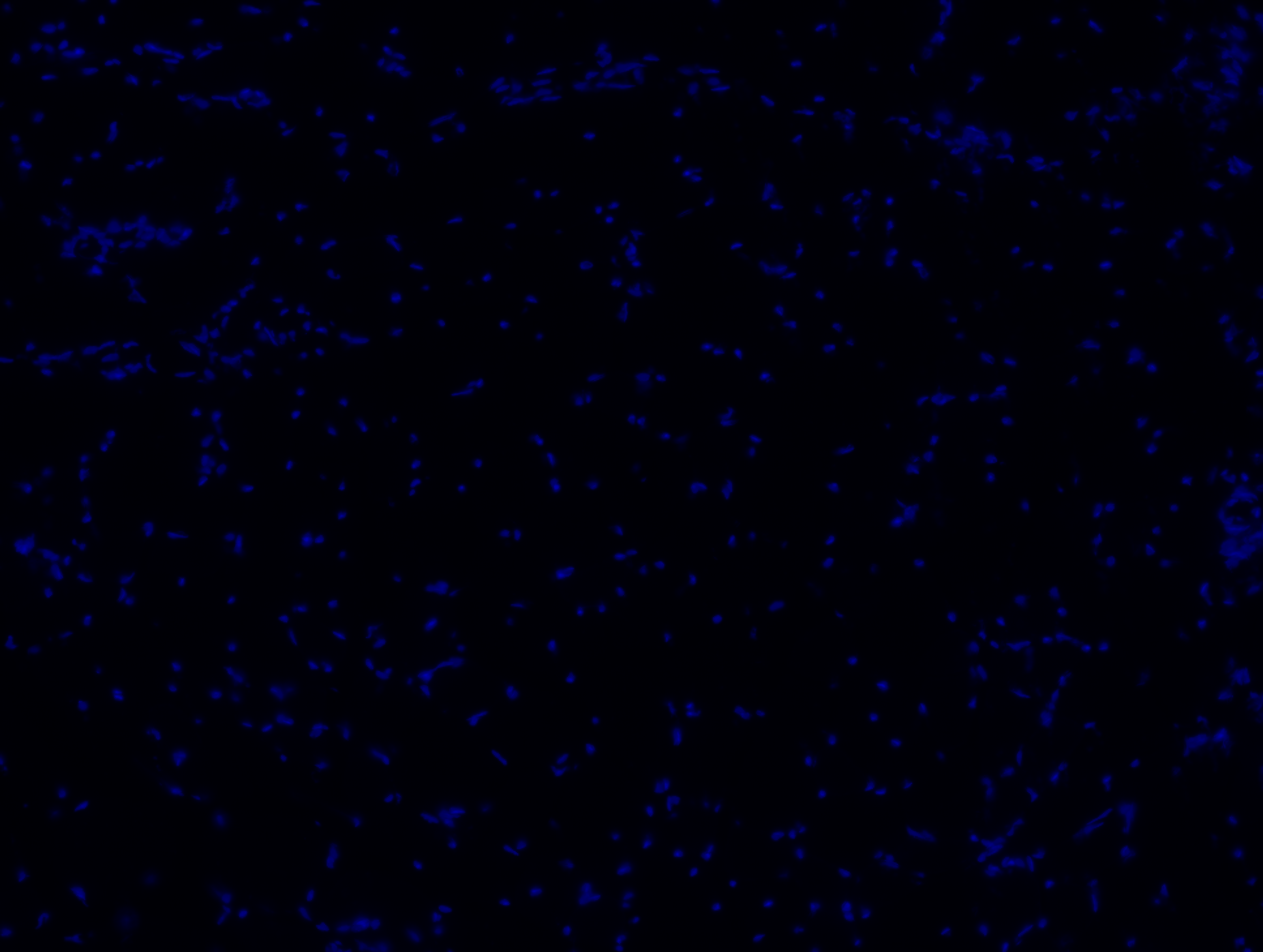

Supplement: Supplementary file 6 — Source Data Fig. 5 [file 44321_2024_49_MOESM6_ESM.zip › Figure 5/5A/mdx/G DAPI.tif]

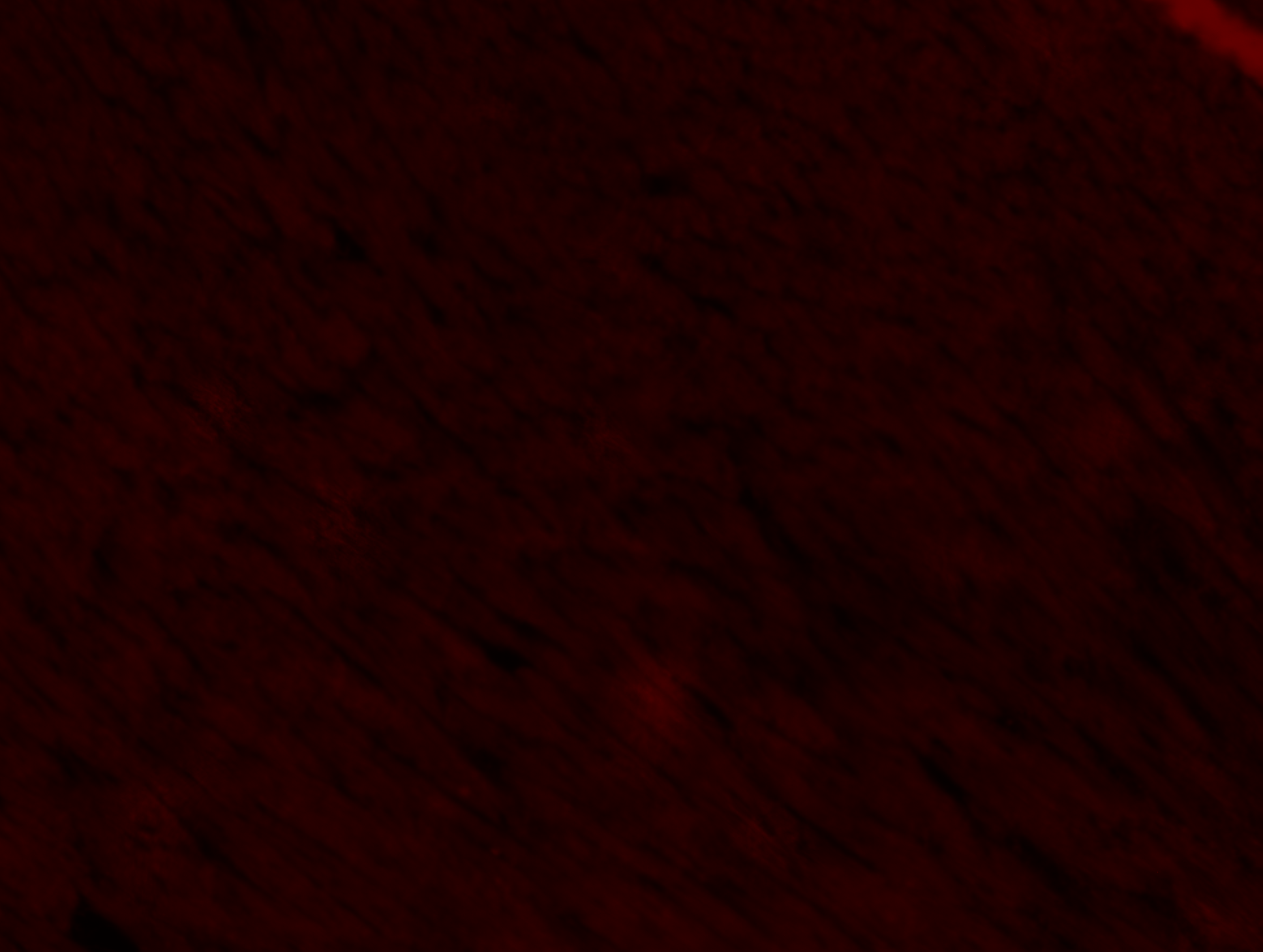

Supplement: Supplementary file 6 — Source Data Fig. 5 [file 44321_2024_49_MOESM6_ESM.zip › Figure 5/5A/mdx/H DYS.tif]

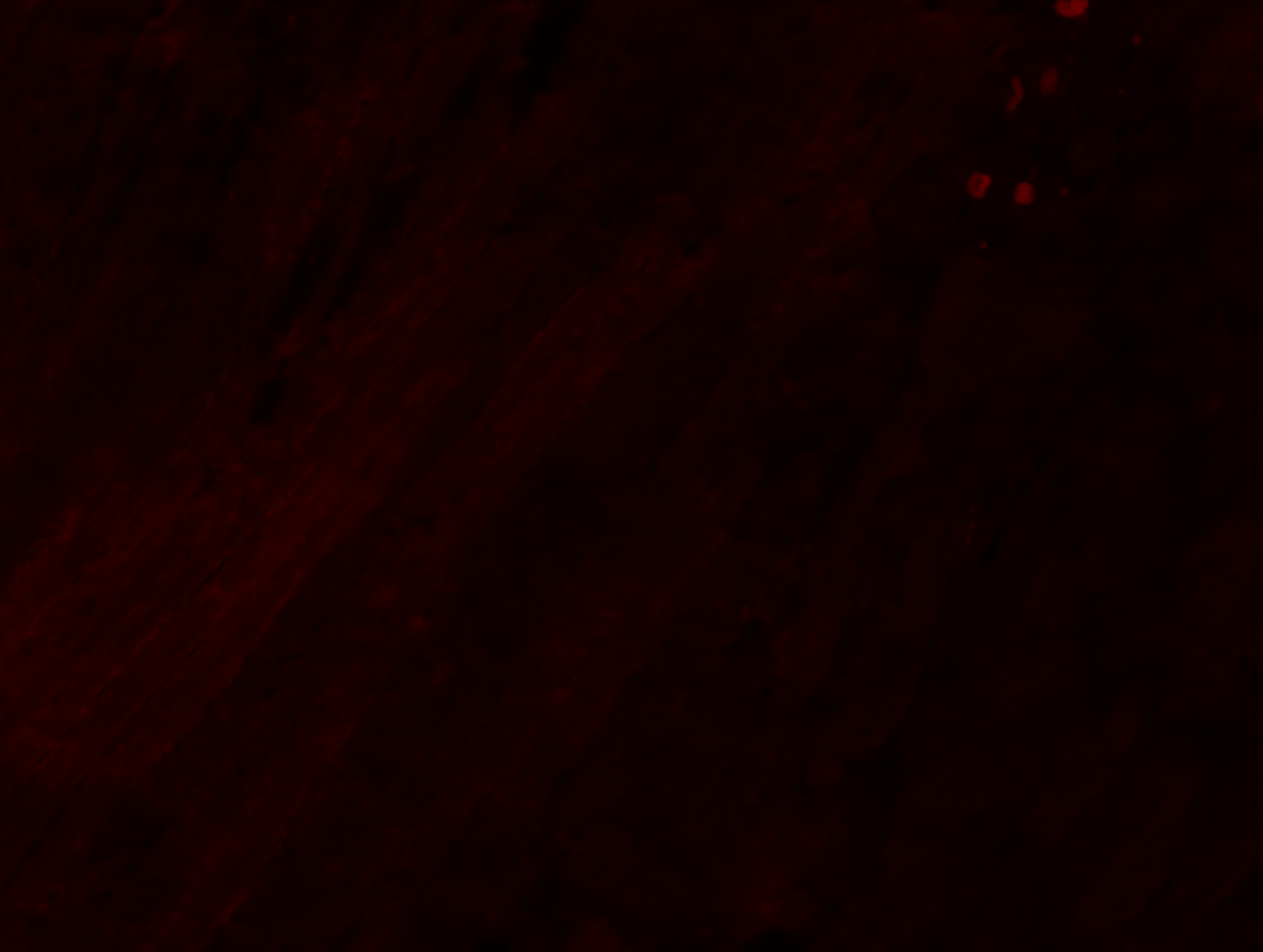

Supplement: Supplementary file 6 — Source Data Fig. 5 [file 44321_2024_49_MOESM6_ESM.zip › Figure 5/5A/mdx/A DYS.tif]

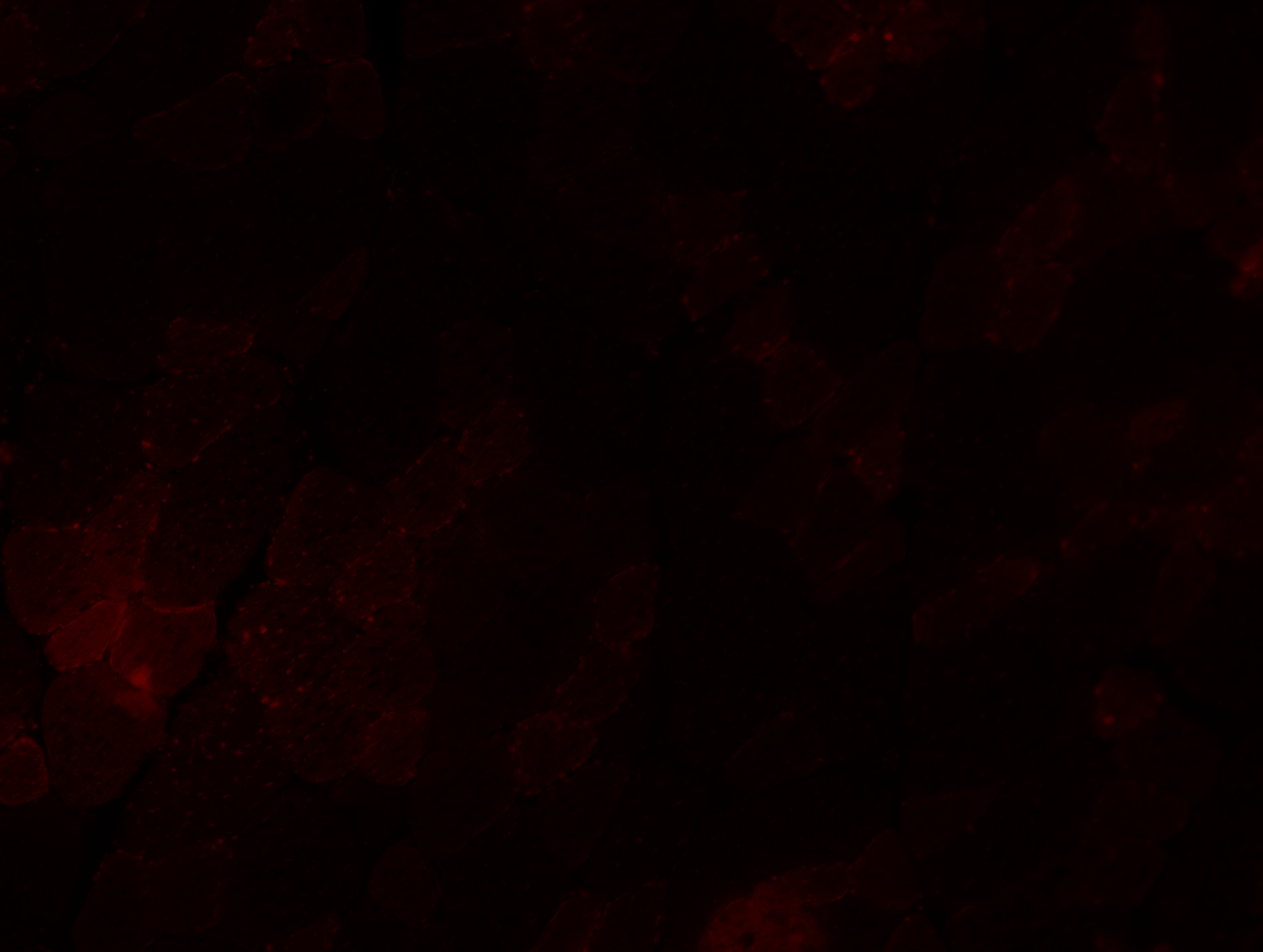

Supplement: Supplementary file 6 — Source Data Fig. 5 [file 44321_2024_49_MOESM6_ESM.zip › Figure 5/5A/mdx/T DYS.tif]

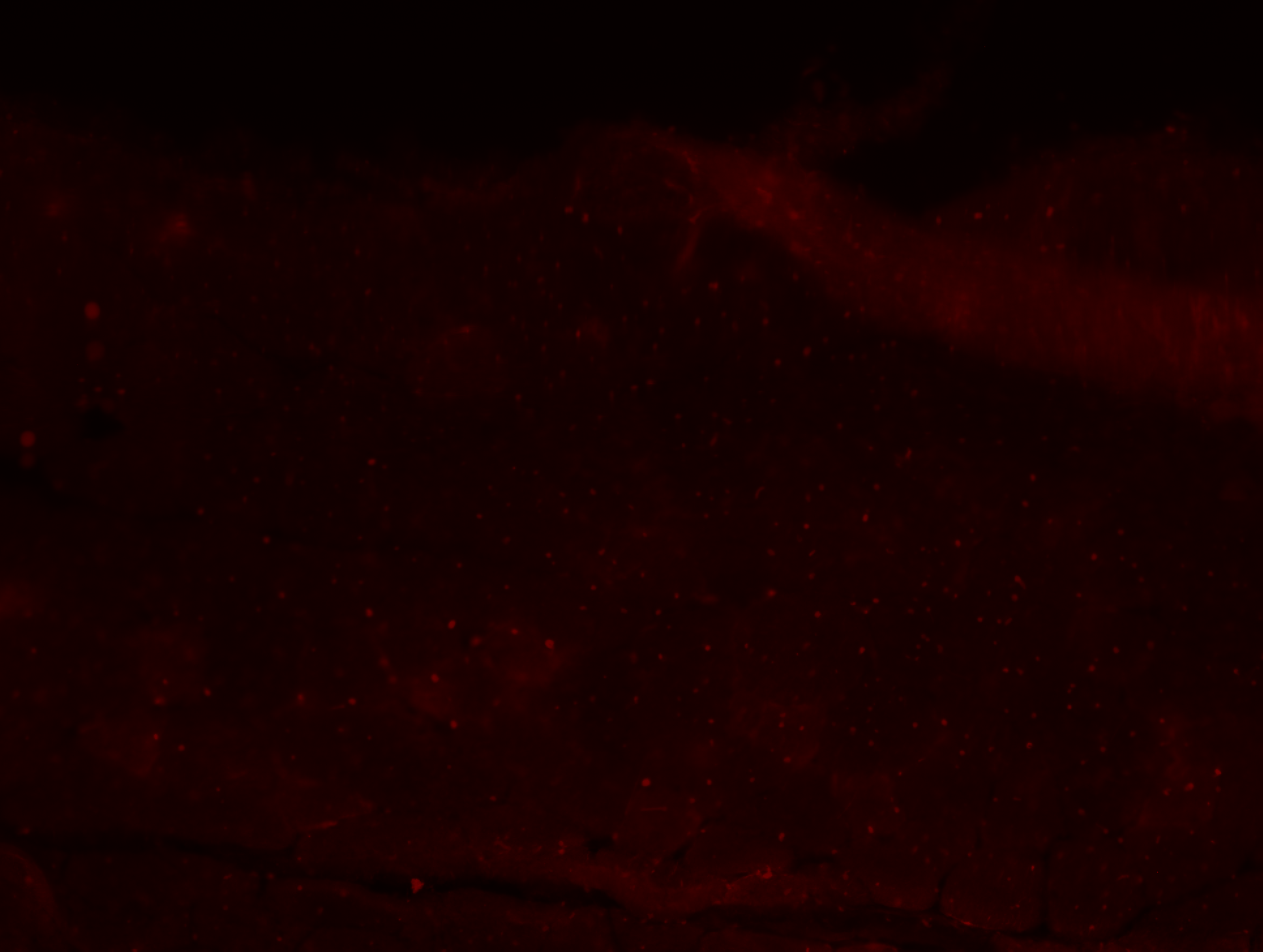

Supplement: Supplementary file 6 — Source Data Fig. 5 [file 44321_2024_49_MOESM6_ESM.zip › Figure 5/5A/mdx/D DYS.tif]

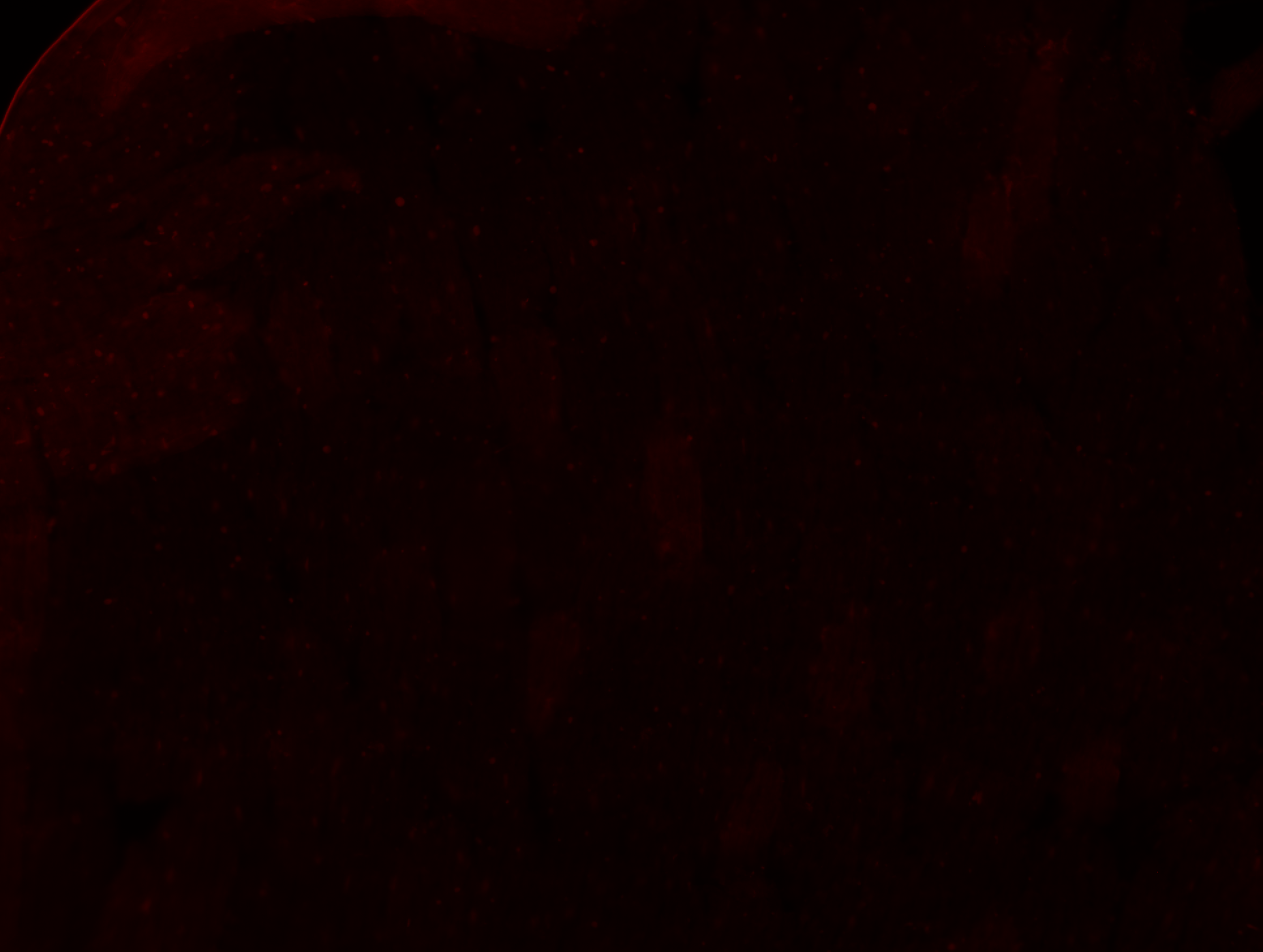

Supplement: Supplementary file 6 — Source Data Fig. 5 [file 44321_2024_49_MOESM6_ESM.zip › Figure 5/5A/mdx/Q DYS.tif]

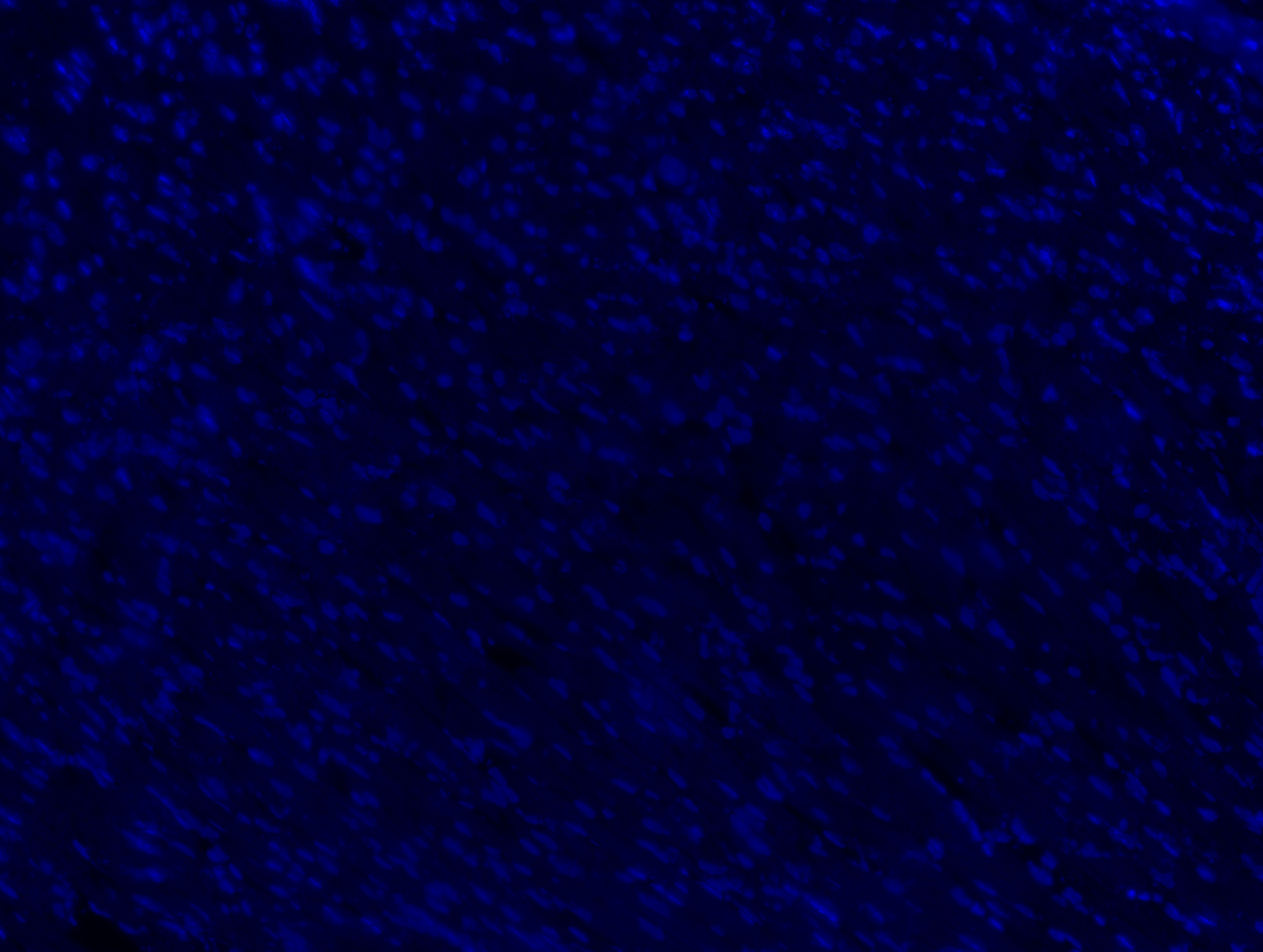

Supplement: Supplementary file 6 — Source Data Fig. 5 [file 44321_2024_49_MOESM6_ESM.zip › Figure 5/5A/mdx/H DAPI.tif]

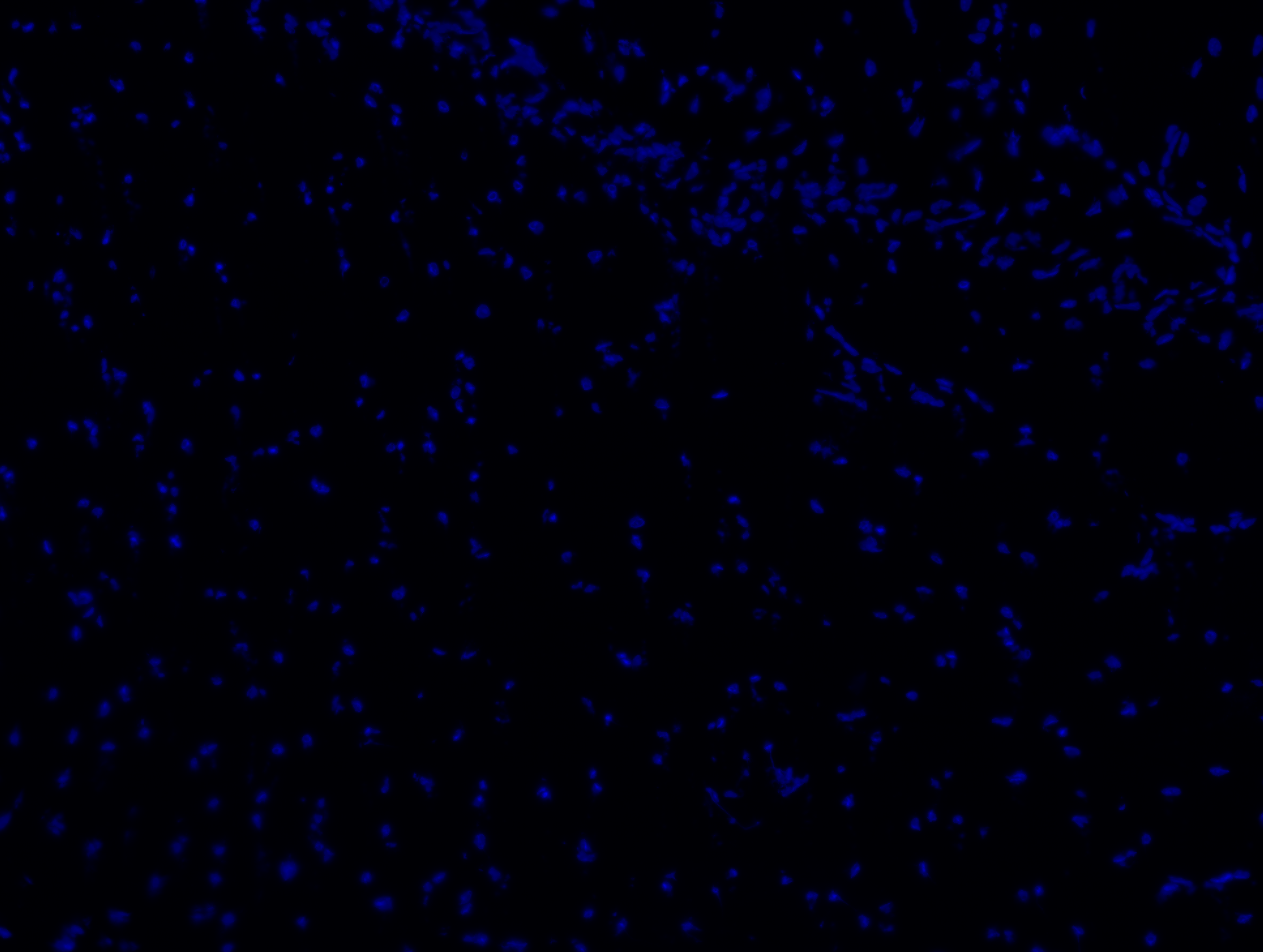

Supplement: Supplementary file 6 — Source Data Fig. 5 [file 44321_2024_49_MOESM6_ESM.zip › Figure 5/5A/mdx/TA DAPI.tif]

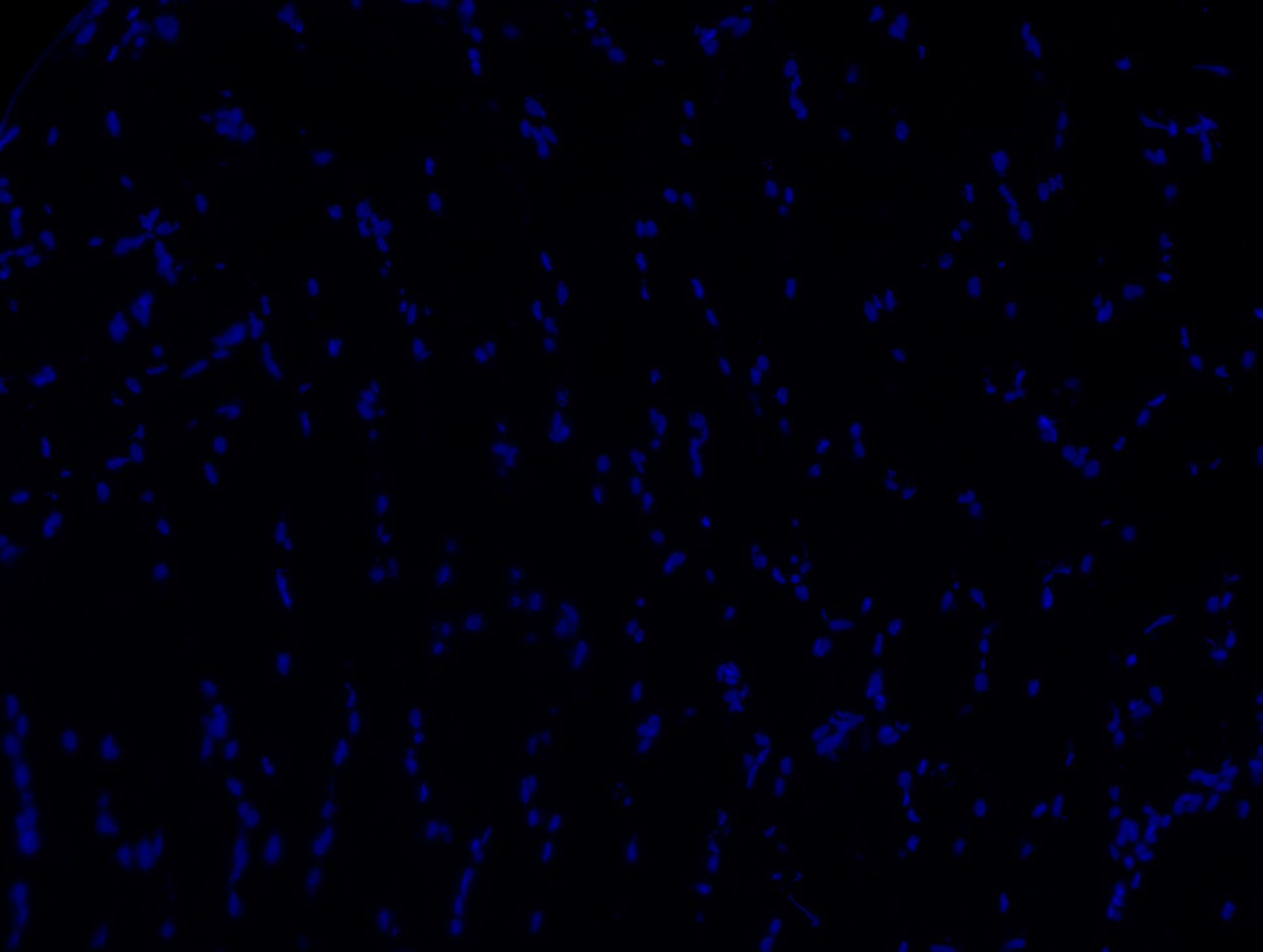

Supplement: Supplementary file 6 — Source Data Fig. 5 [file 44321_2024_49_MOESM6_ESM.zip › Figure 5/5A/mdx/Q DAPI.tif]

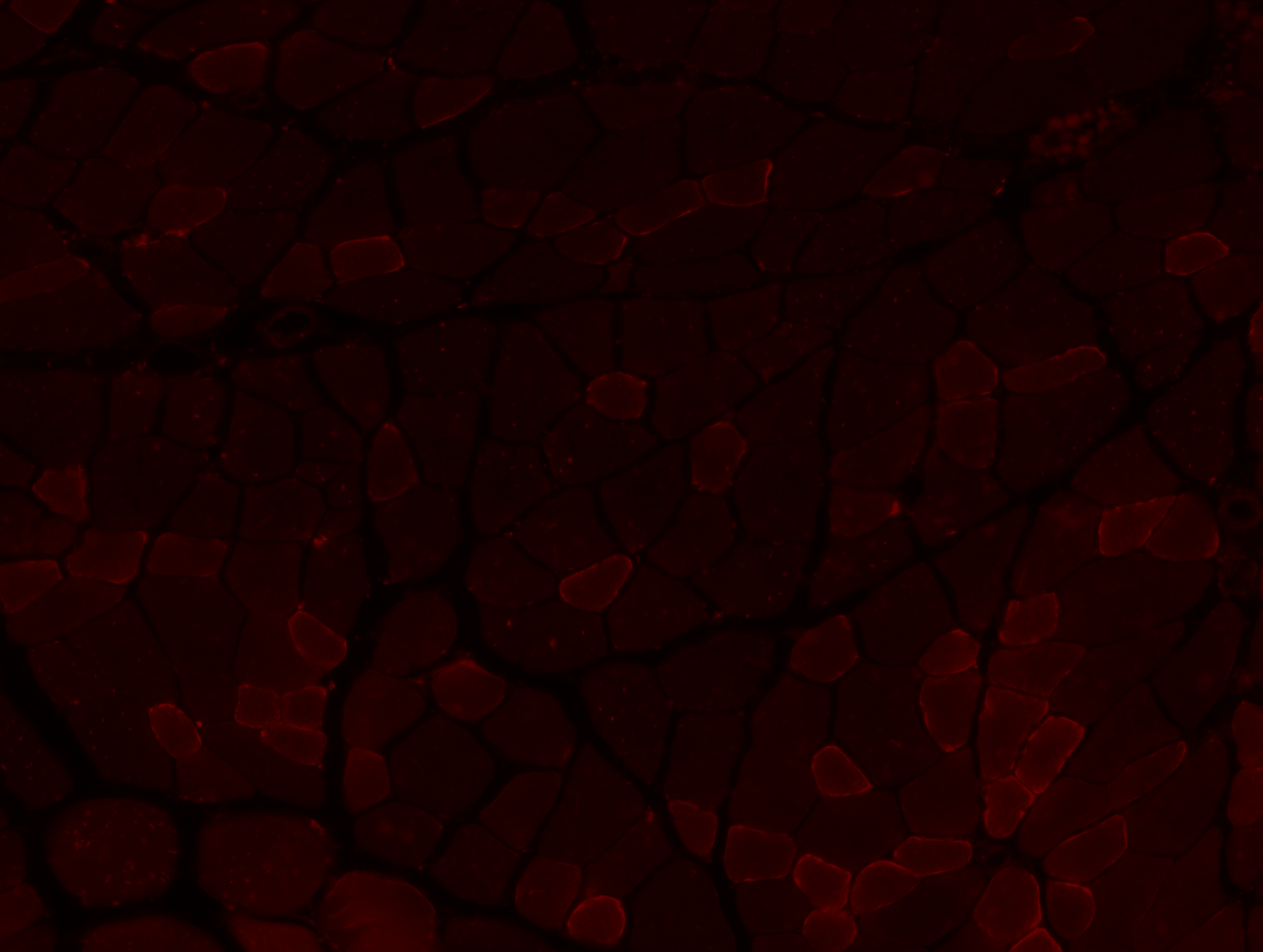

Supplement: Supplementary file 6 — Source Data Fig. 5 [file 44321_2024_49_MOESM6_ESM.zip › Figure 5/5A/mdx/G DYS.tif]

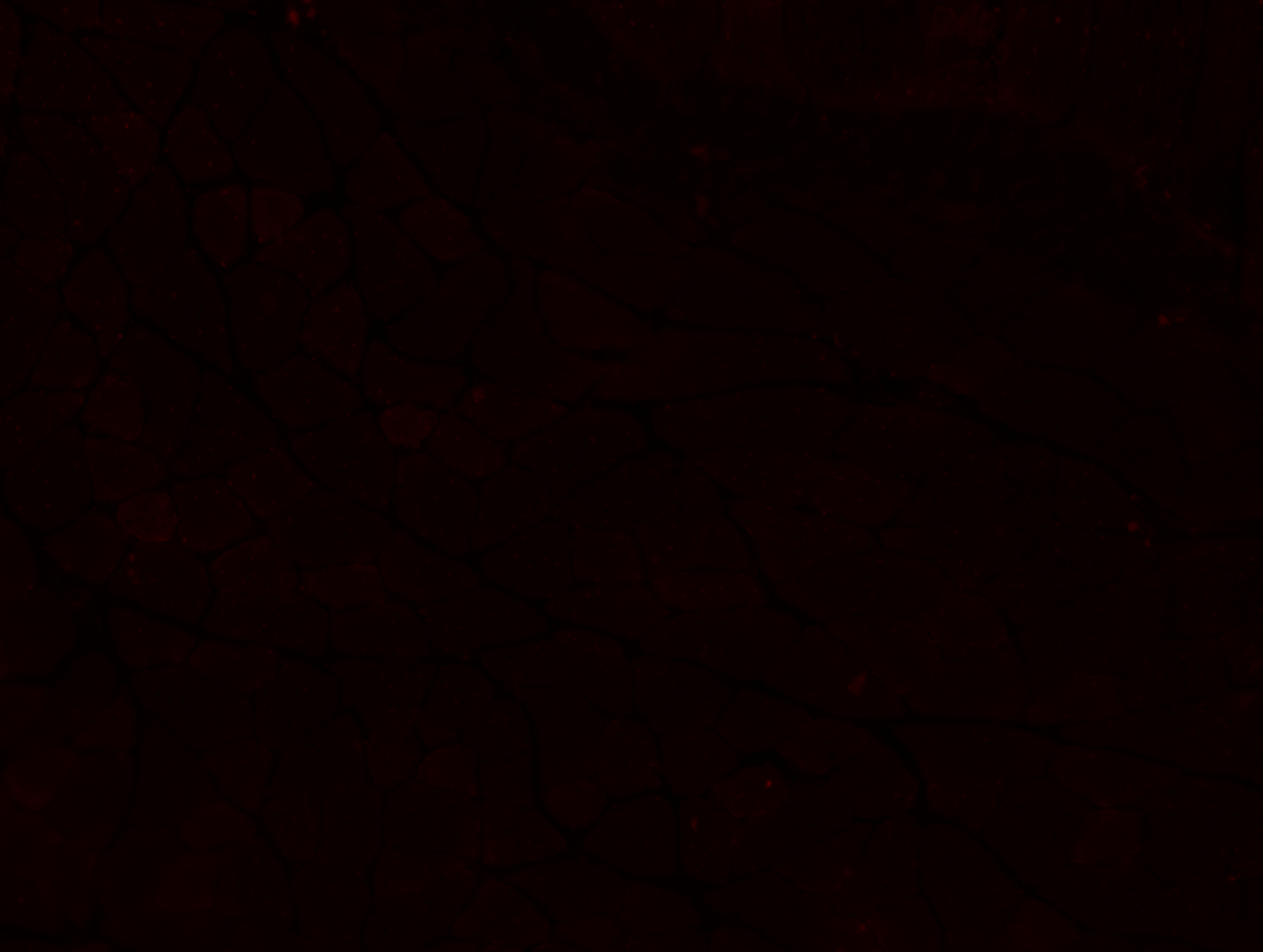

Supplement: Supplementary file 6 — Source Data Fig. 5 [file 44321_2024_49_MOESM6_ESM.zip › Figure 5/5A/mdx/TA DYS.tif]

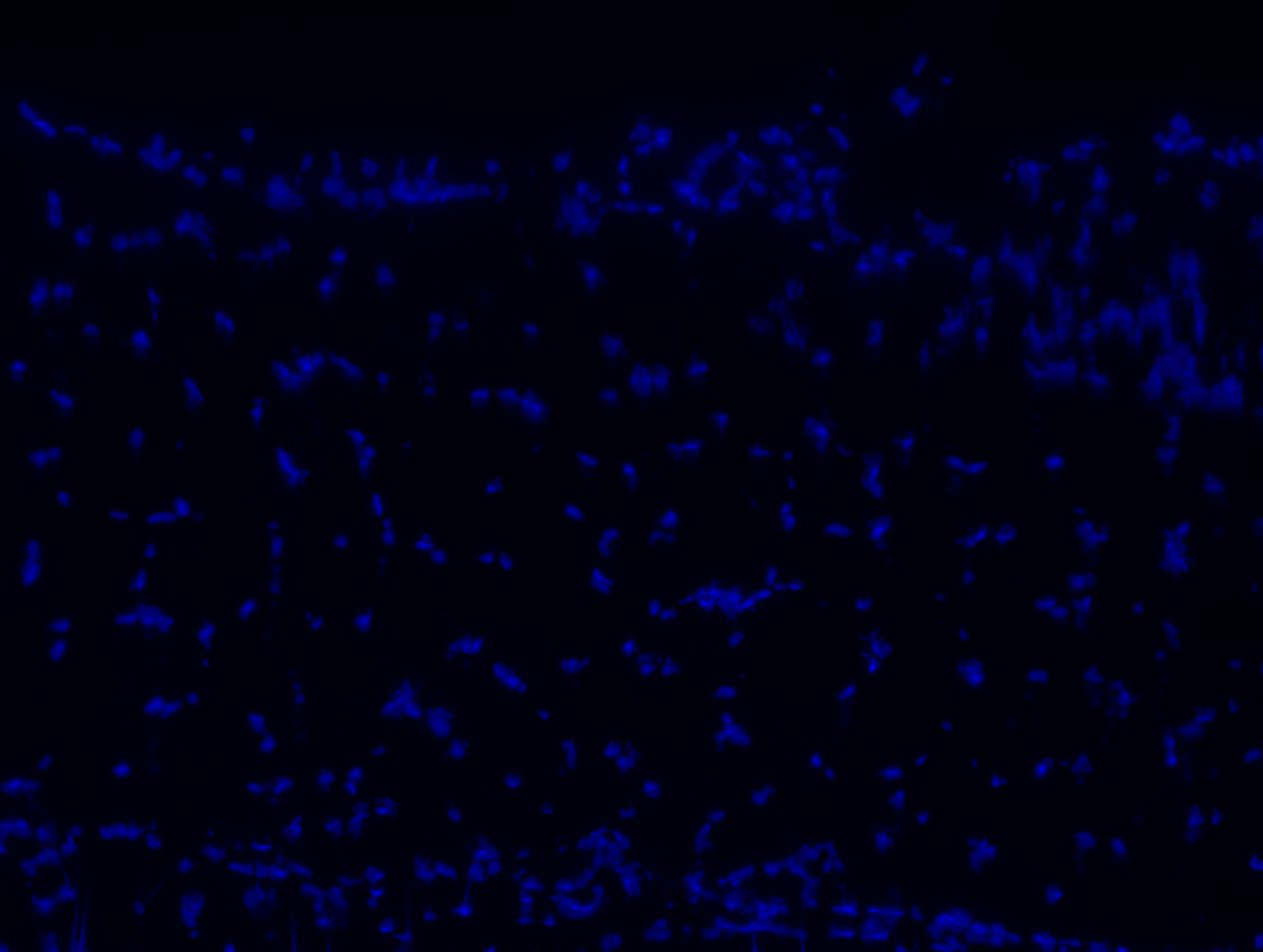

Supplement: Supplementary file 6 — Source Data Fig. 5 [file 44321_2024_49_MOESM6_ESM.zip › Figure 5/5A/mdx/D DAPI.tif]

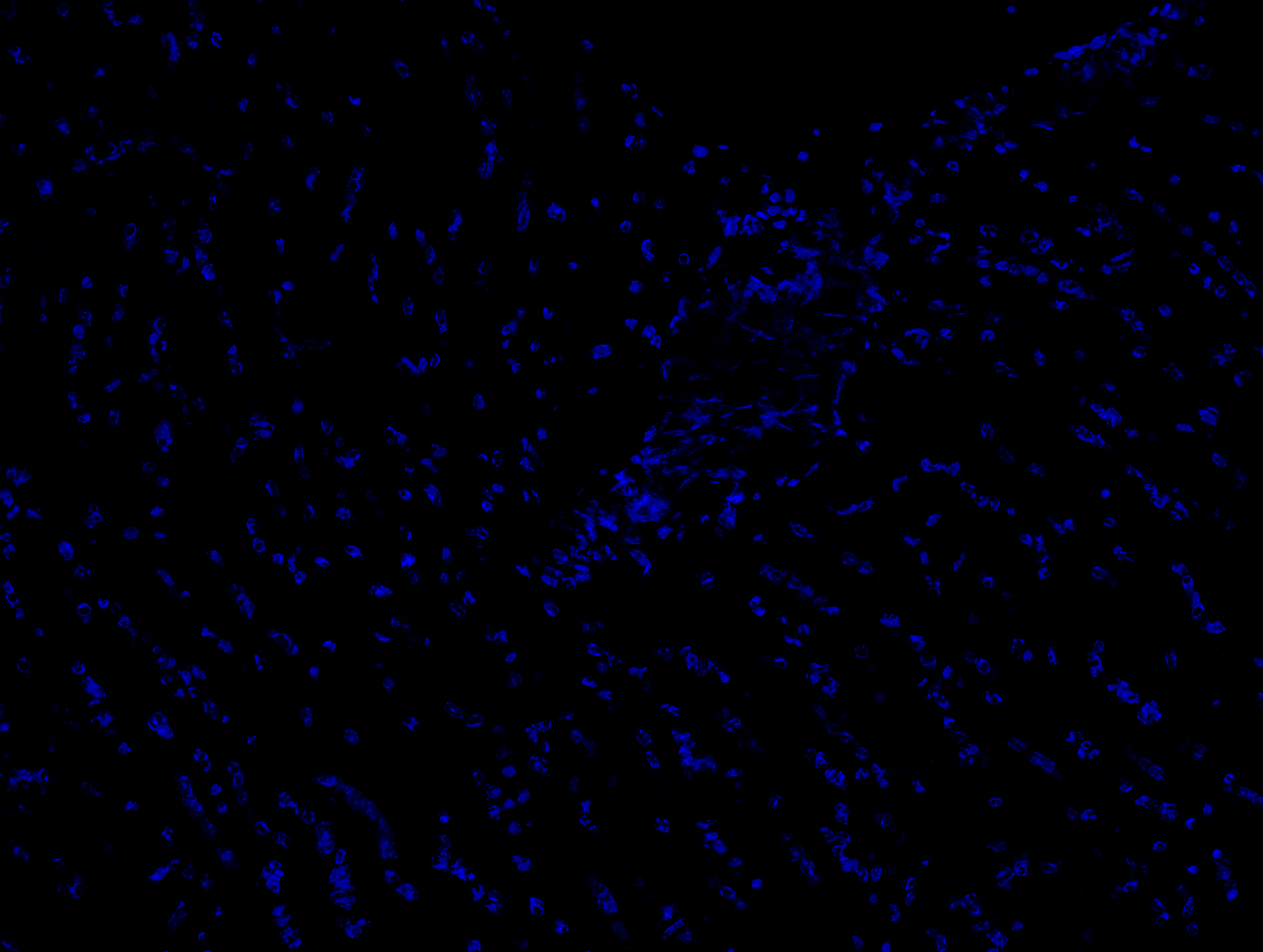

Supplement: Supplementary file 6 — Source Data Fig. 5 [file 44321_2024_49_MOESM6_ESM.zip › Figure 5/5A/EXO-EAAPMO/T DAPI.tif]

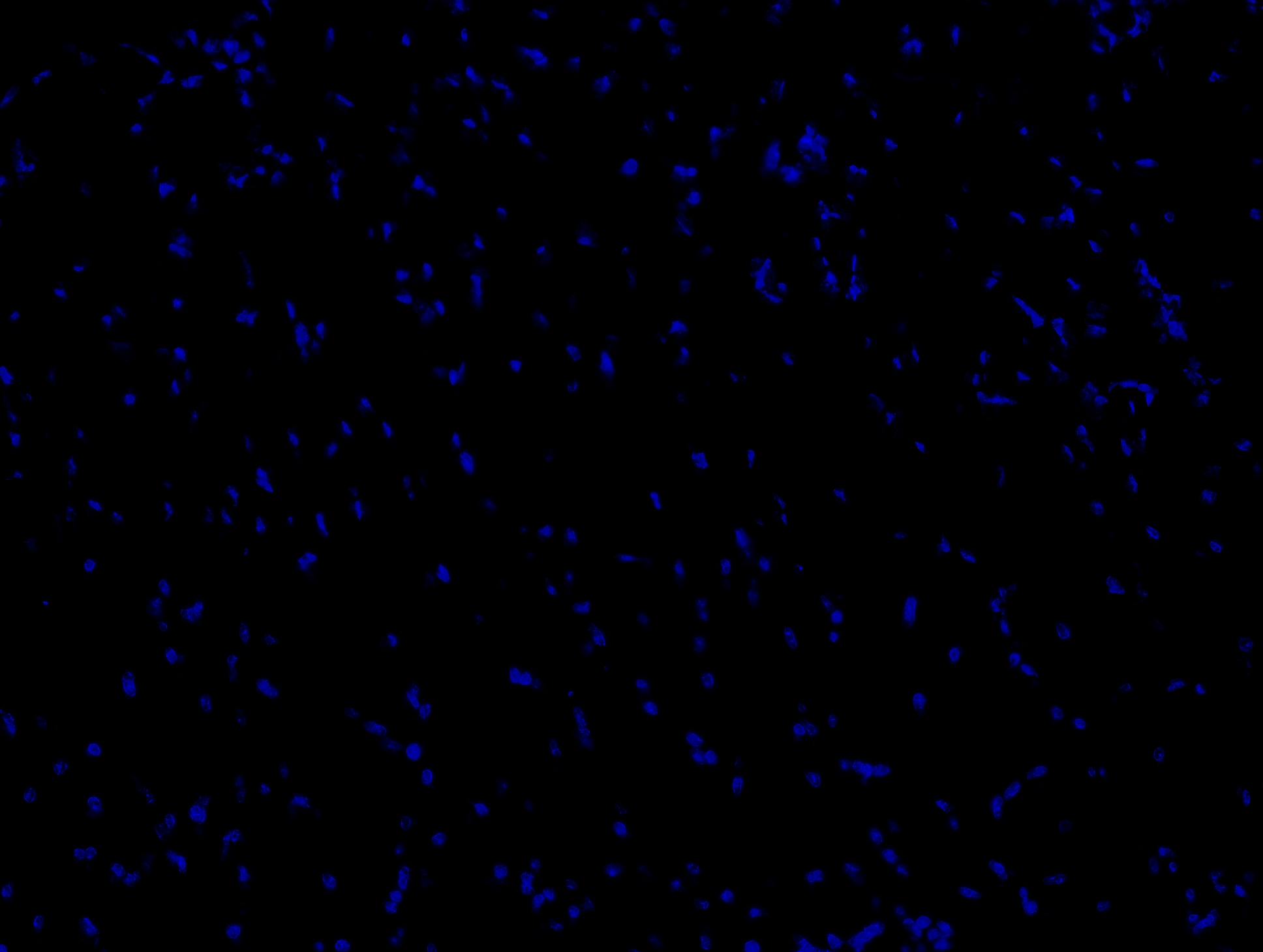

Supplement: Supplementary file 6 — Source Data Fig. 5 [file 44321_2024_49_MOESM6_ESM.zip › Figure 5/5A/EXO-EAAPMO/A DAPI.tif]

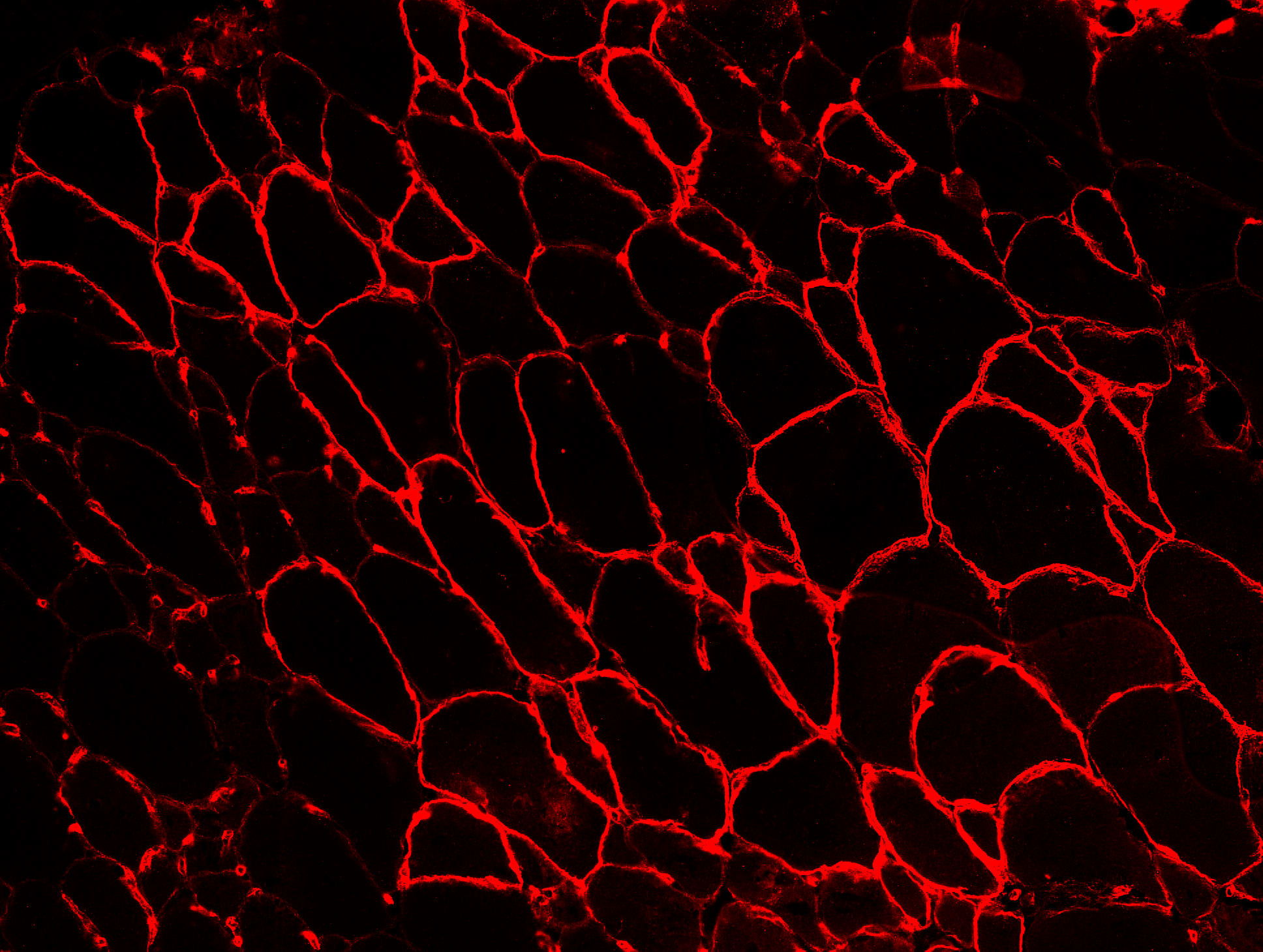

Supplement: Supplementary file 6 — Source Data Fig. 5 [file 44321_2024_49_MOESM6_ESM.zip › Figure 5/5A/EXO-EAAPMO/A DYS .tif]

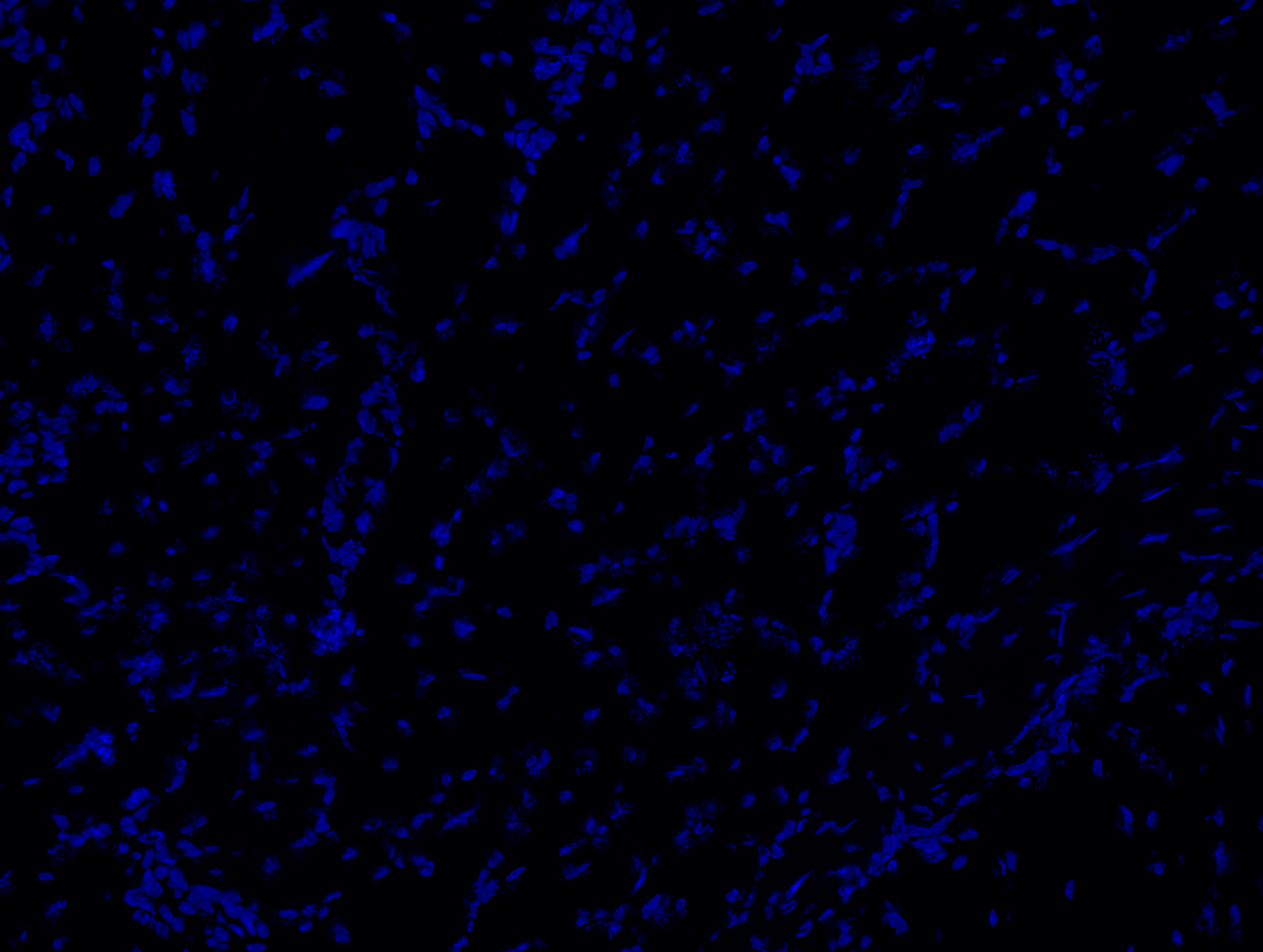

Supplement: Supplementary file 6 — Source Data Fig. 5 [file 44321_2024_49_MOESM6_ESM.zip › Figure 5/5A/EXO-EAAPMO/G DAPI.tif]

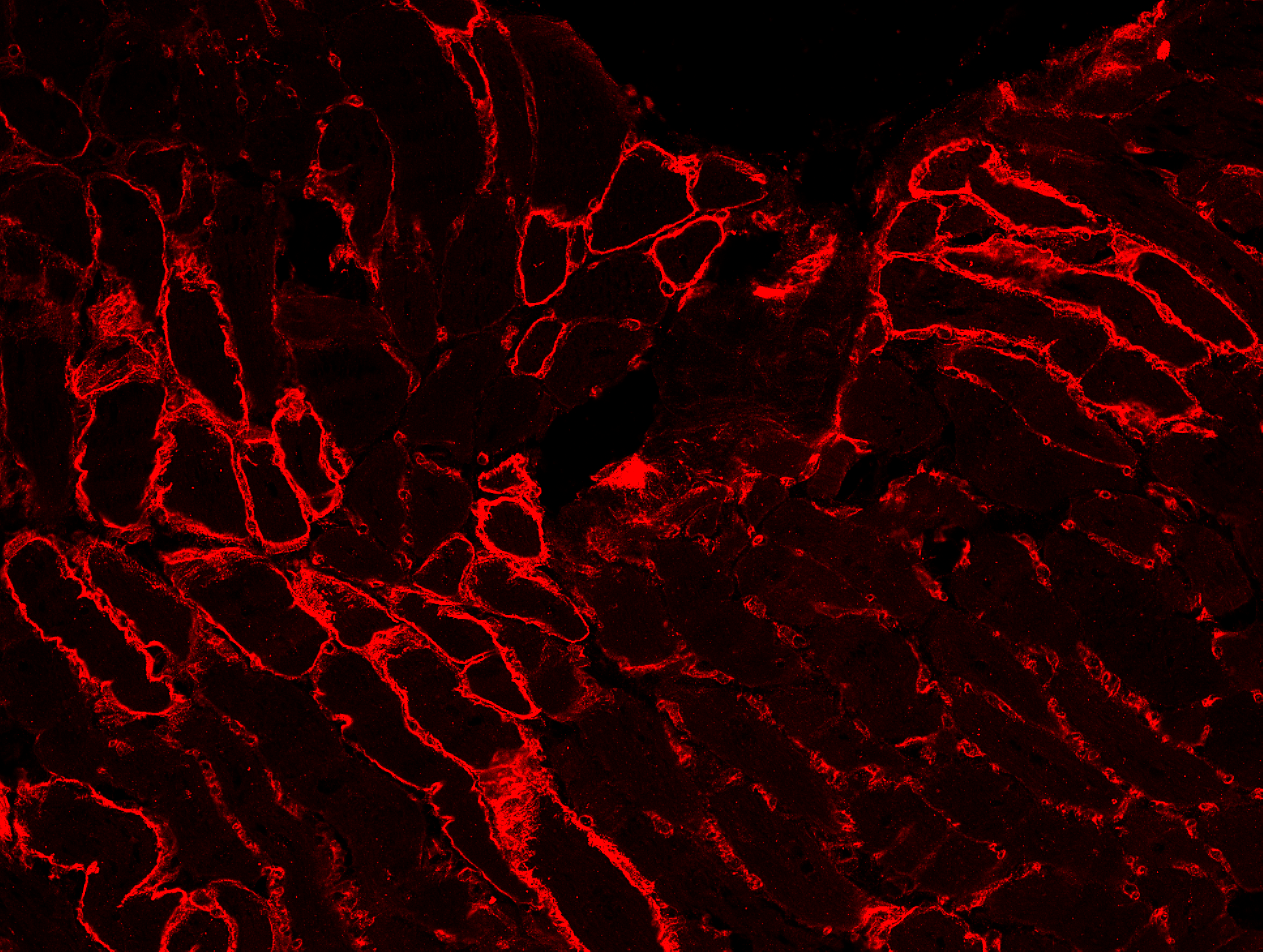

Supplement: Supplementary file 6 — Source Data Fig. 5 [file 44321_2024_49_MOESM6_ESM.zip › Figure 5/5A/EXO-EAAPMO/T DYS .tif]

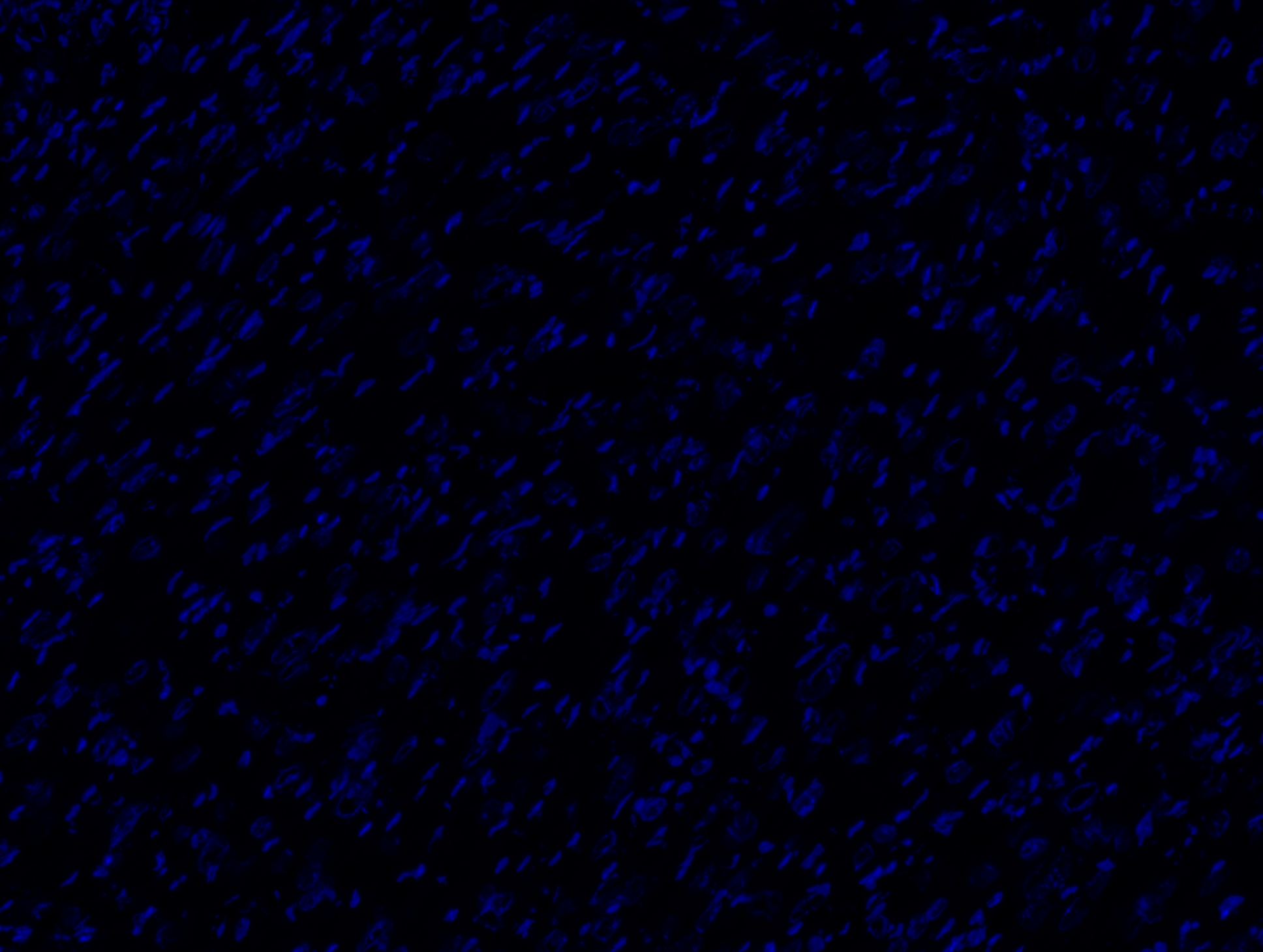

Supplement: Supplementary file 6 — Source Data Fig. 5 [file 44321_2024_49_MOESM6_ESM.zip › Figure 5/5A/EXO-EAAPMO/H DAPI.tif]

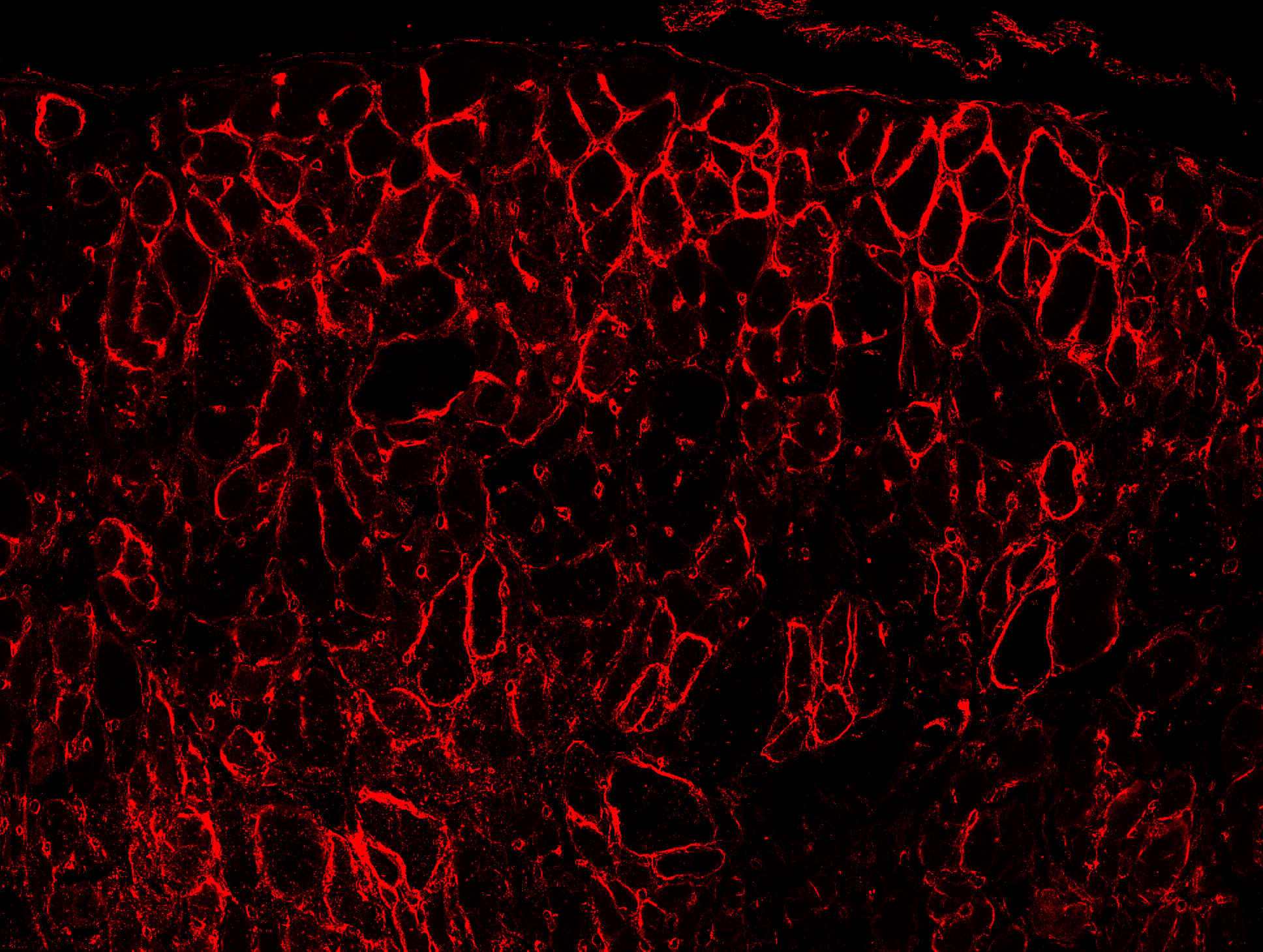

Supplement: Supplementary file 6 — Source Data Fig. 5 [file 44321_2024_49_MOESM6_ESM.zip › Figure 5/5A/EXO-EAAPMO/D DYS .tif]

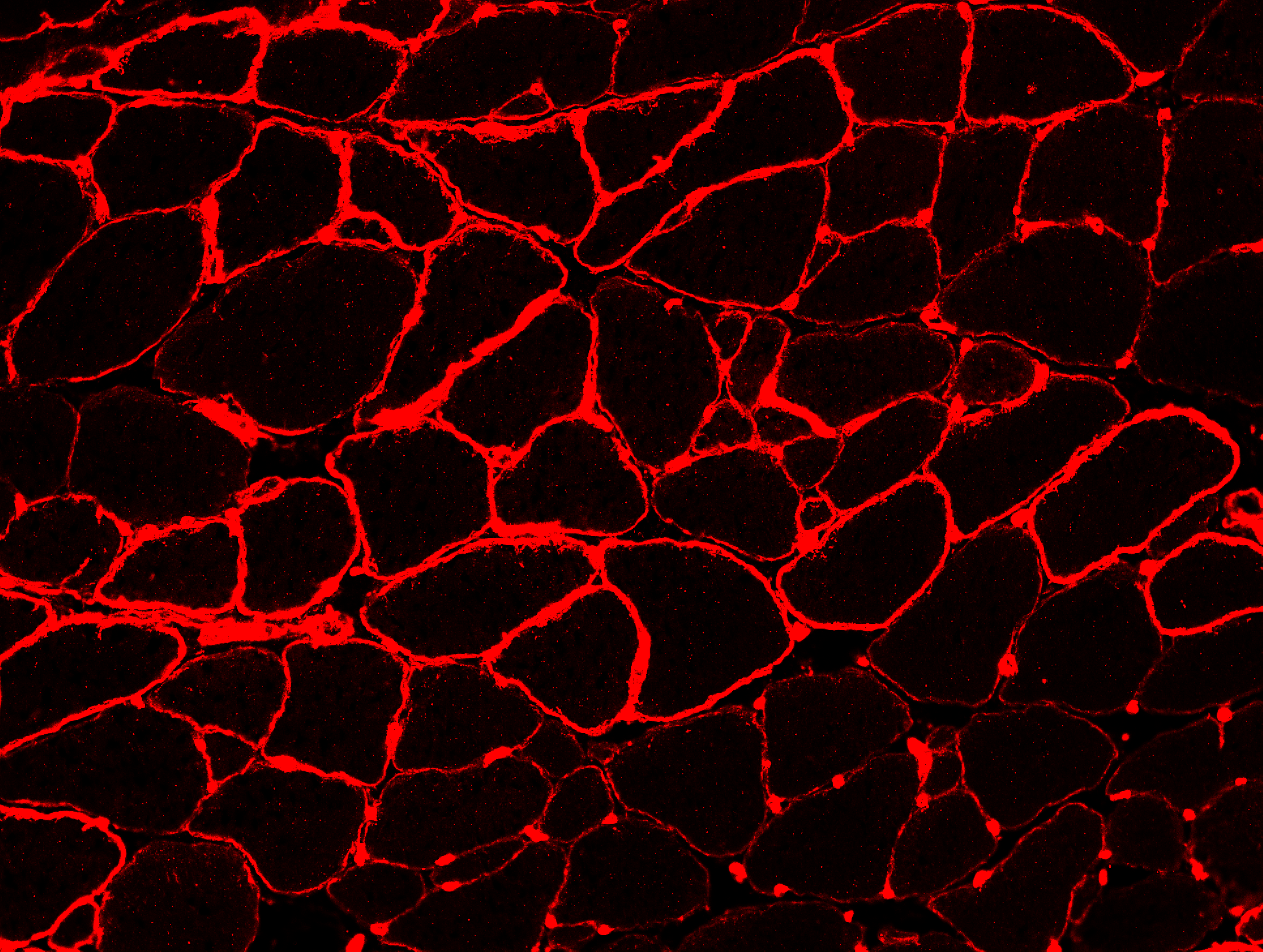

Supplement: Supplementary file 6 — Source Data Fig. 5 [file 44321_2024_49_MOESM6_ESM.zip › Figure 5/5A/EXO-EAAPMO/Q DYS .tif]

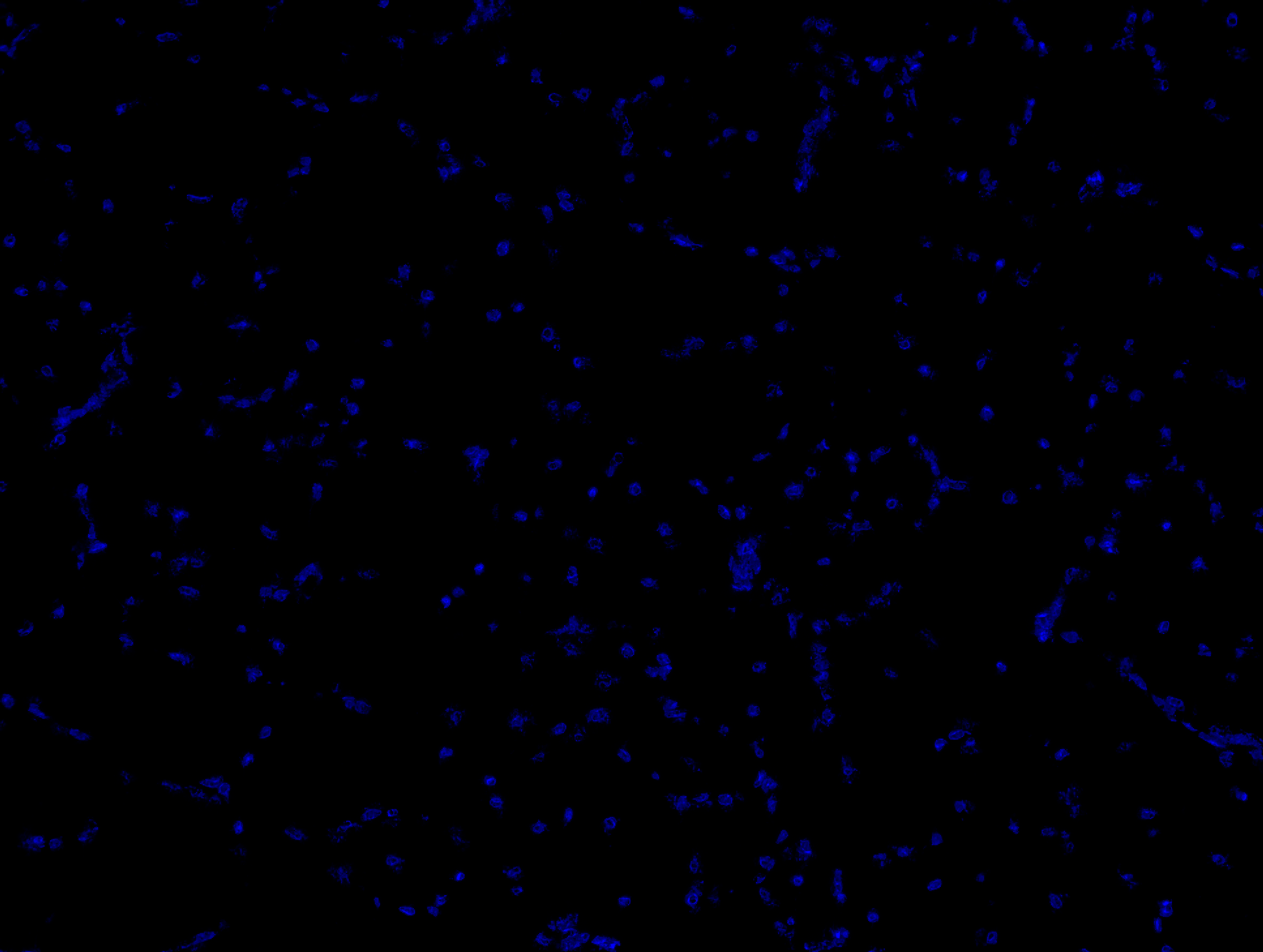

Supplement: Supplementary file 6 — Source Data Fig. 5 [file 44321_2024_49_MOESM6_ESM.zip › Figure 5/5A/EXO-EAAPMO/TA DAPI.tif]

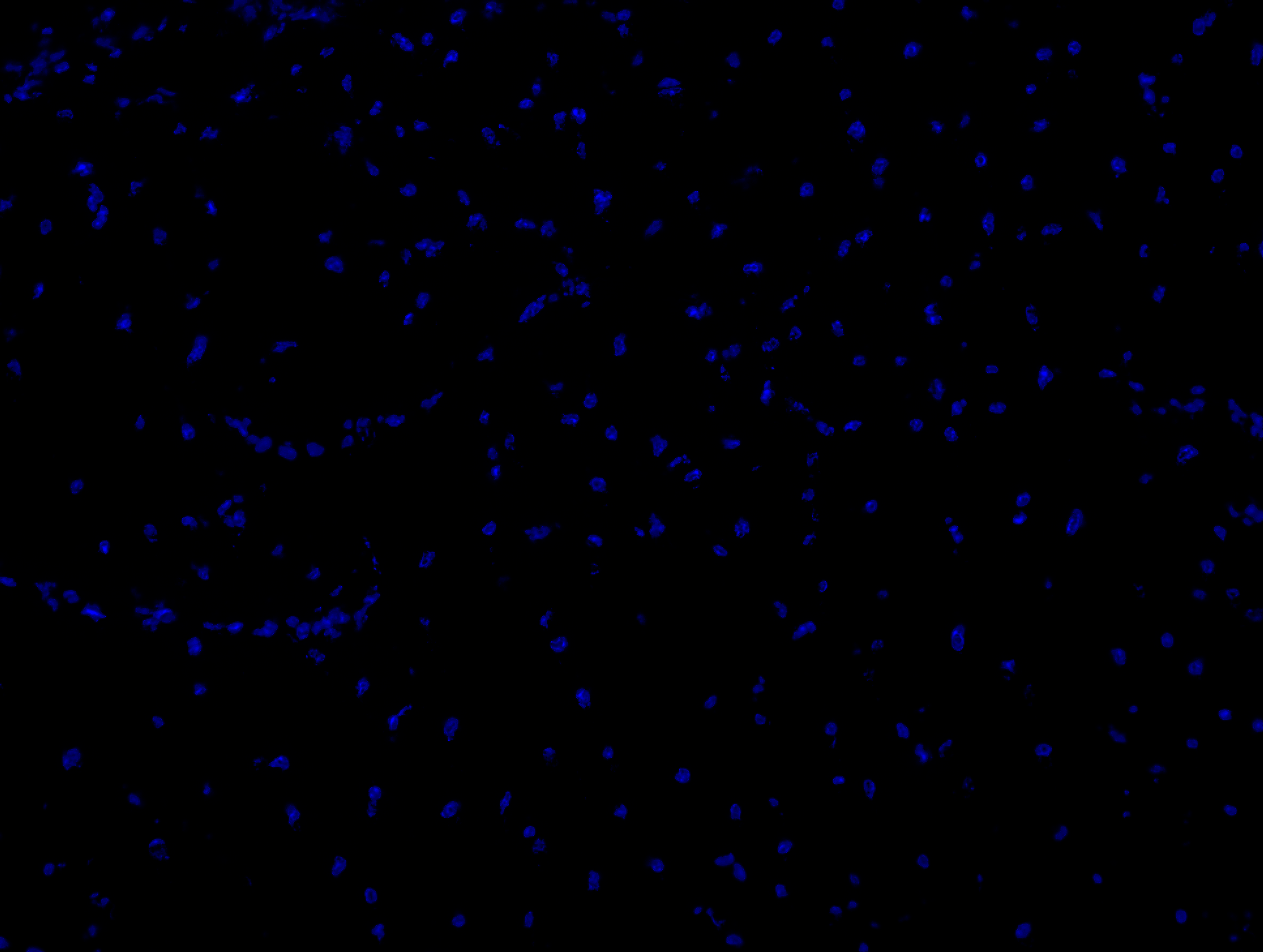

Supplement: Supplementary file 6 — Source Data Fig. 5 [file 44321_2024_49_MOESM6_ESM.zip › Figure 5/5A/EXO-EAAPMO/Q DAPI.tif]

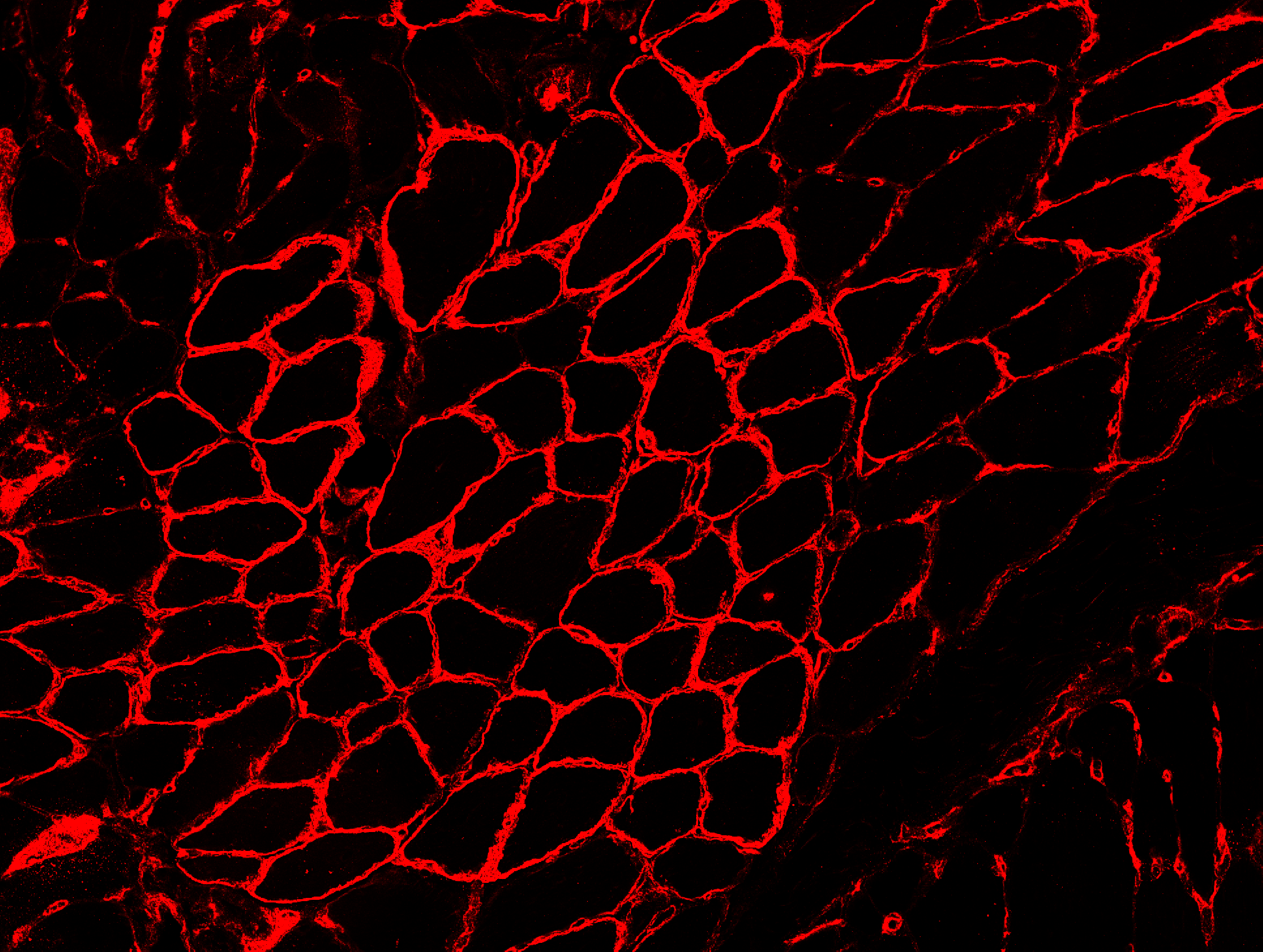

Supplement: Supplementary file 6 — Source Data Fig. 5 [file 44321_2024_49_MOESM6_ESM.zip › Figure 5/5A/EXO-EAAPMO/G DYS.tif]

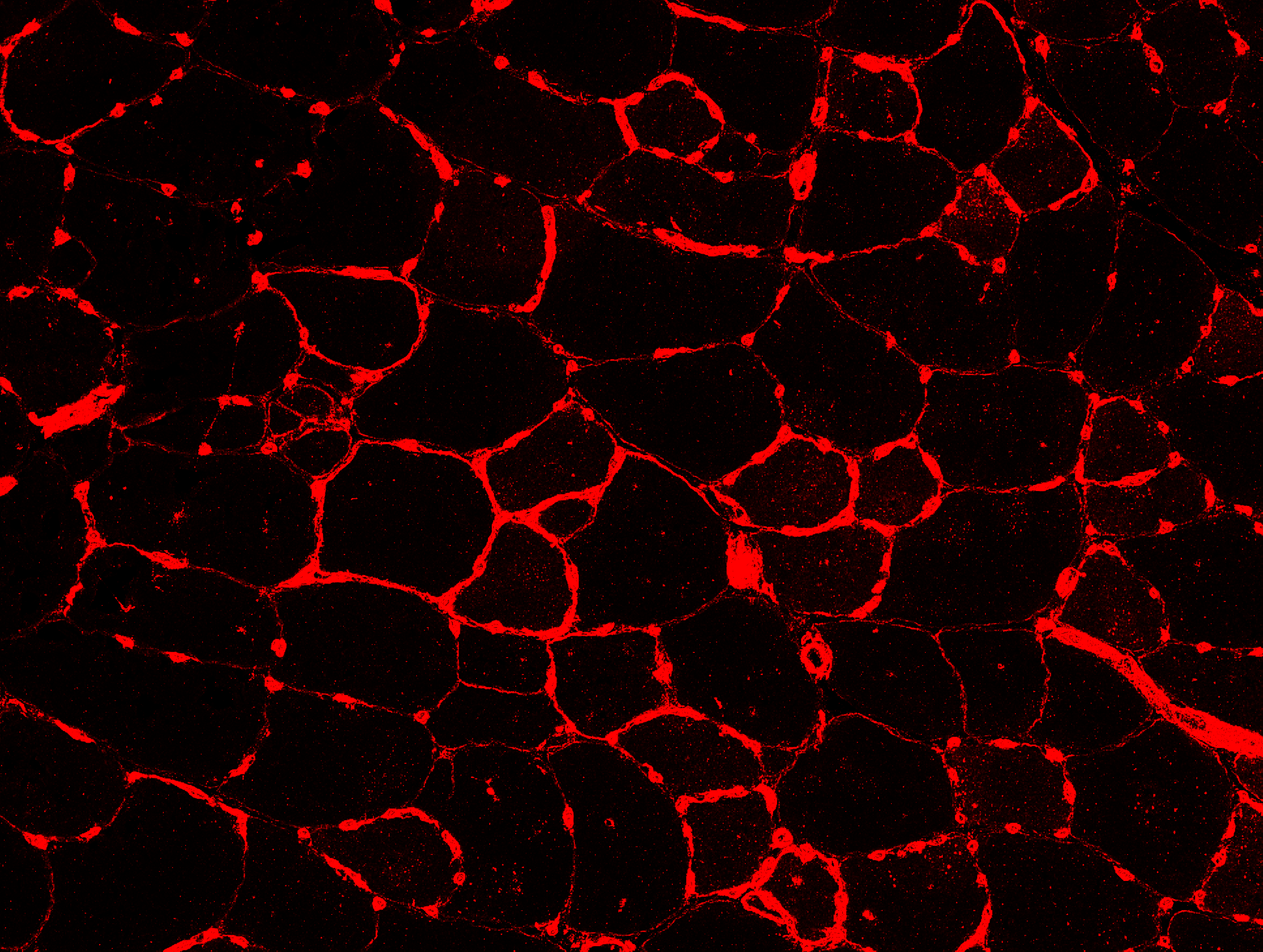

Supplement: Supplementary file 6 — Source Data Fig. 5 [file 44321_2024_49_MOESM6_ESM.zip › Figure 5/5A/EXO-EAAPMO/TA DYS.tif]

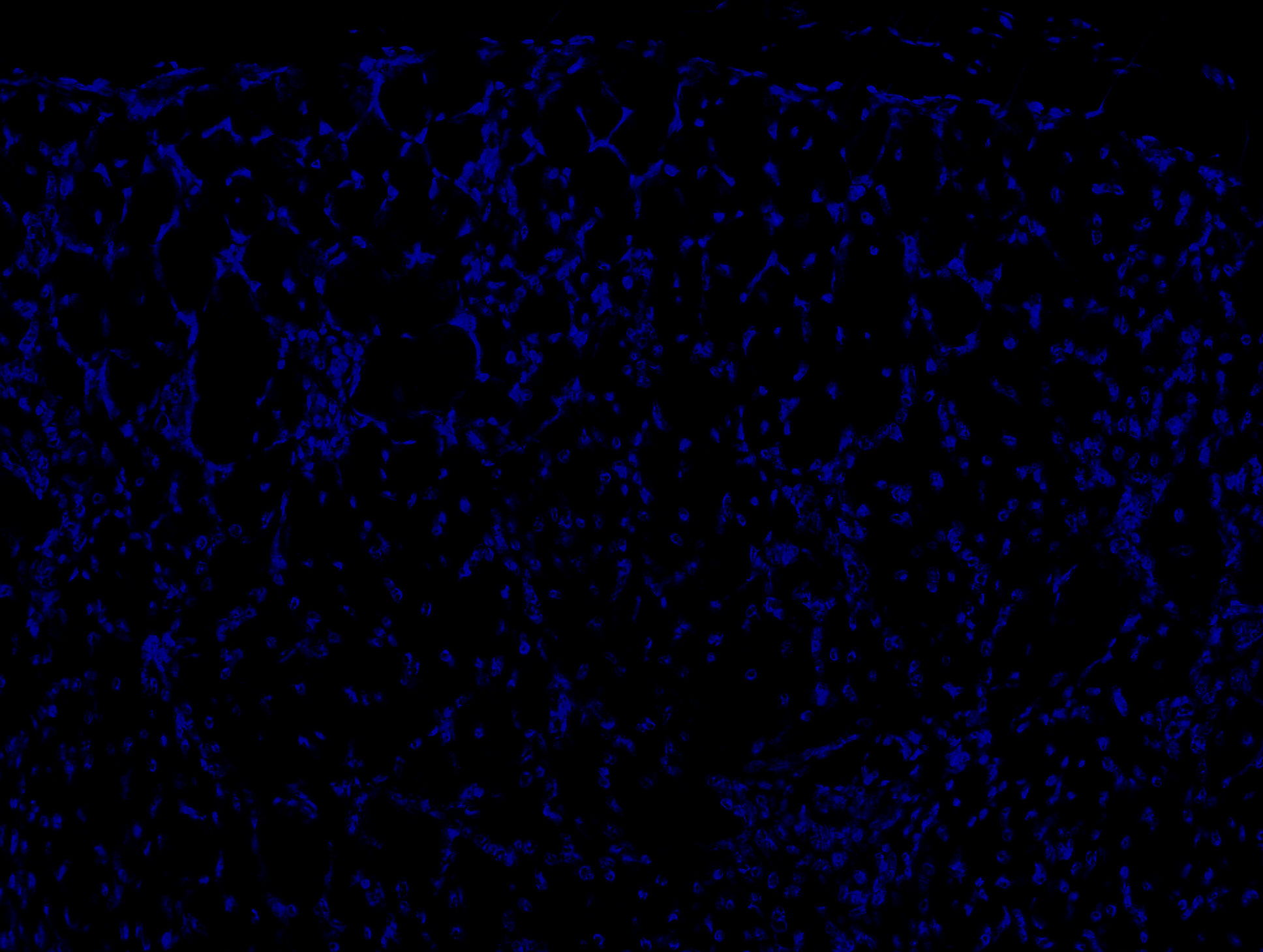

Supplement: Supplementary file 6 — Source Data Fig. 5 [file 44321_2024_49_MOESM6_ESM.zip › Figure 5/5A/EXO-EAAPMO/D DAPI.tif]

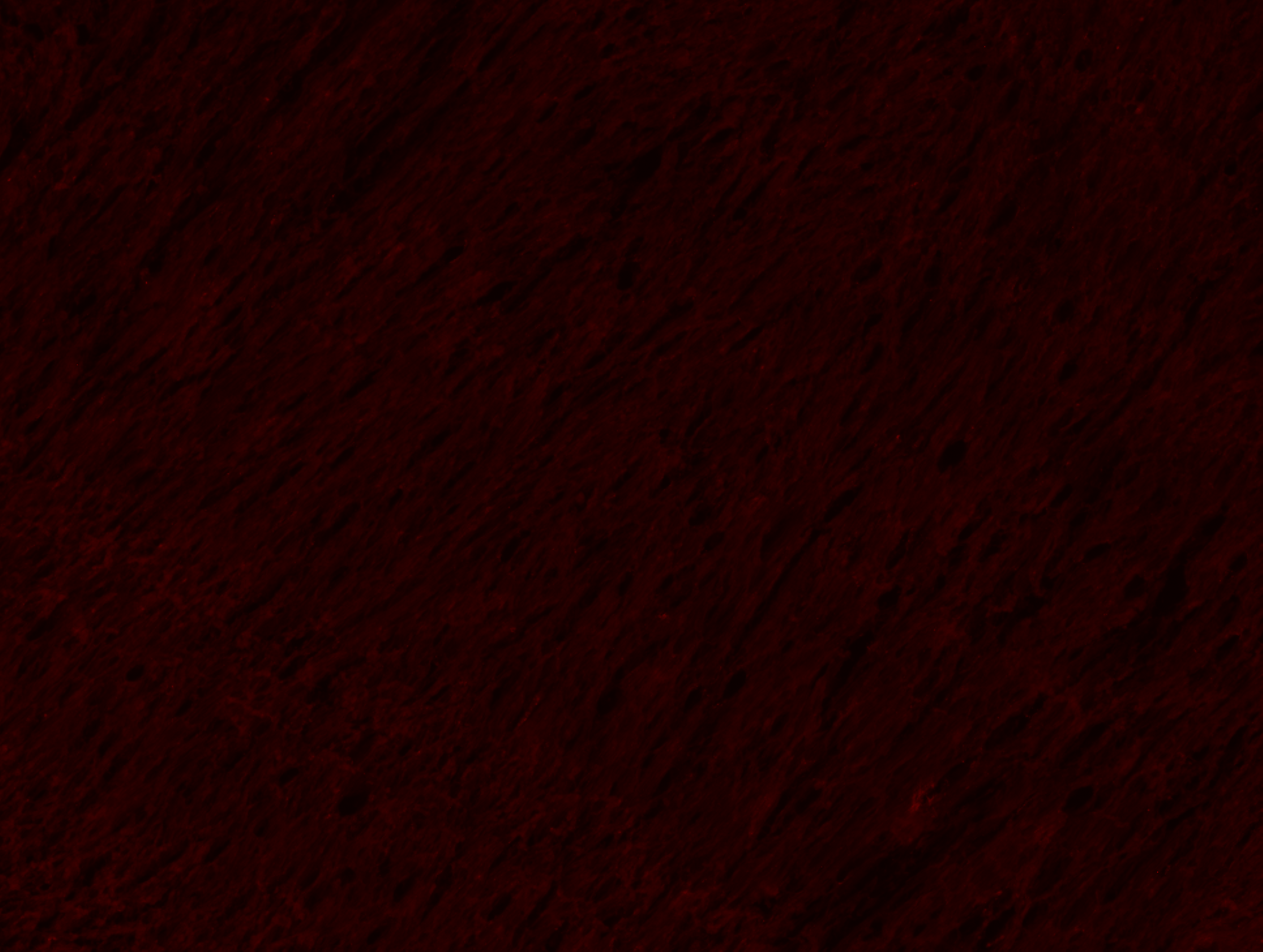

Supplement: Supplementary file 6 — Source Data Fig. 5 [file 44321_2024_49_MOESM6_ESM.zip › Figure 5/5A/EXO-EAAPMO/H DYS .tif]

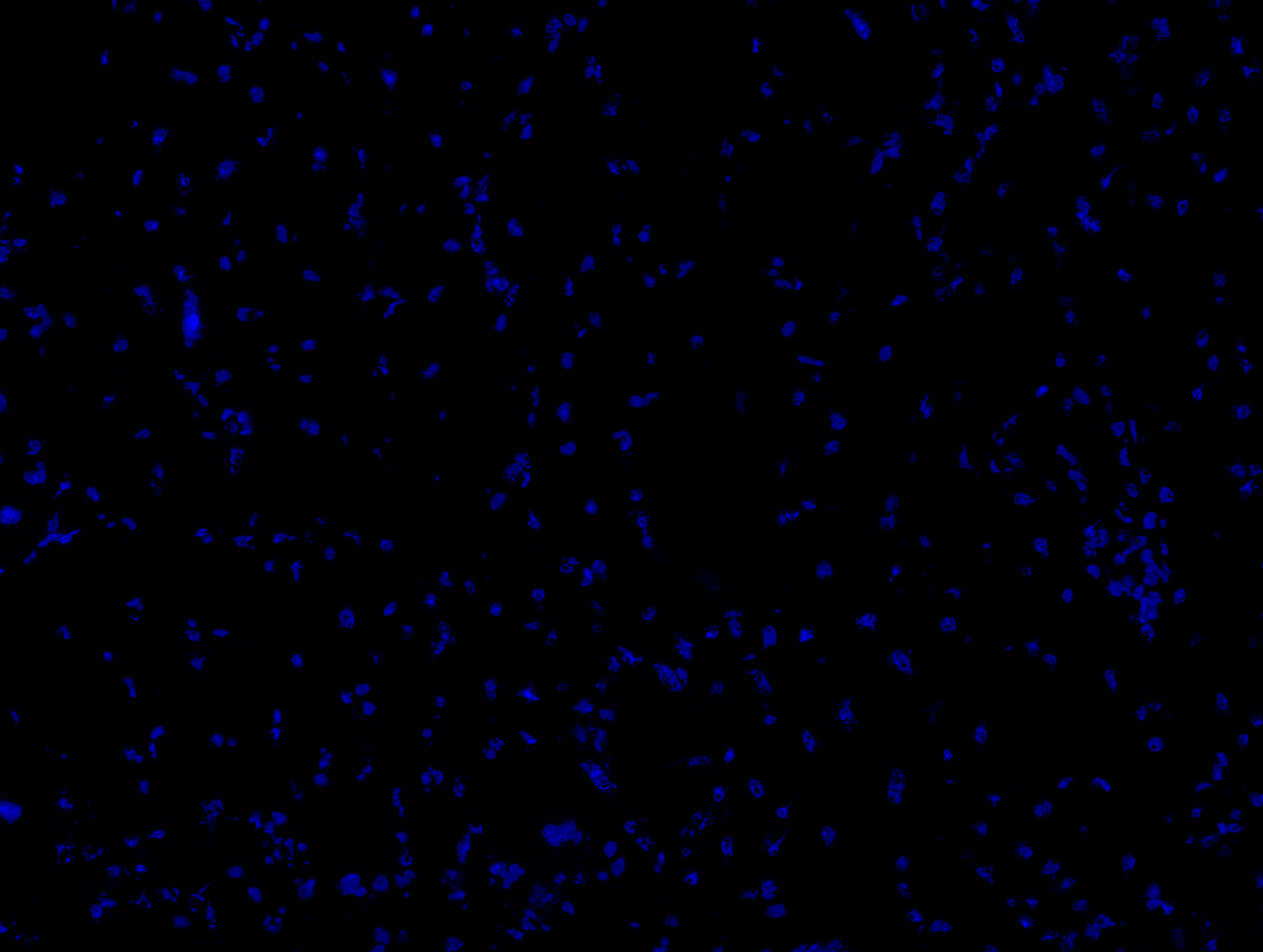

Supplement: Supplementary file 6 — Source Data Fig. 5 [file 44321_2024_49_MOESM6_ESM.zip › Figure 5/5A/PMO/T DAPI.tif]

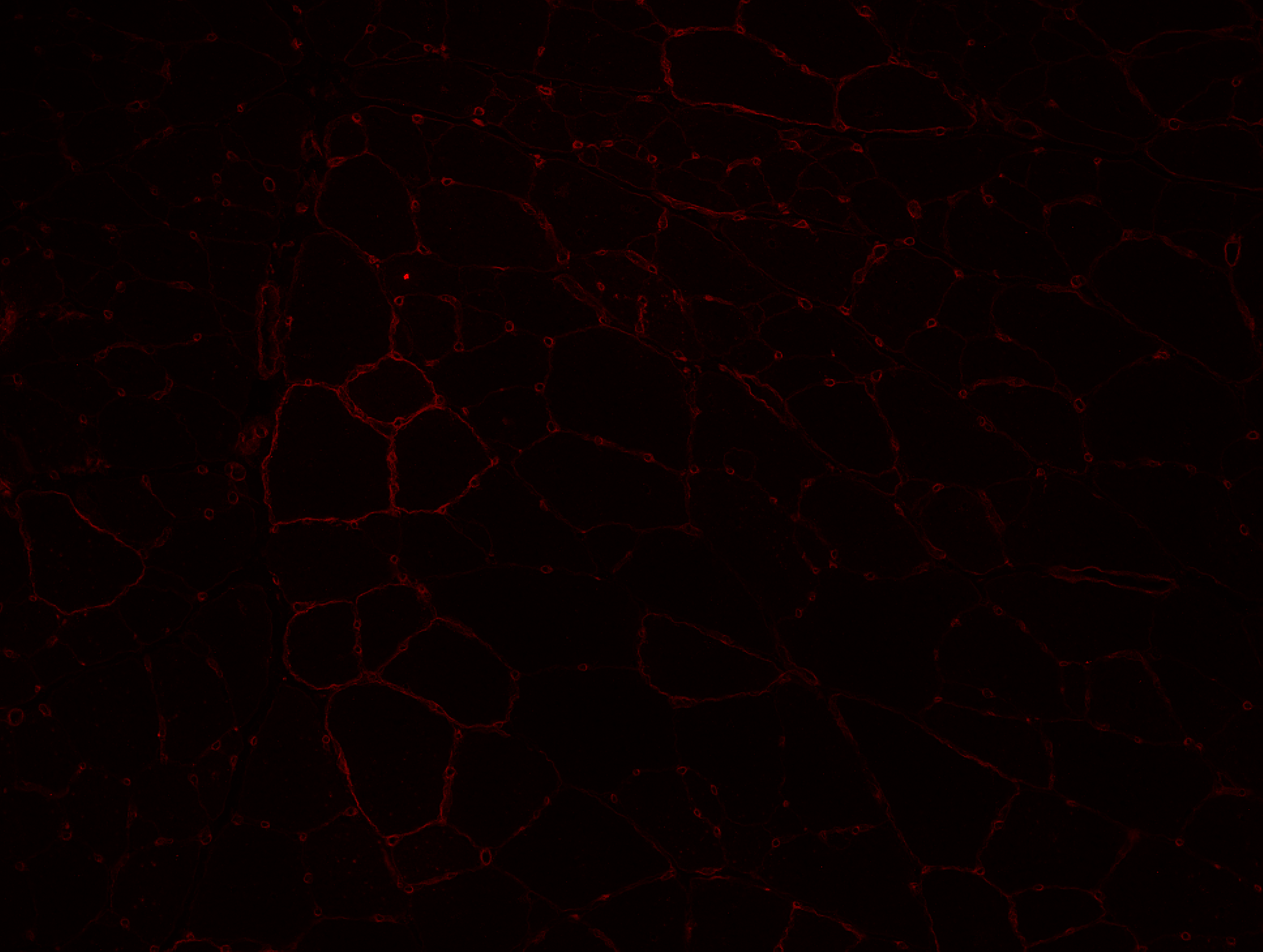

Supplement: Supplementary file 6 — Source Data Fig. 5 [file 44321_2024_49_MOESM6_ESM.zip › Figure 5/5A/PMO/G DYS .tif]

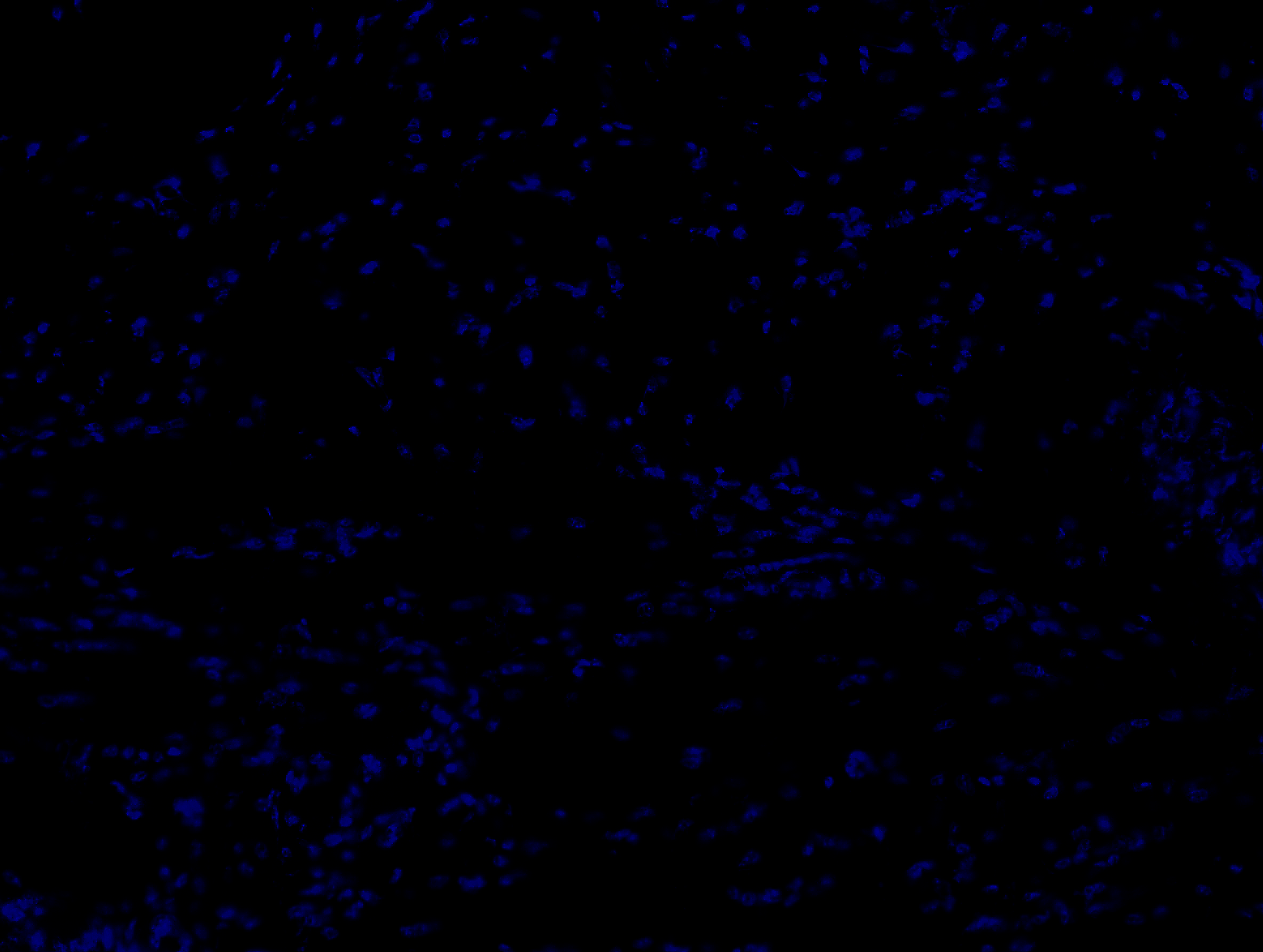

Supplement: Supplementary file 6 — Source Data Fig. 5 [file 44321_2024_49_MOESM6_ESM.zip › Figure 5/5A/PMO/A DAPI.tif]

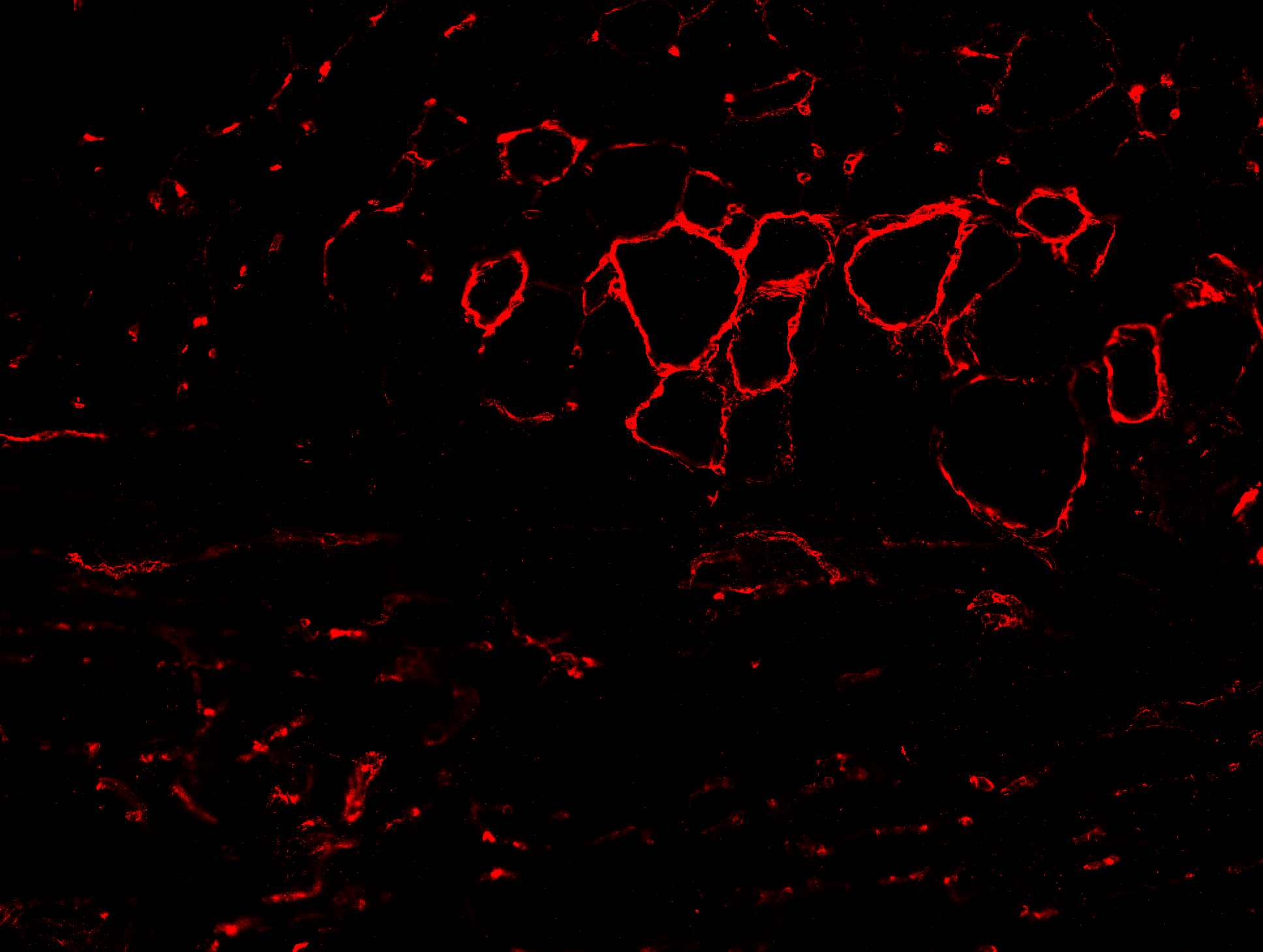

Supplement: Supplementary file 6 — Source Data Fig. 5 [file 44321_2024_49_MOESM6_ESM.zip › Figure 5/5A/PMO/A DYS .tif]

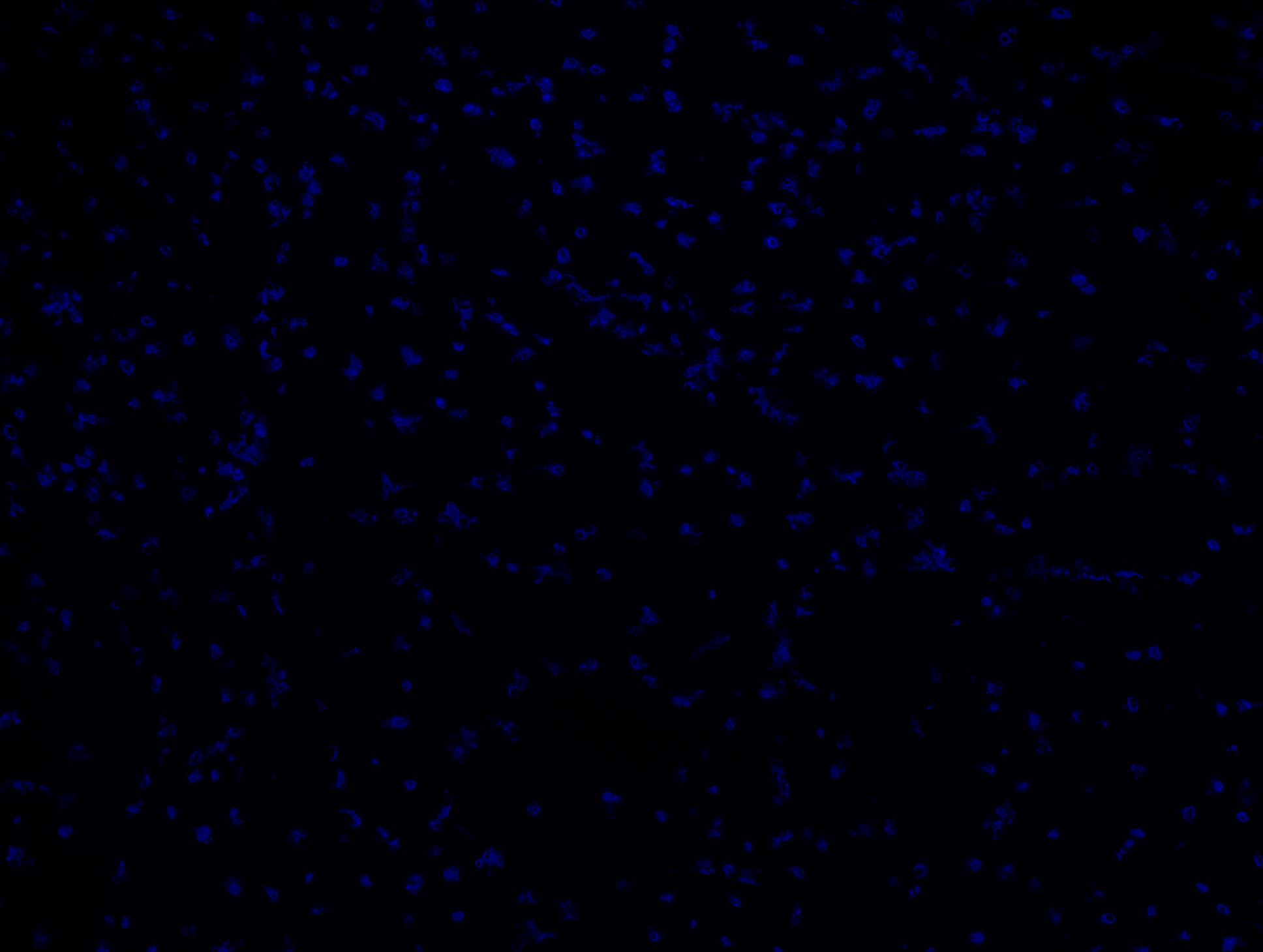

Supplement: Supplementary file 6 — Source Data Fig. 5 [file 44321_2024_49_MOESM6_ESM.zip › Figure 5/5A/PMO/G DAPI.tif]

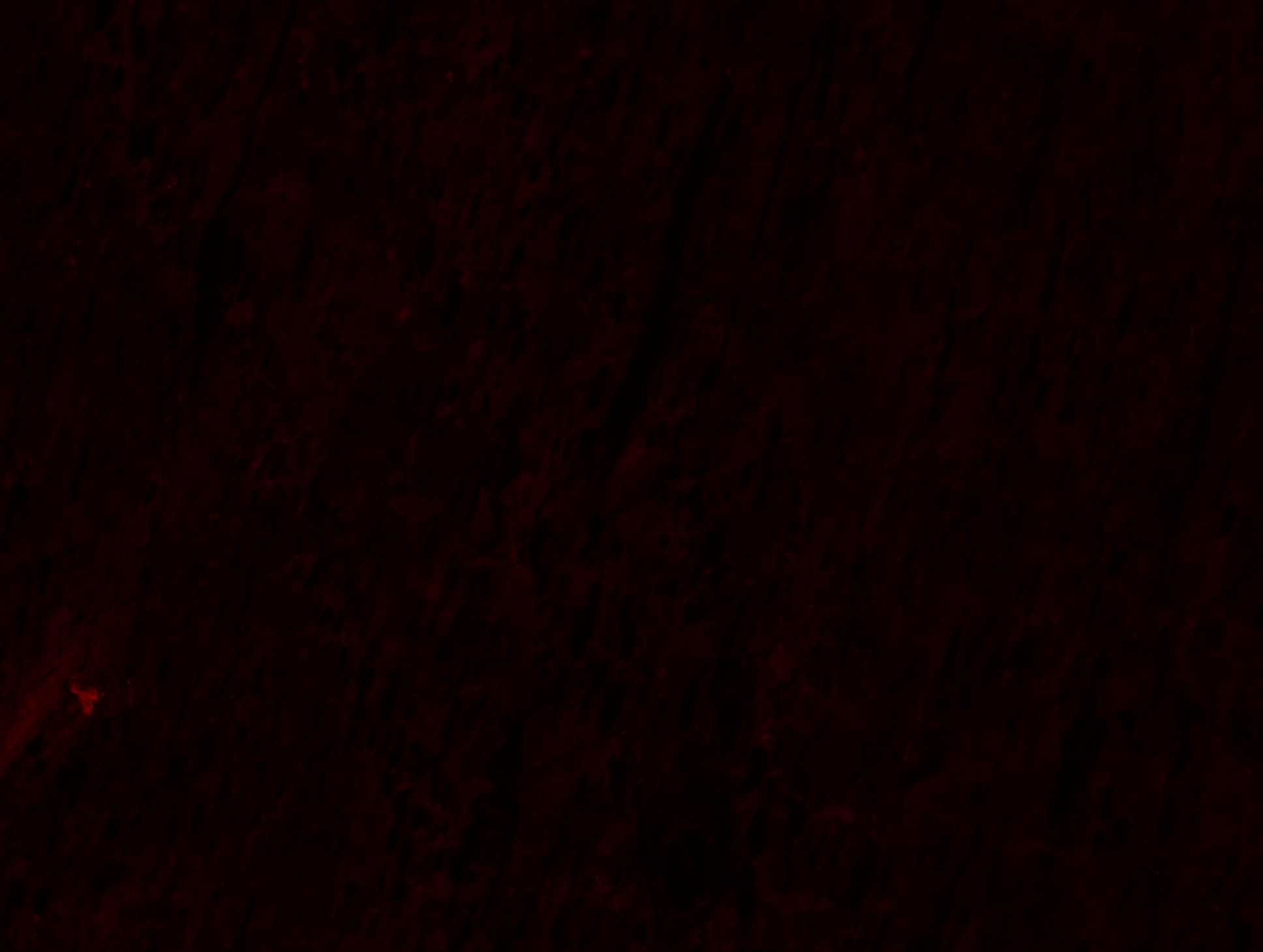

Supplement: Supplementary file 6 — Source Data Fig. 5 [file 44321_2024_49_MOESM6_ESM.zip › Figure 5/5A/PMO/H DYS.tif]

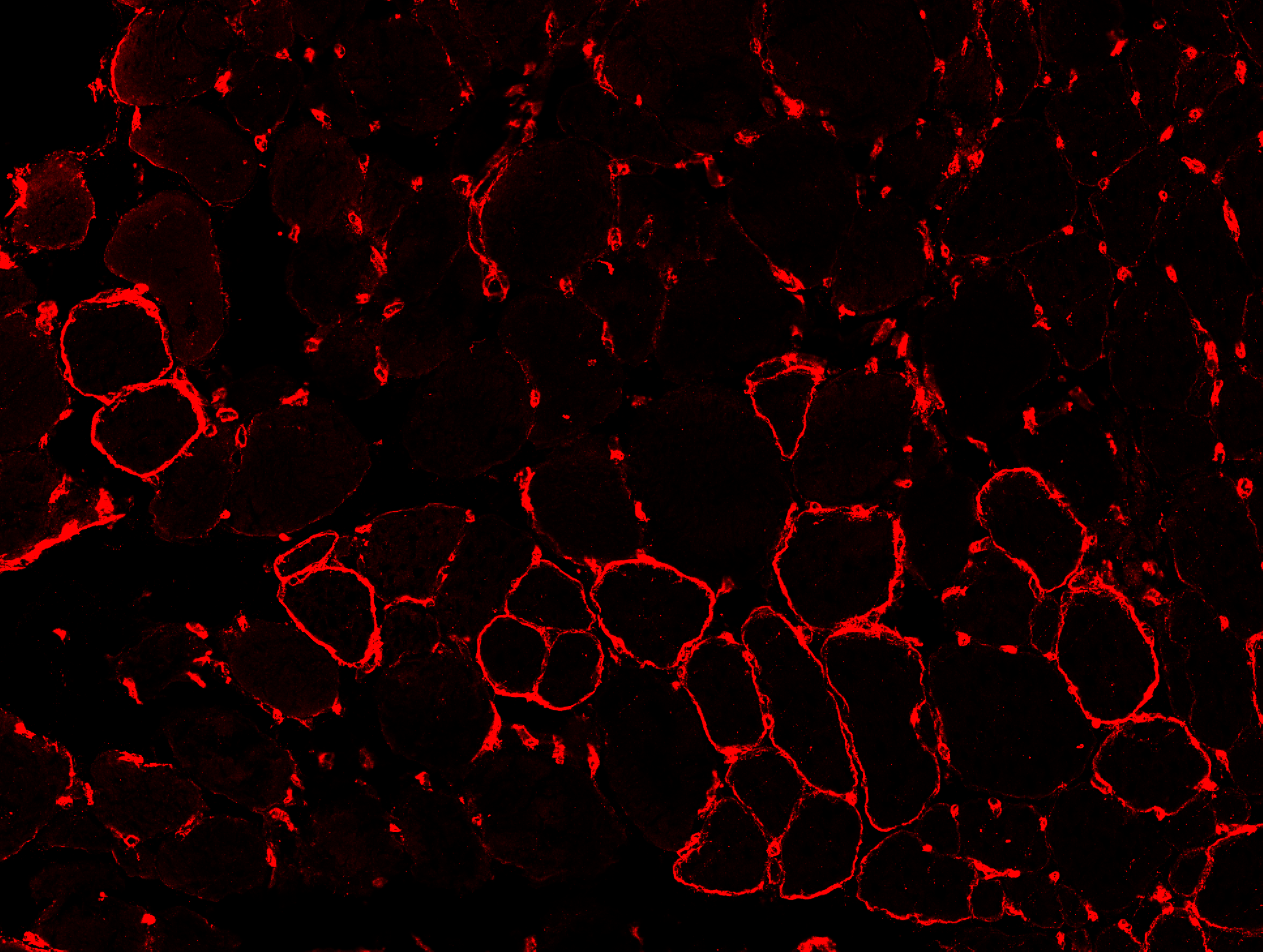

Supplement: Supplementary file 6 — Source Data Fig. 5 [file 44321_2024_49_MOESM6_ESM.zip › Figure 5/5A/PMO/T DYS .tif]

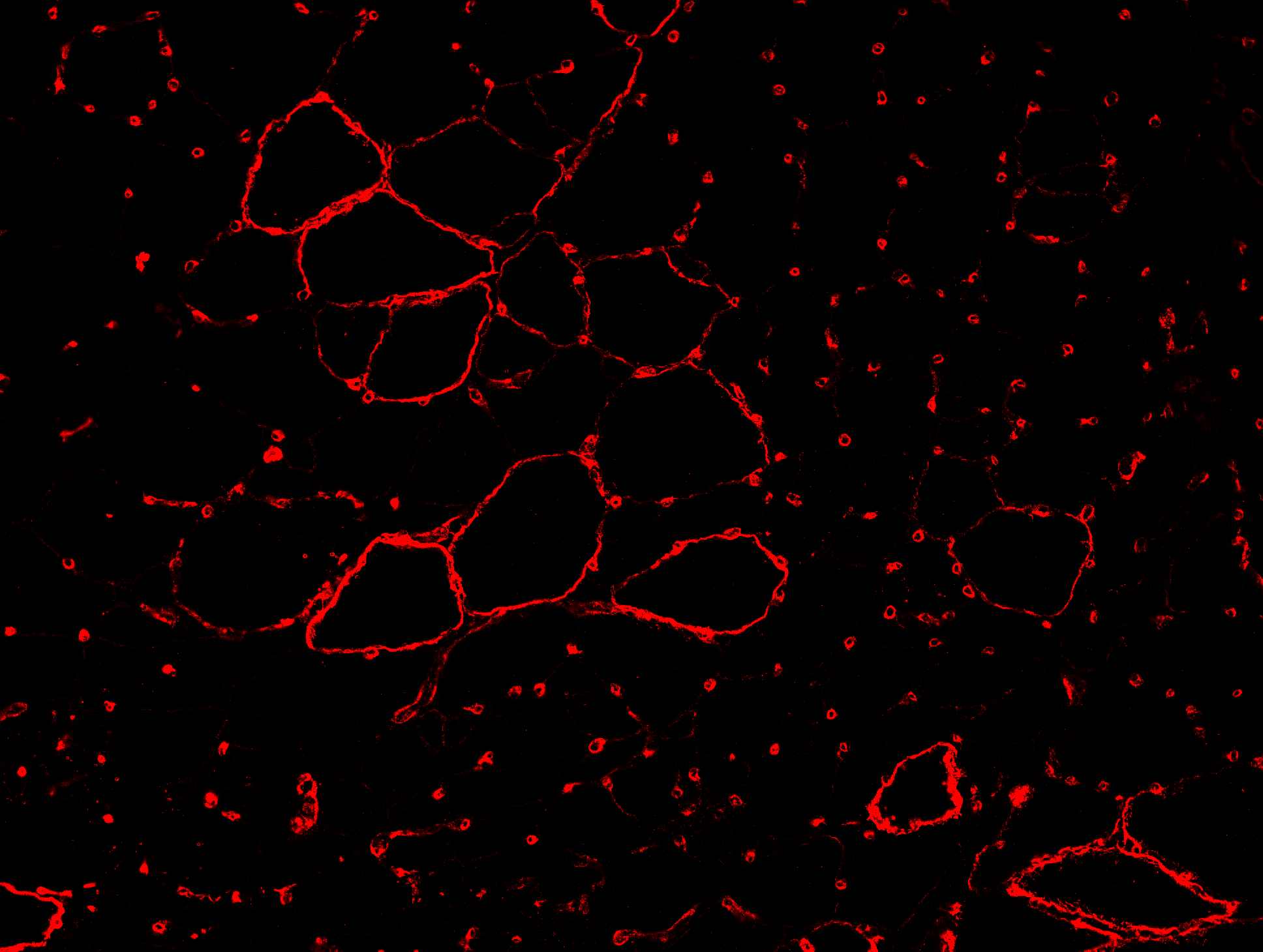

Supplement: Supplementary file 6 — Source Data Fig. 5 [file 44321_2024_49_MOESM6_ESM.zip › Figure 5/5A/PMO/Q DYS.tif]

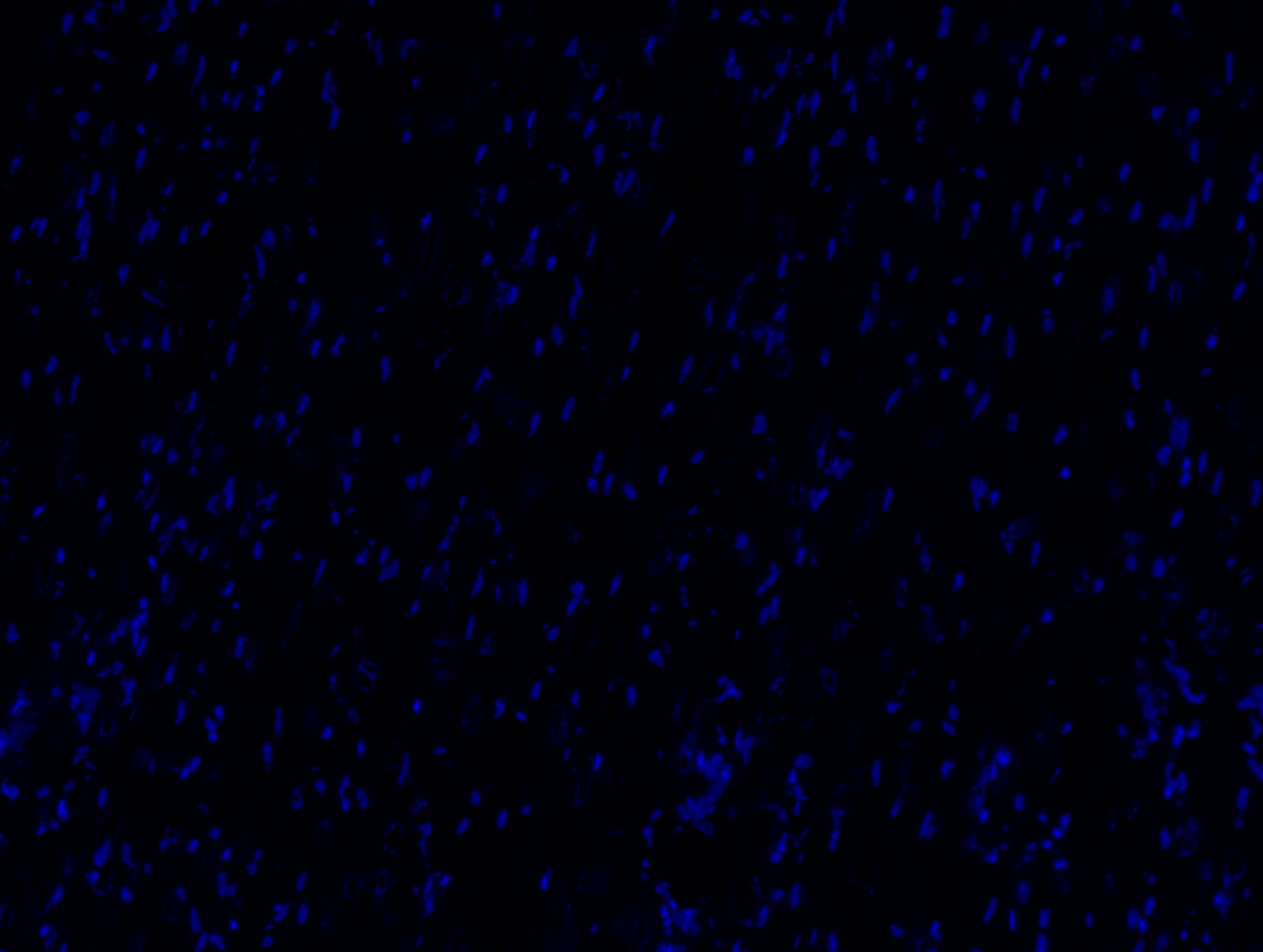

Supplement: Supplementary file 6 — Source Data Fig. 5 [file 44321_2024_49_MOESM6_ESM.zip › Figure 5/5A/PMO/H DAPI.tif]

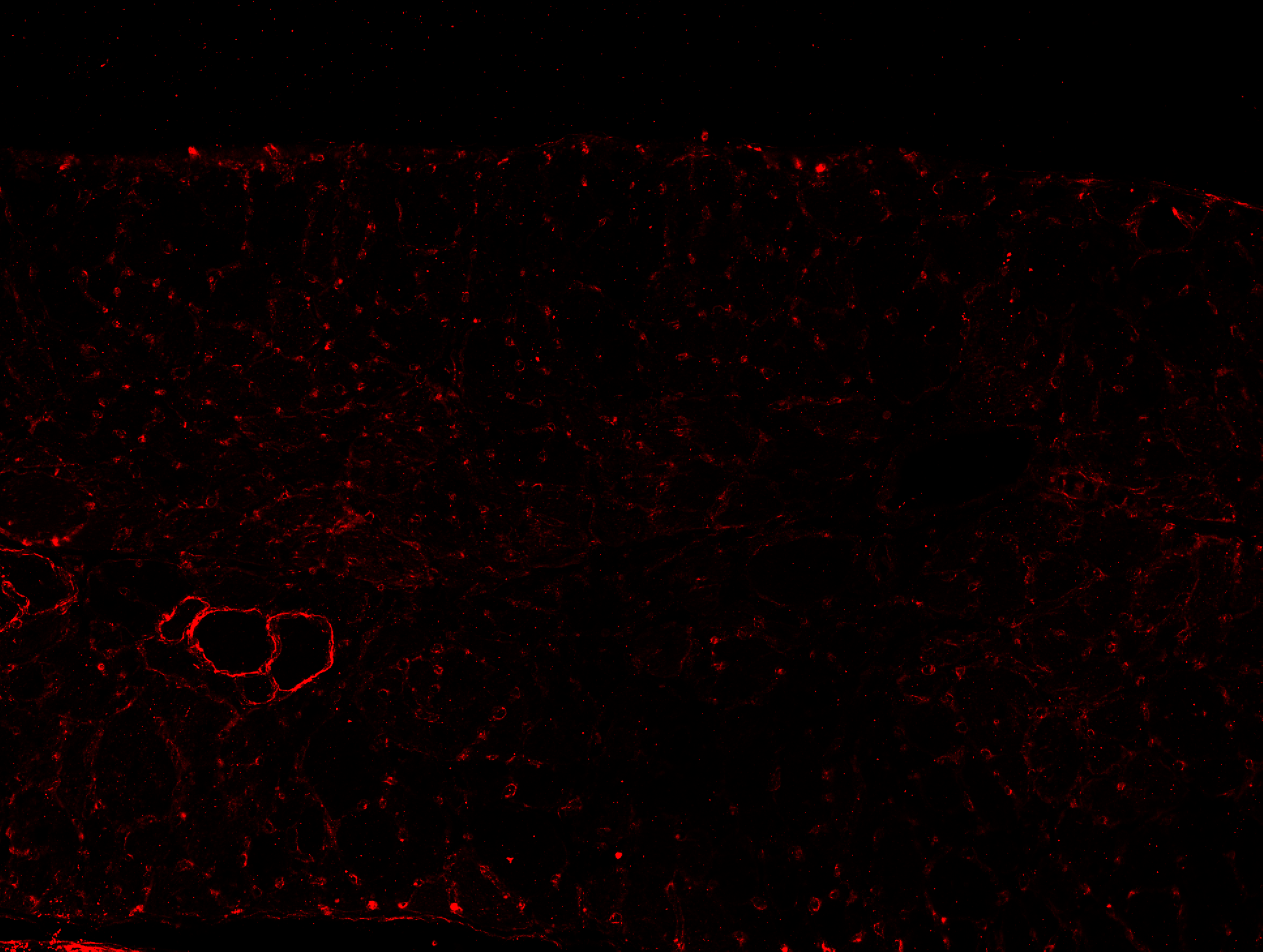

Supplement: Supplementary file 6 — Source Data Fig. 5 [file 44321_2024_49_MOESM6_ESM.zip › Figure 5/5A/PMO/D DYS .tif]

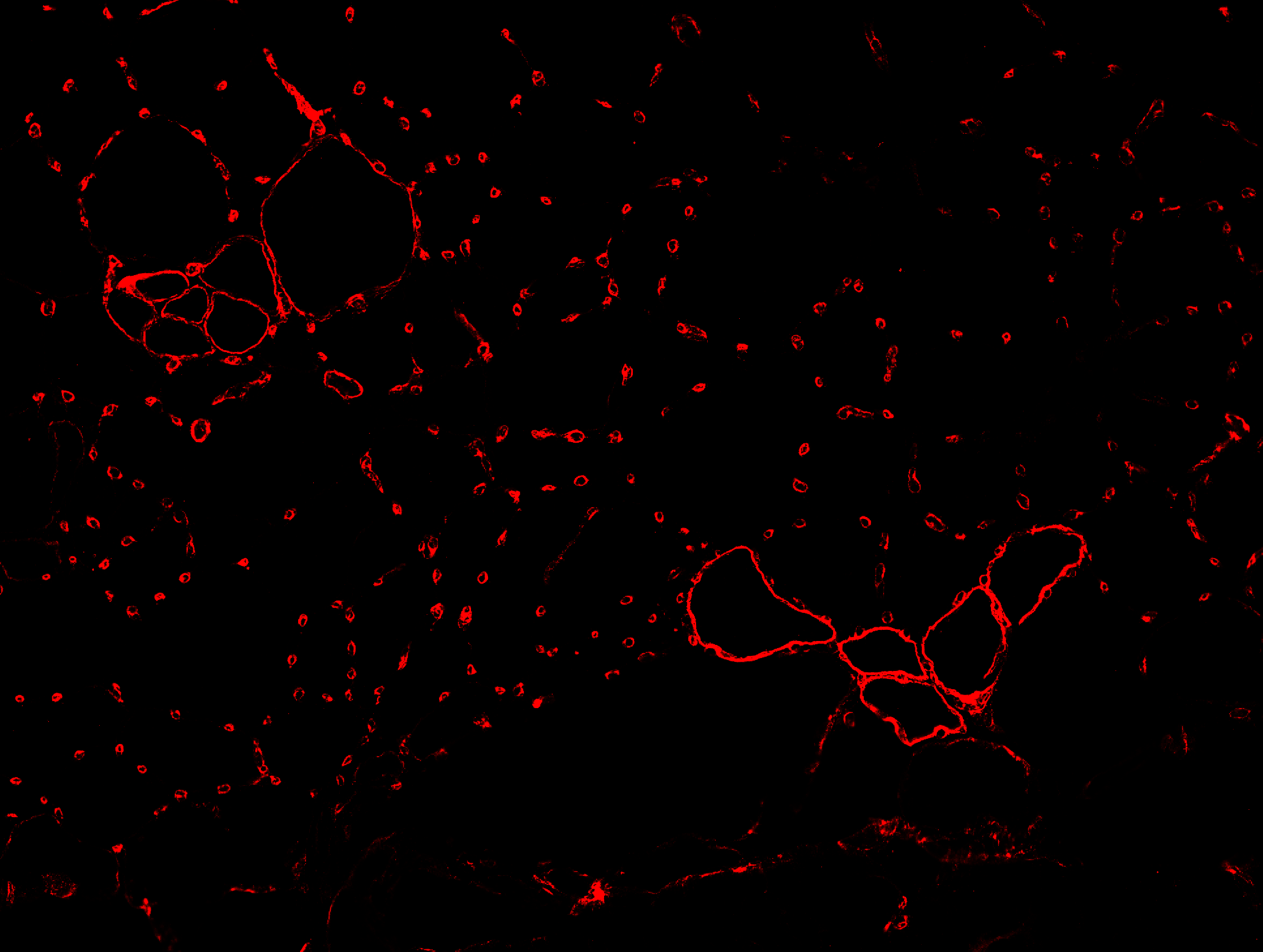

Supplement: Supplementary file 6 — Source Data Fig. 5 [file 44321_2024_49_MOESM6_ESM.zip › Figure 5/5A/PMO/TA DYS .tif]

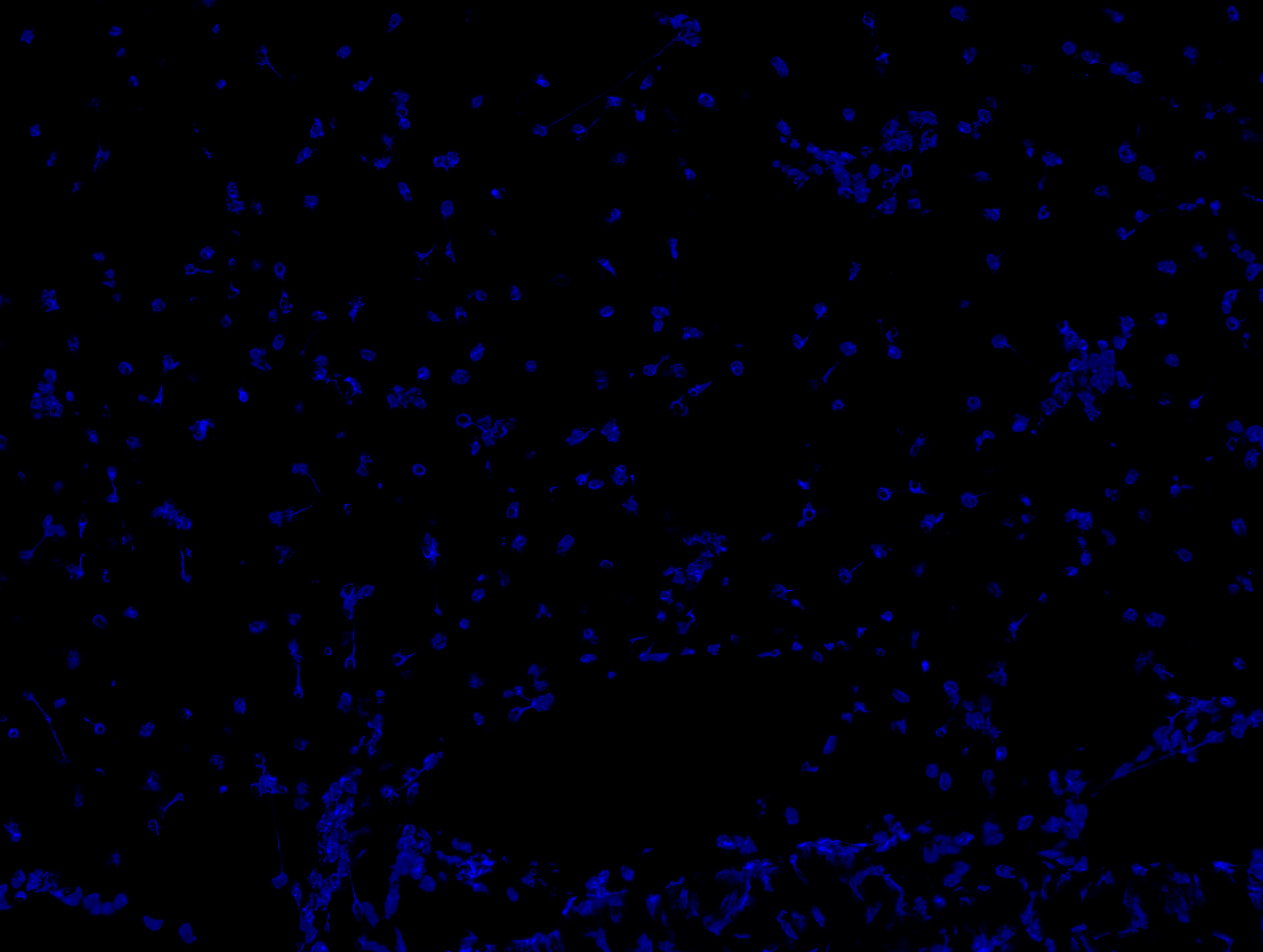

Supplement: Supplementary file 6 — Source Data Fig. 5 [file 44321_2024_49_MOESM6_ESM.zip › Figure 5/5A/PMO/TA DAPI.tif]

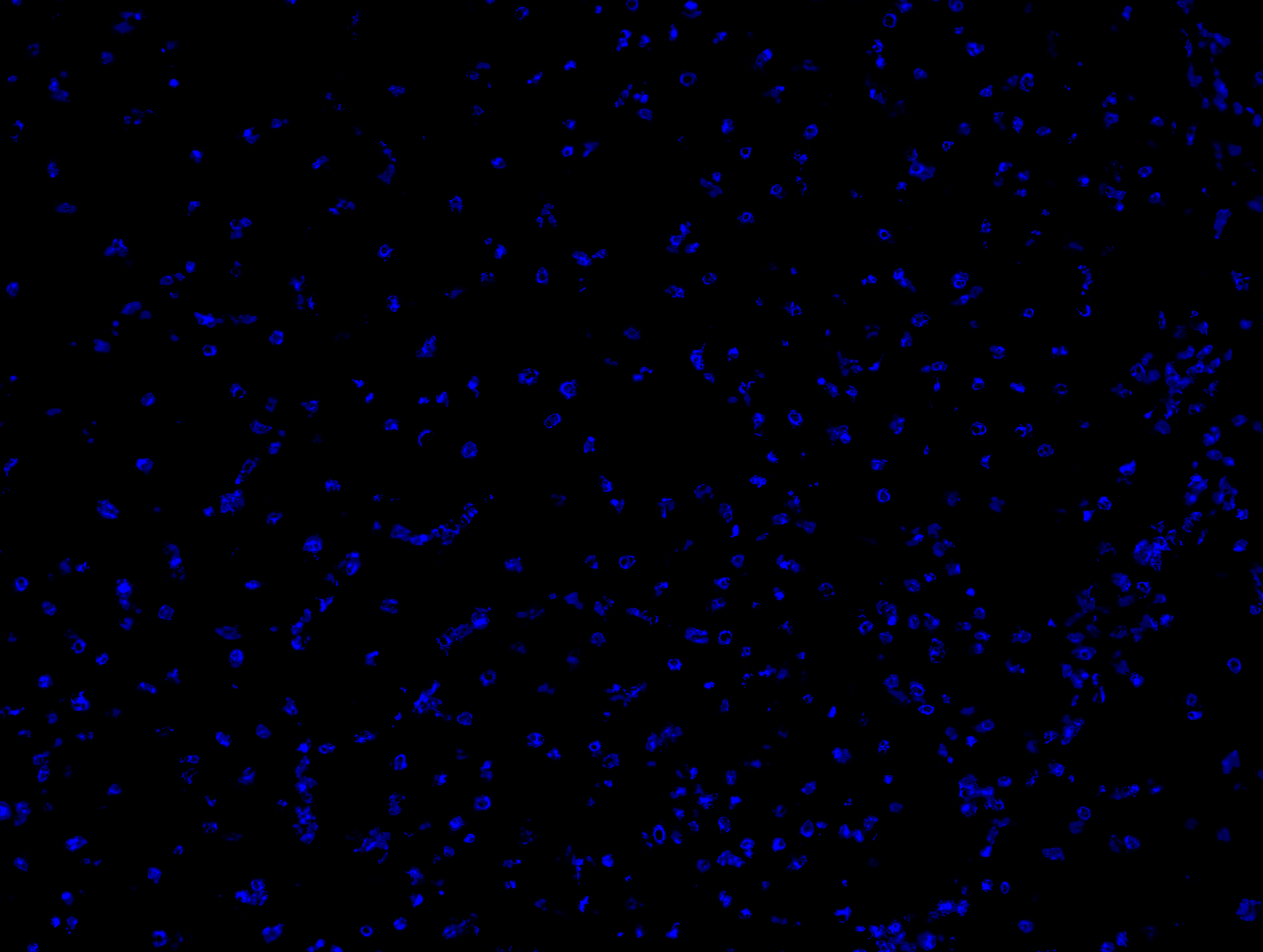

Supplement: Supplementary file 6 — Source Data Fig. 5 [file 44321_2024_49_MOESM6_ESM.zip › Figure 5/5A/PMO/Q DAPI.tif]

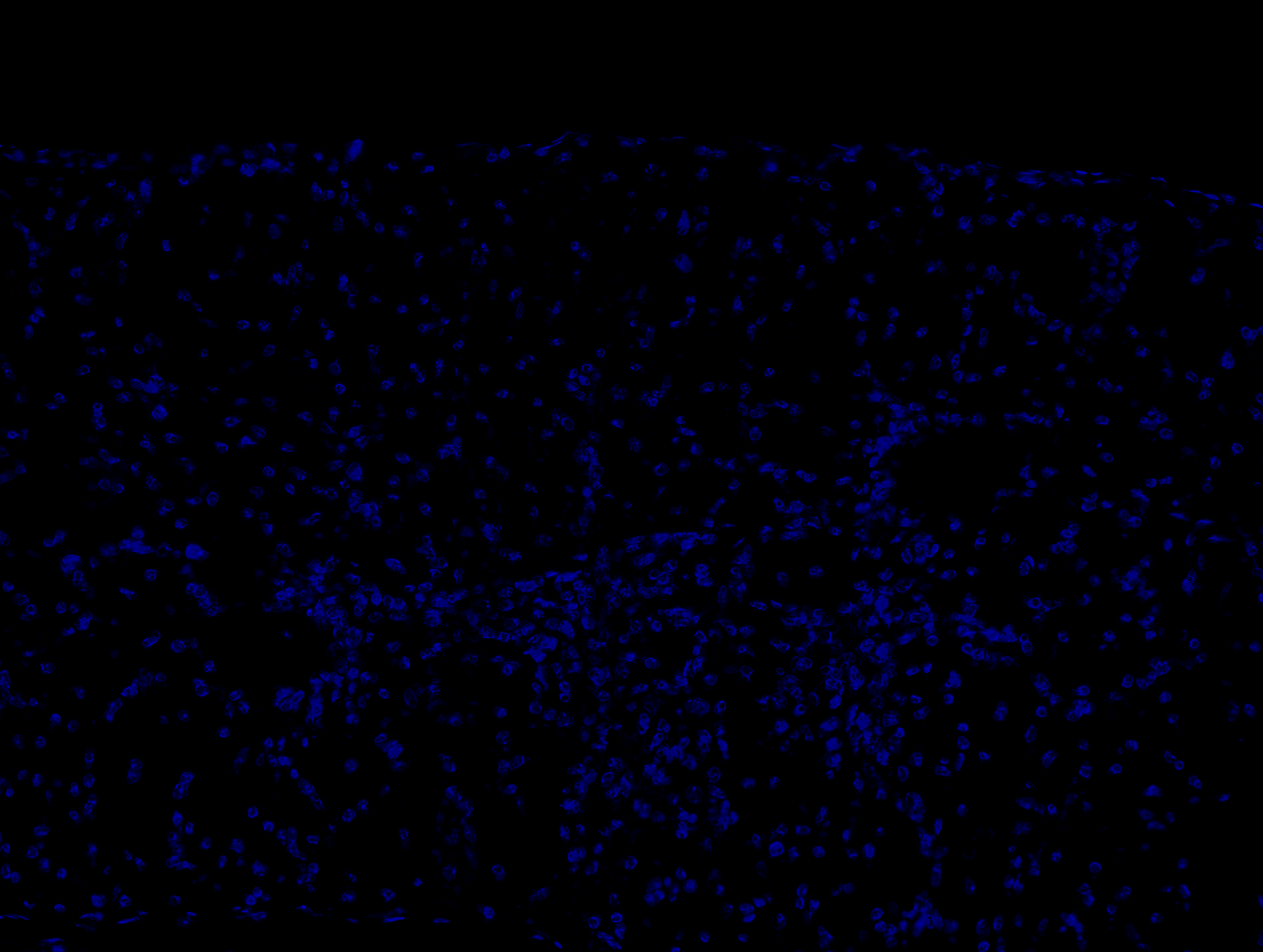

Supplement: Supplementary file 6 — Source Data Fig. 5 [file 44321_2024_49_MOESM6_ESM.zip › Figure 5/5A/PMO/D DAPI.tif]

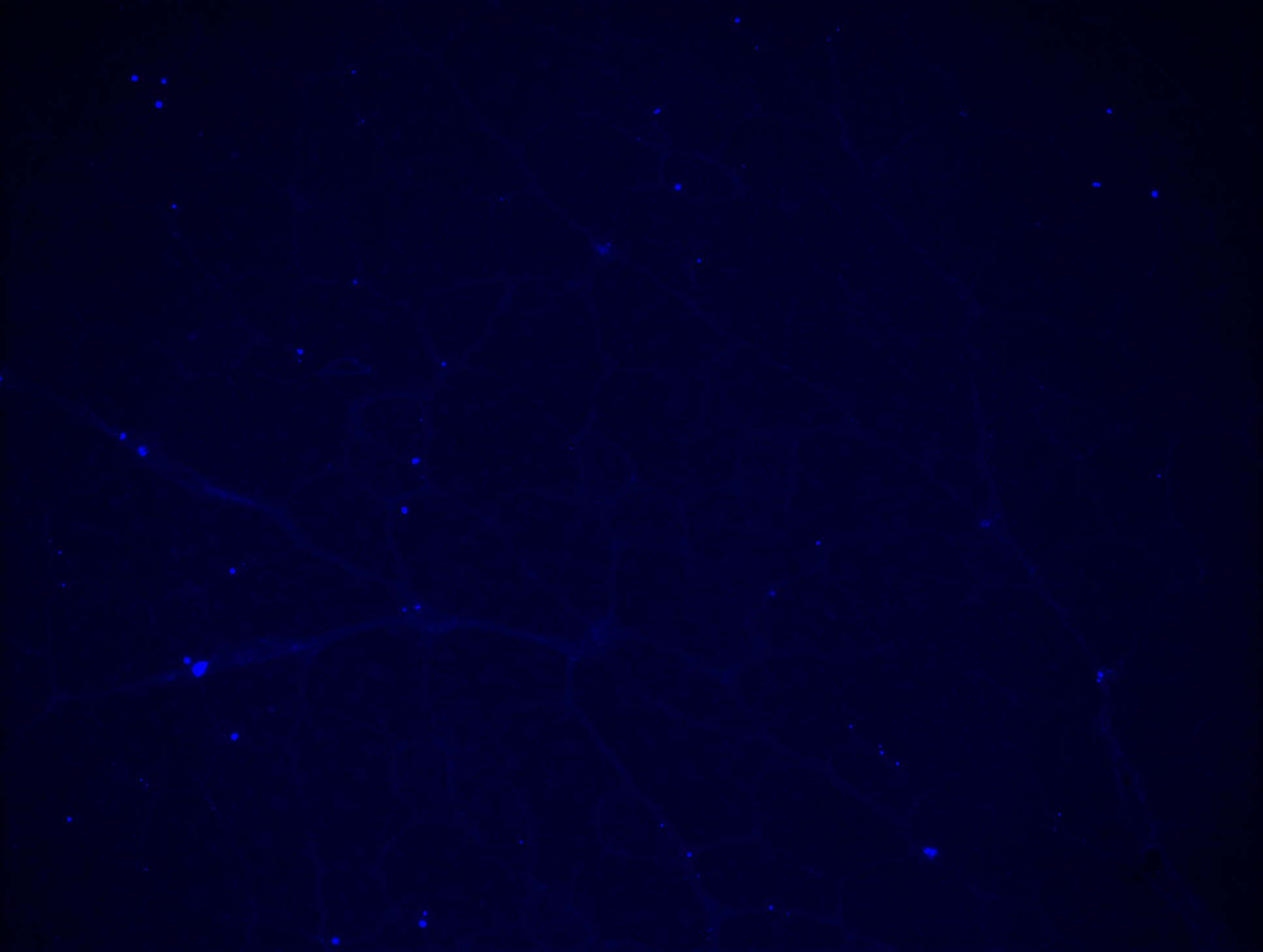

Supplement: Supplementary file 6 — Source Data Fig. 5 [file 44321_2024_49_MOESM6_ESM.zip › Figure 5/5A/C57/T DAPI.tif]

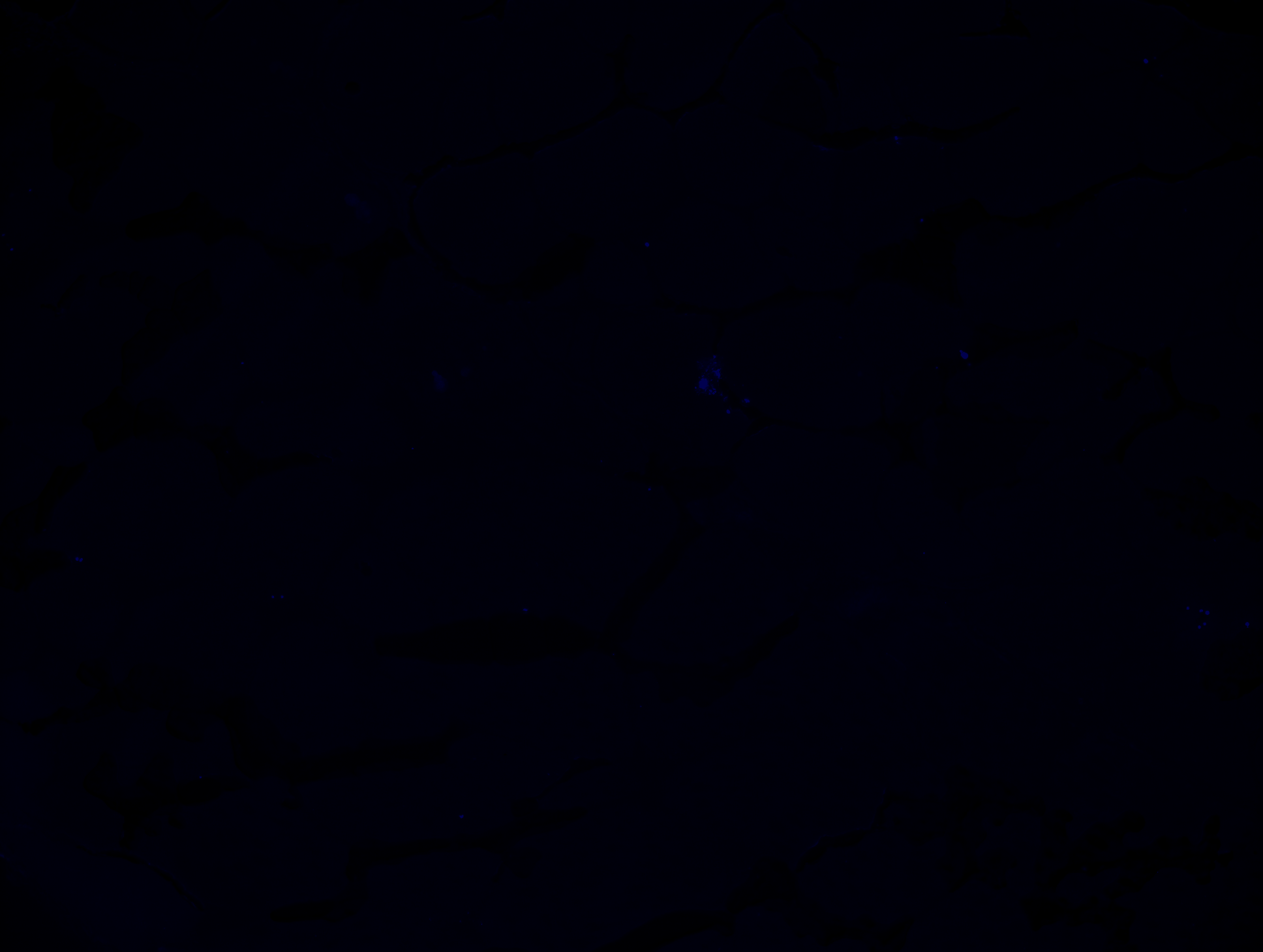

Supplement: Supplementary file 6 — Source Data Fig. 5 [file 44321_2024_49_MOESM6_ESM.zip › Figure 5/5A/C57/A DAPI.tif]

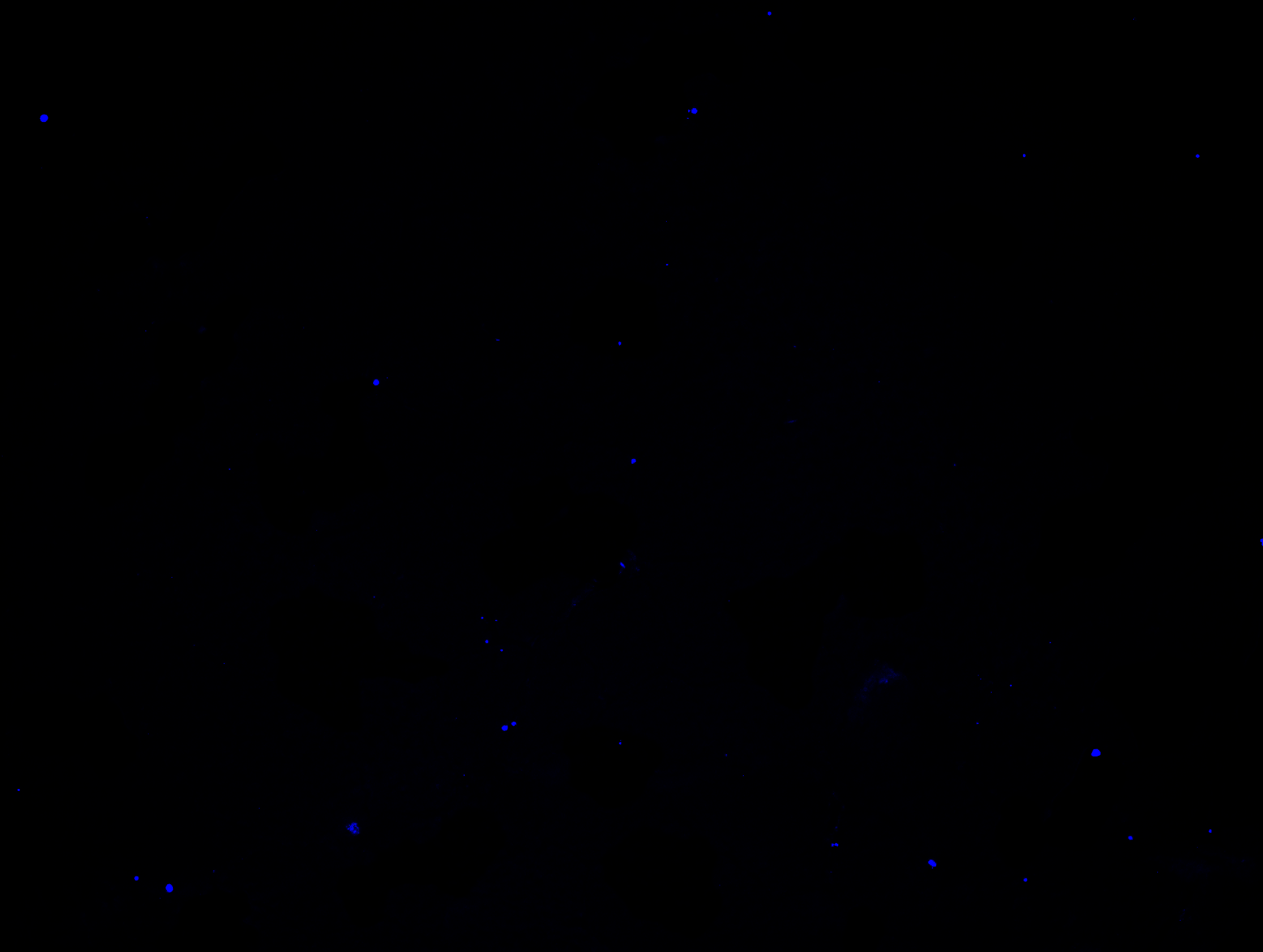

Supplement: Supplementary file 6 — Source Data Fig. 5 [file 44321_2024_49_MOESM6_ESM.zip › Figure 5/5A/C57/G DAPI.tif]

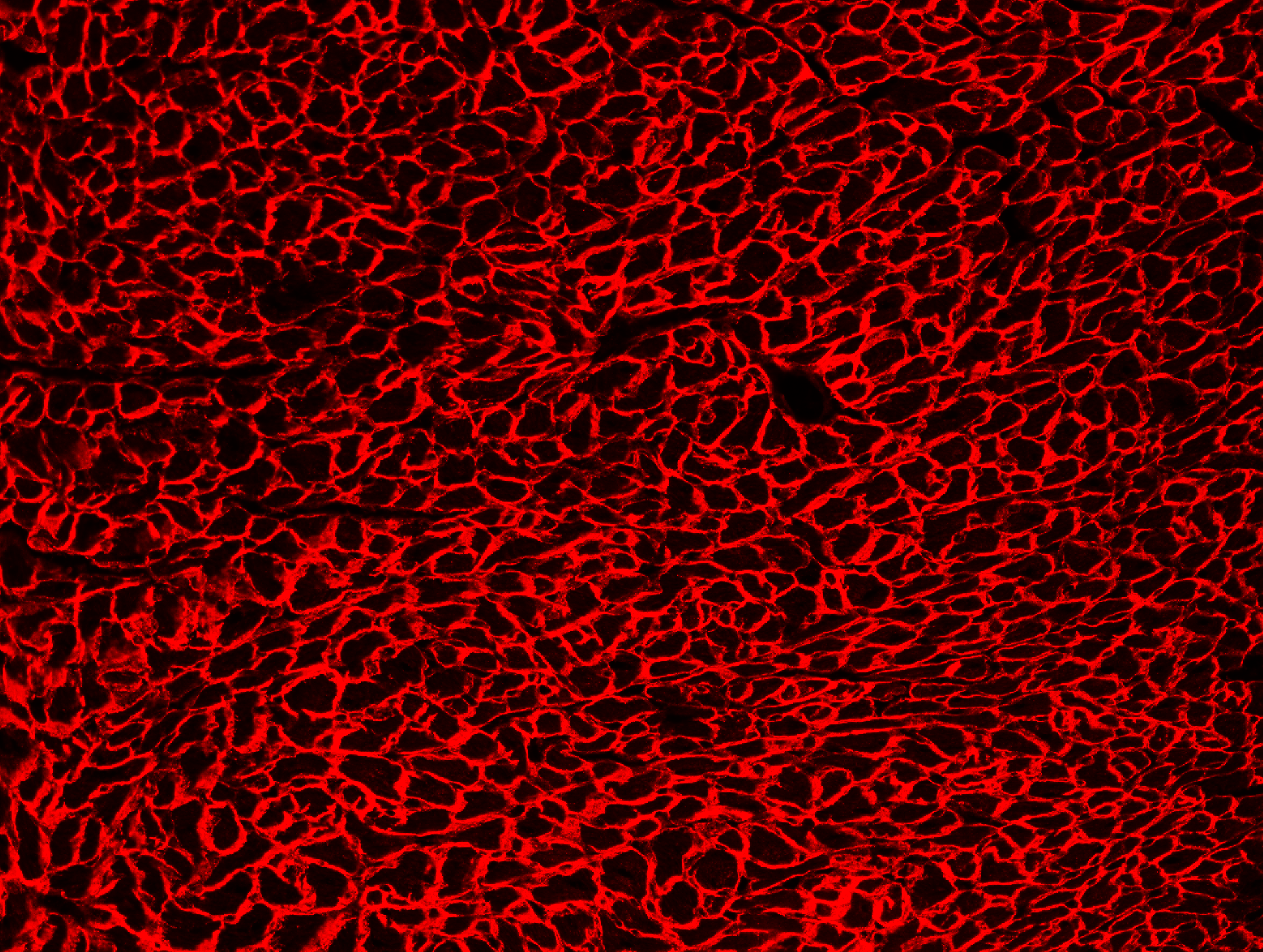

Supplement: Supplementary file 6 — Source Data Fig. 5 [file 44321_2024_49_MOESM6_ESM.zip › Figure 5/5A/C57/H DYS.tif]

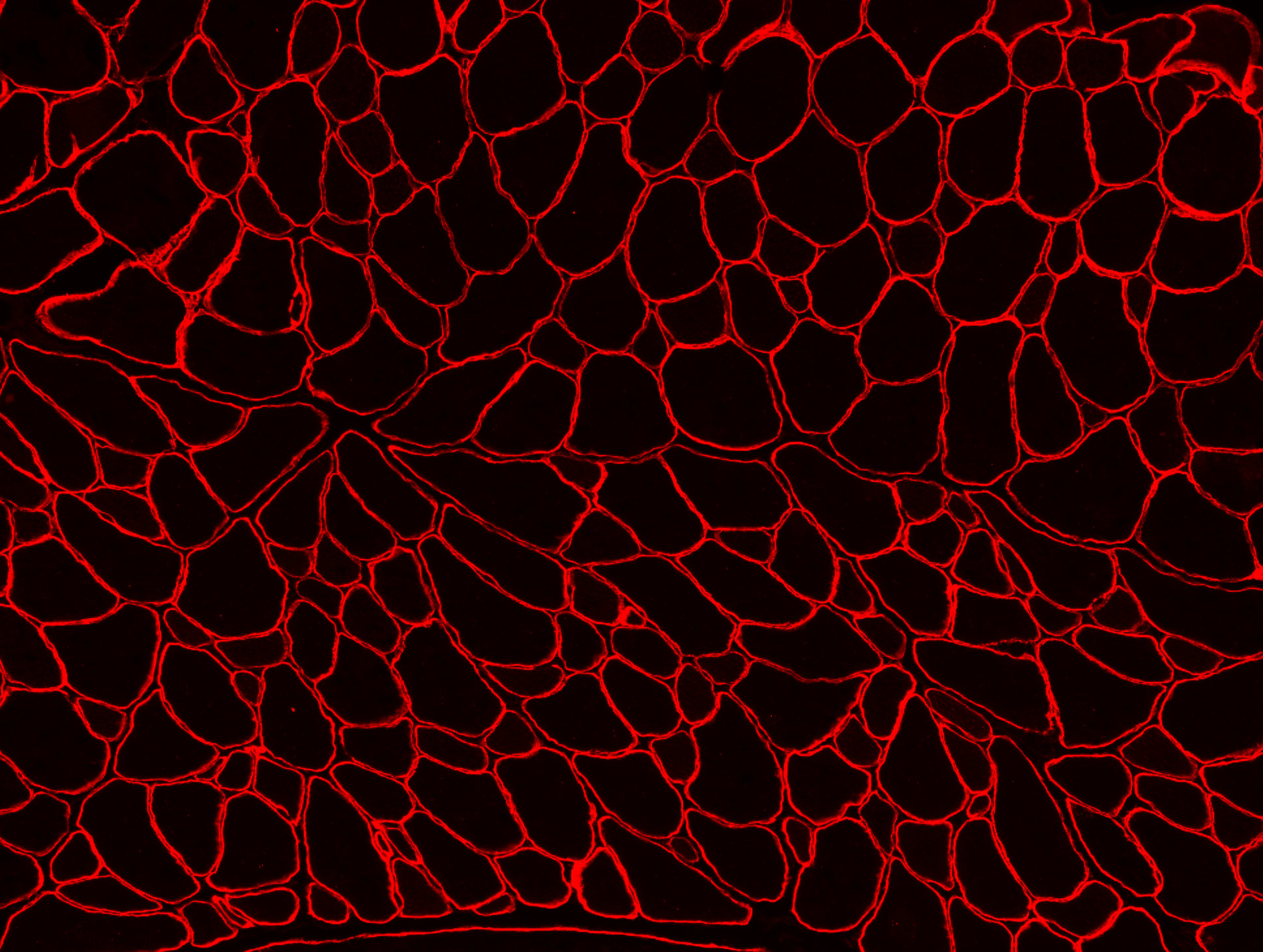

Supplement: Supplementary file 6 — Source Data Fig. 5 [file 44321_2024_49_MOESM6_ESM.zip › Figure 5/5A/C57/A DYS.tif]

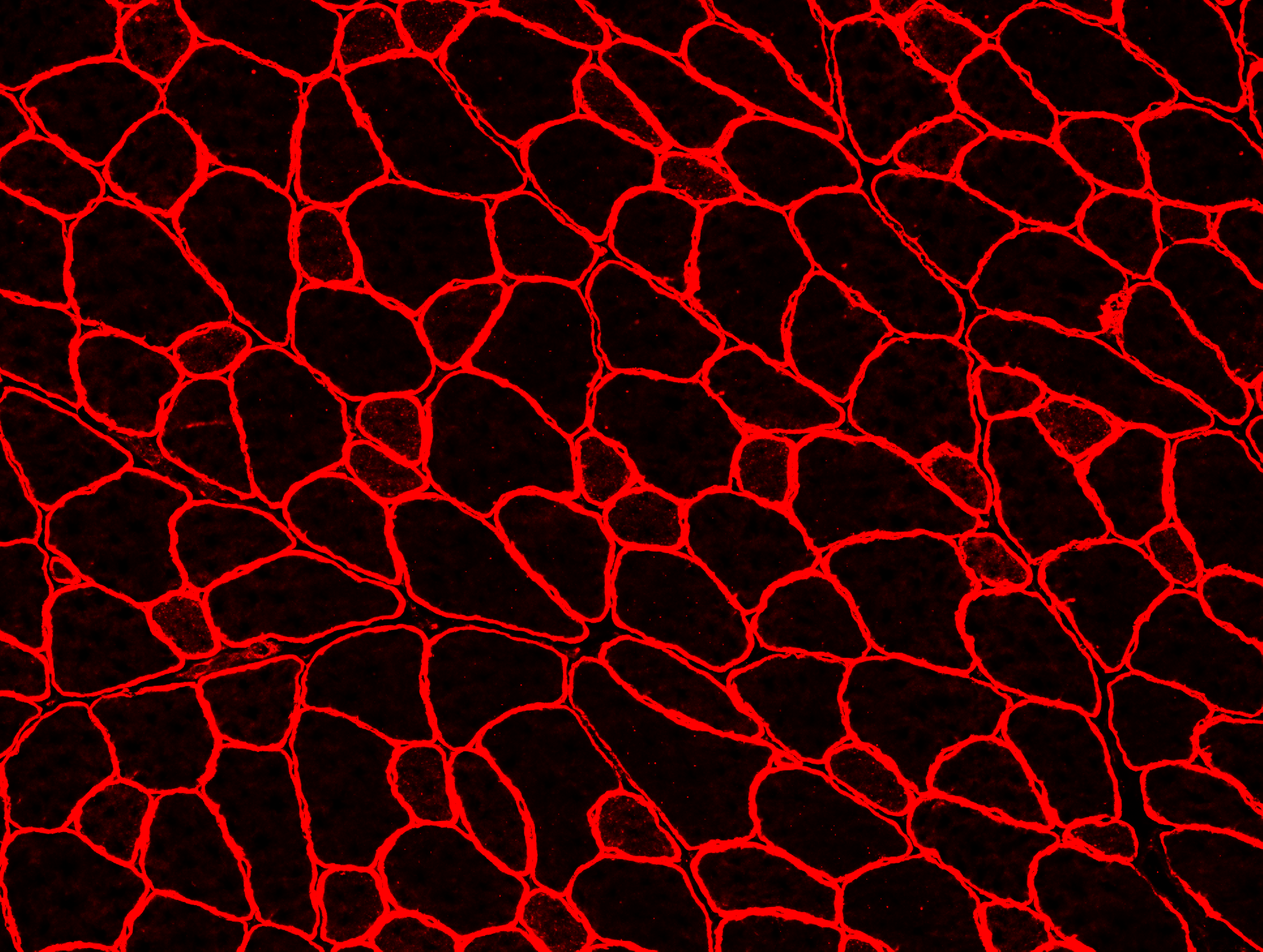

Supplement: Supplementary file 6 — Source Data Fig. 5 [file 44321_2024_49_MOESM6_ESM.zip › Figure 5/5A/C57/T DYS.tif]

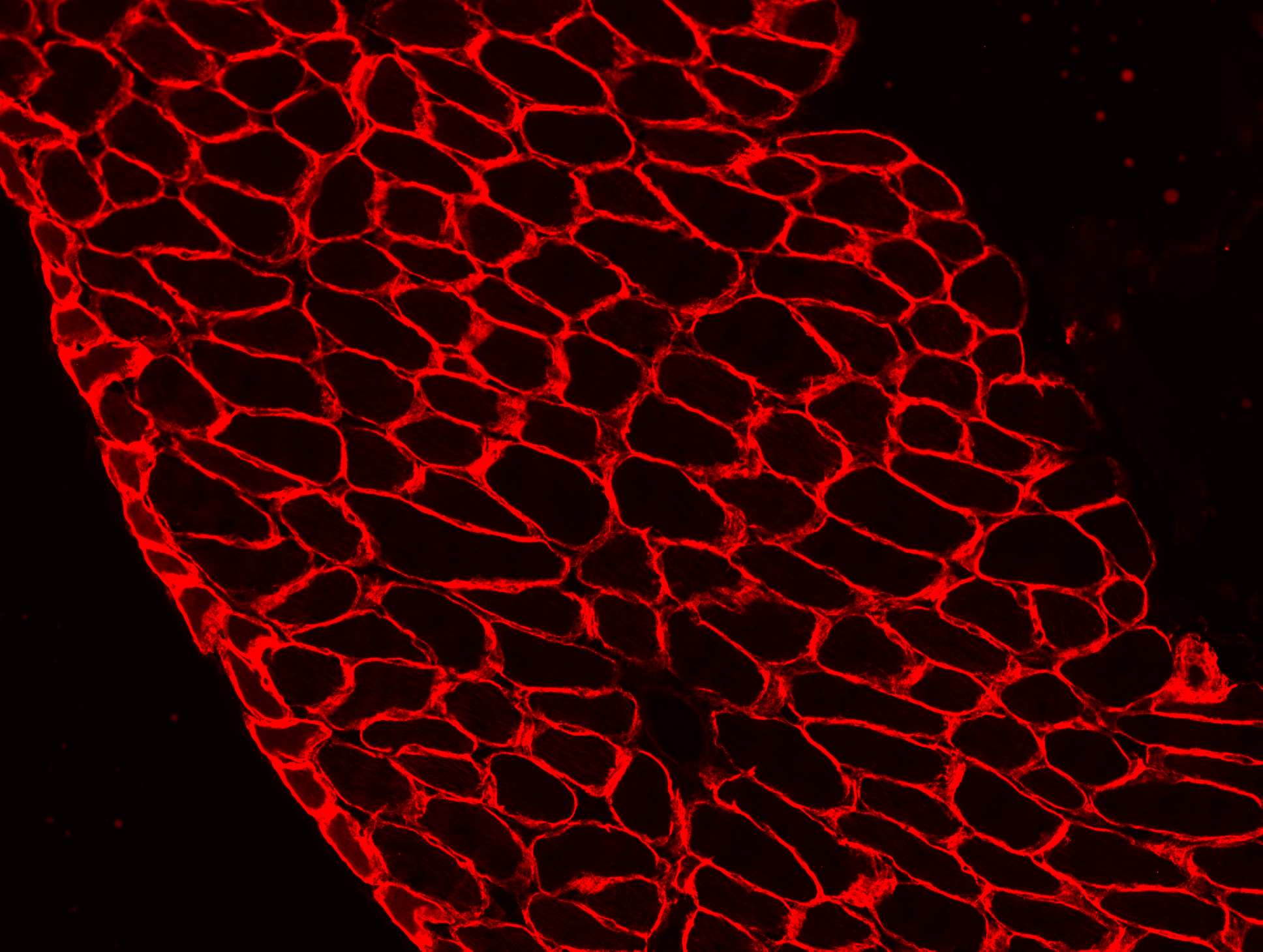

Supplement: Supplementary file 6 — Source Data Fig. 5 [file 44321_2024_49_MOESM6_ESM.zip › Figure 5/5A/C57/D DYS.tif]

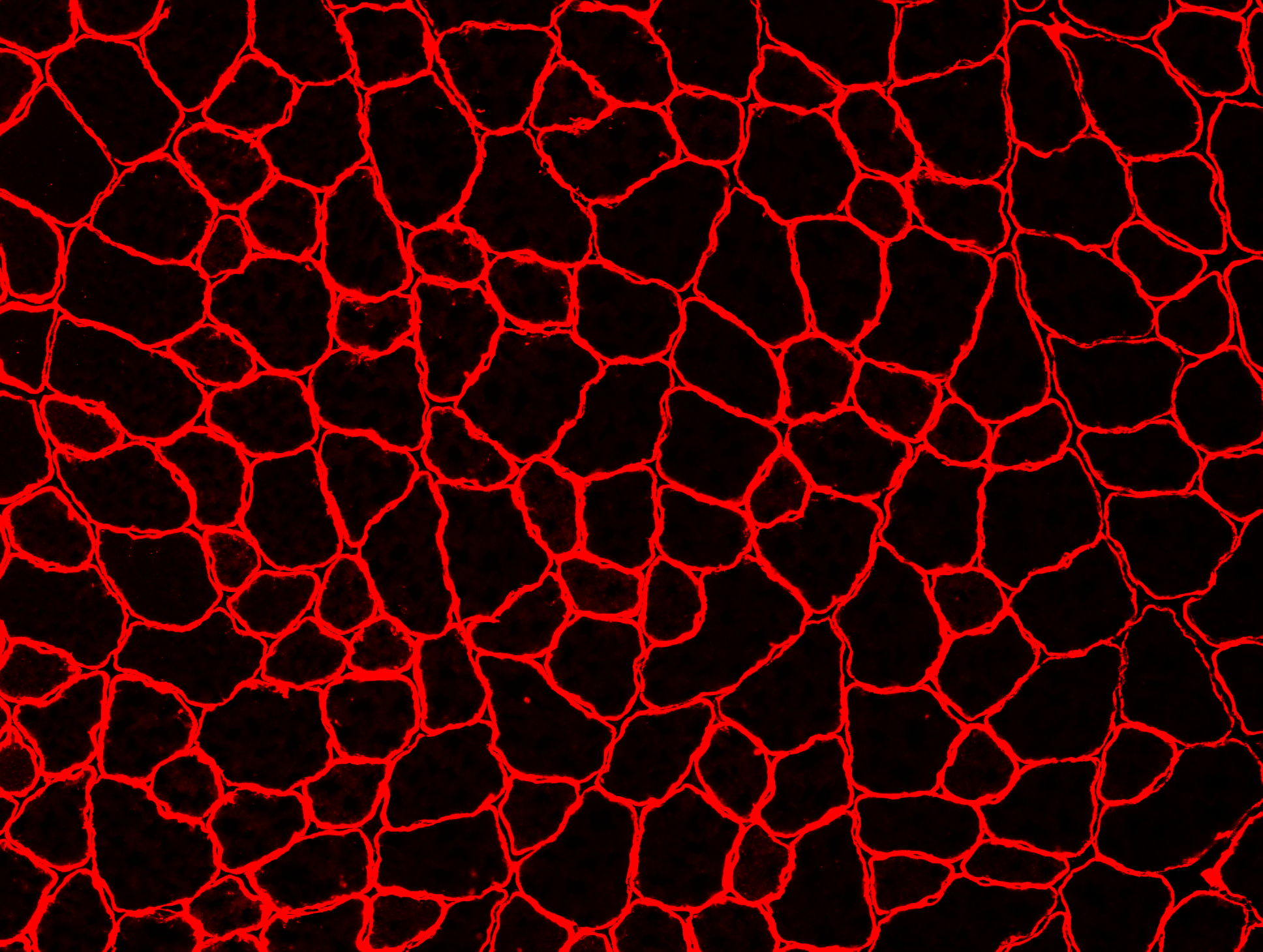

Supplement: Supplementary file 6 — Source Data Fig. 5 [file 44321_2024_49_MOESM6_ESM.zip › Figure 5/5A/C57/Q DYS.tif]

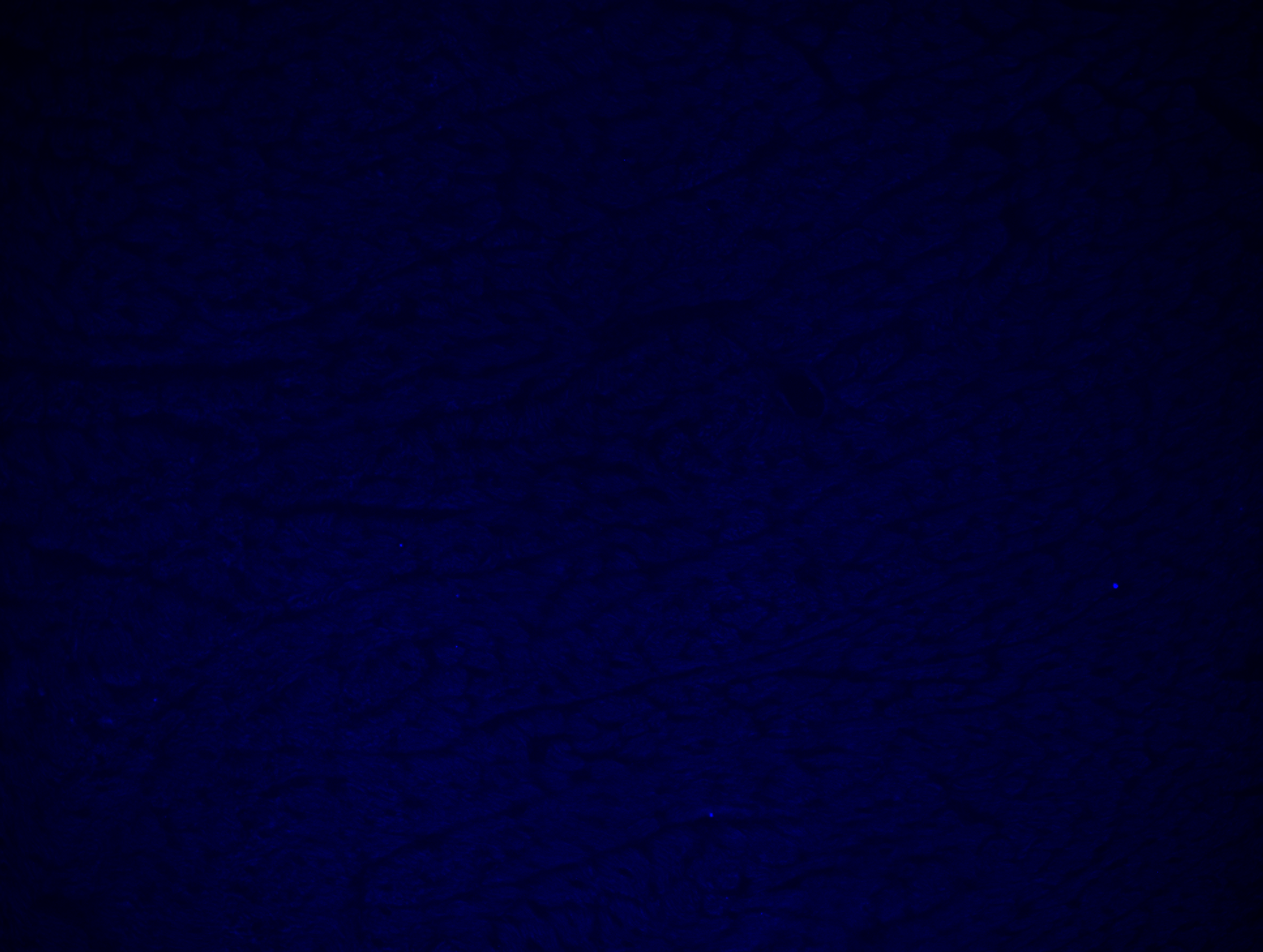

Supplement: Supplementary file 6 — Source Data Fig. 5 [file 44321_2024_49_MOESM6_ESM.zip › Figure 5/5A/C57/H DAPI.tif]

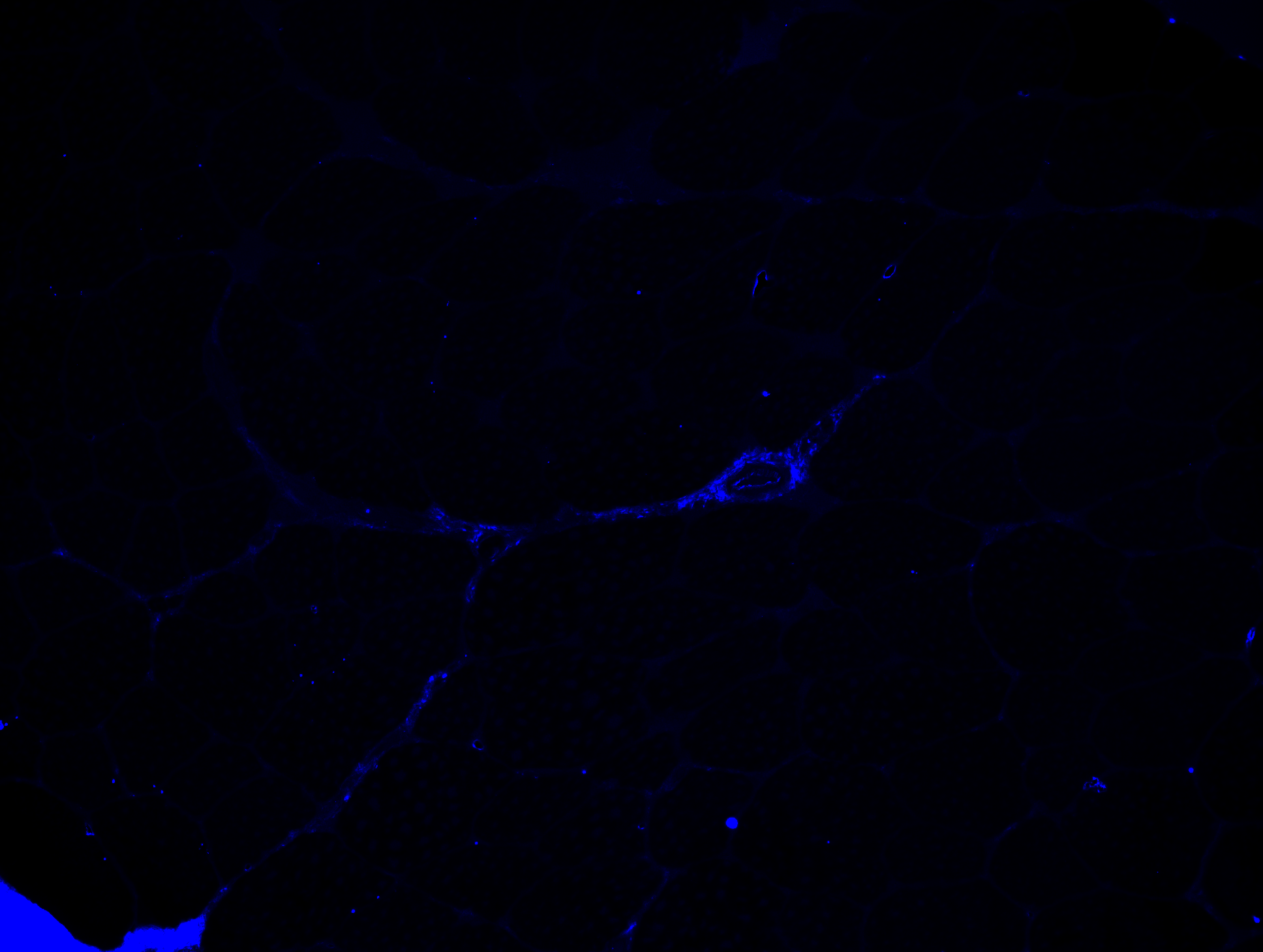

Supplement: Supplementary file 6 — Source Data Fig. 5 [file 44321_2024_49_MOESM6_ESM.zip › Figure 5/5A/C57/TA DAPI.tif]

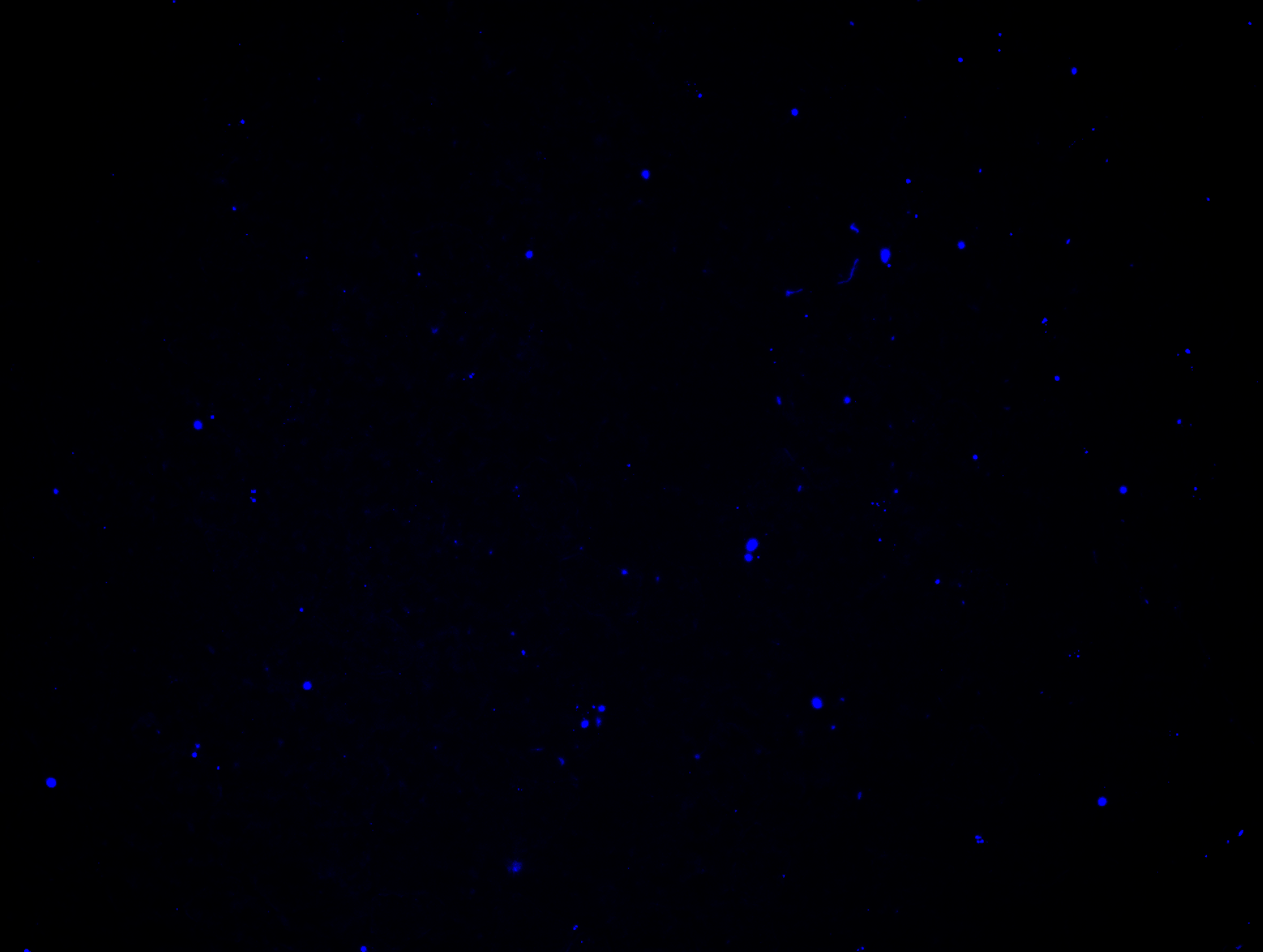

Supplement: Supplementary file 6 — Source Data Fig. 5 [file 44321_2024_49_MOESM6_ESM.zip › Figure 5/5A/C57/Q DAPI.tif]

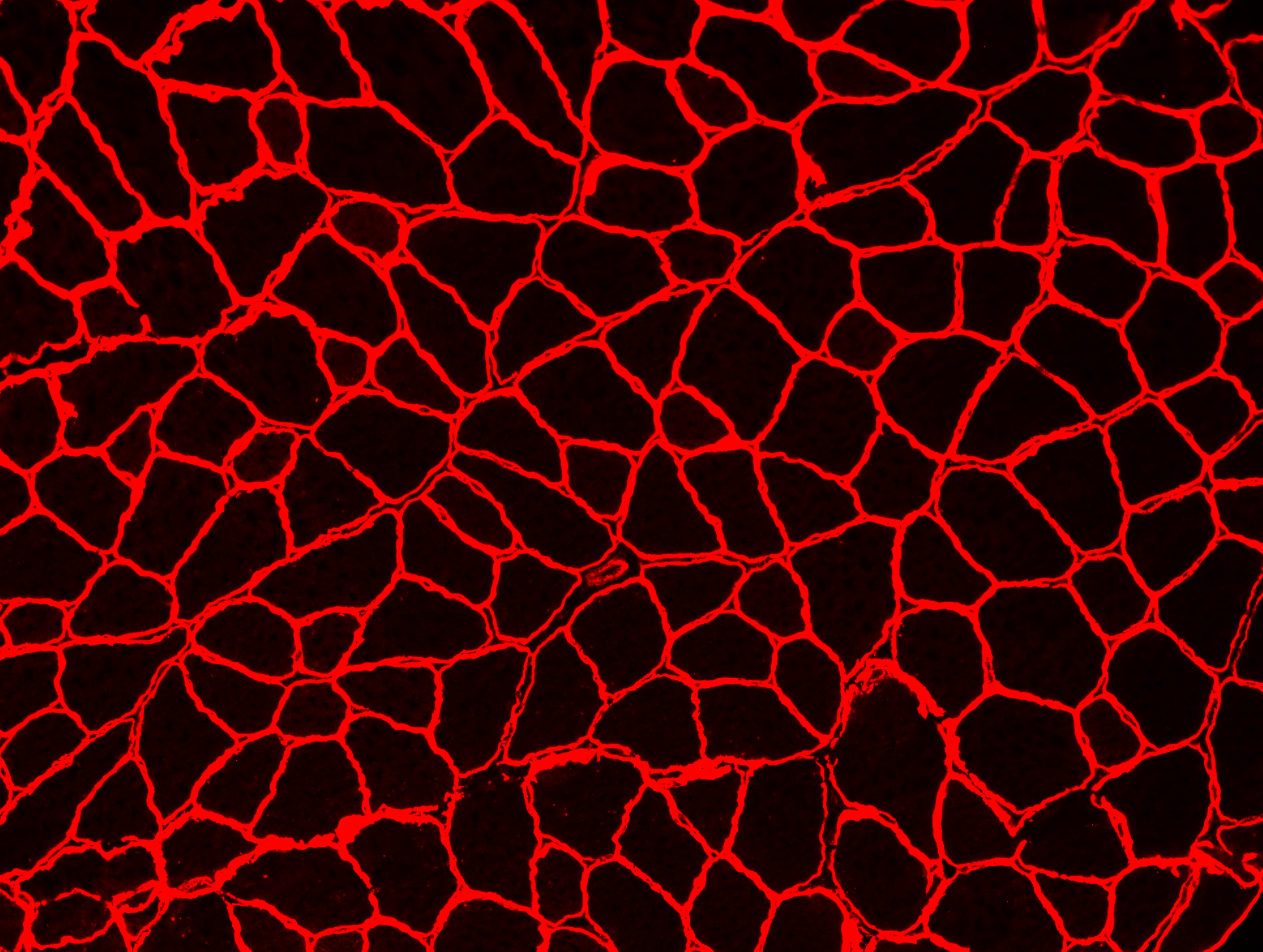

Supplement: Supplementary file 6 — Source Data Fig. 5 [file 44321_2024_49_MOESM6_ESM.zip › Figure 5/5A/C57/G DYS.tif]

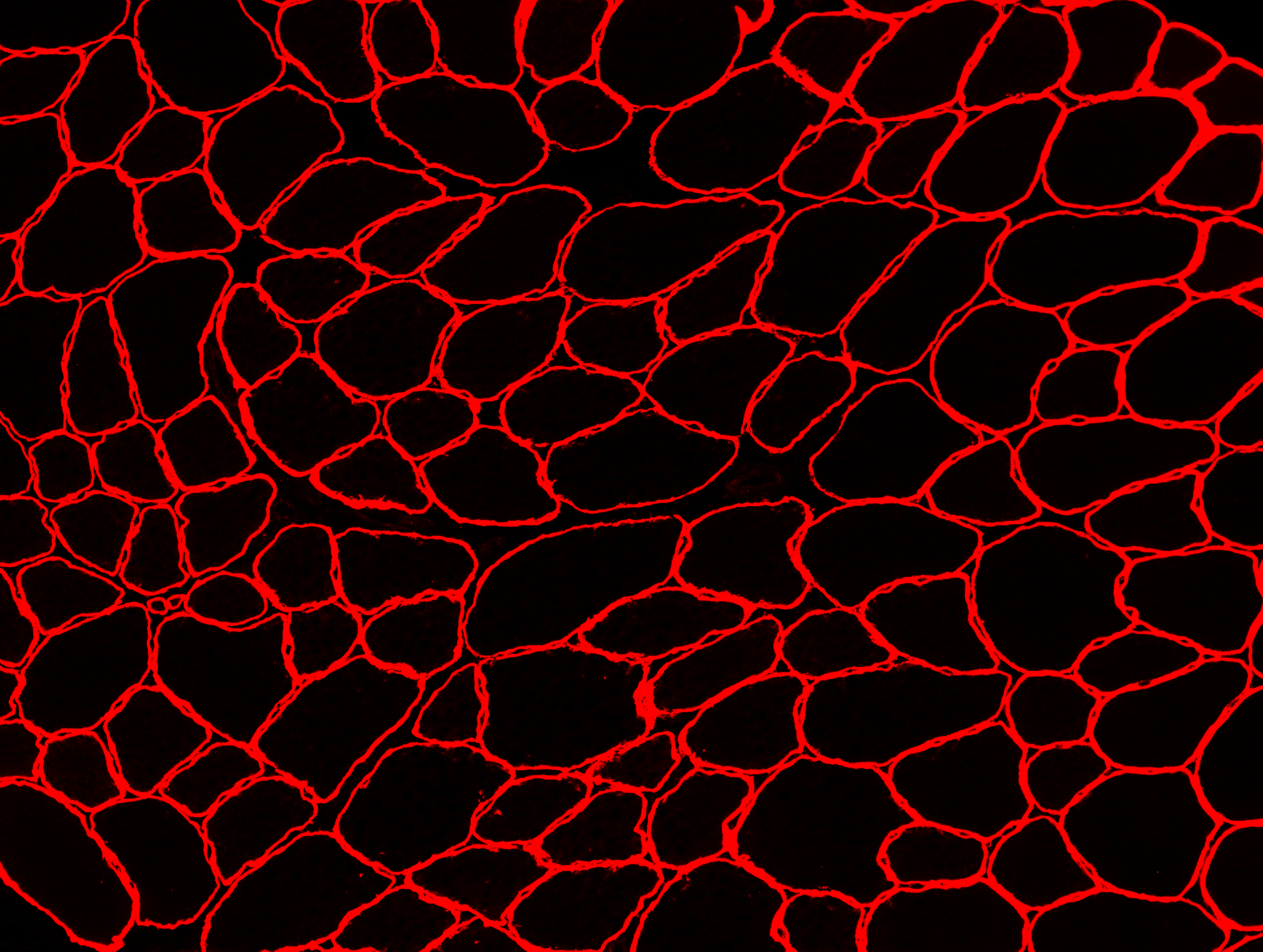

Supplement: Supplementary file 6 — Source Data Fig. 5 [file 44321_2024_49_MOESM6_ESM.zip › Figure 5/5A/C57/TA DYS.tif]

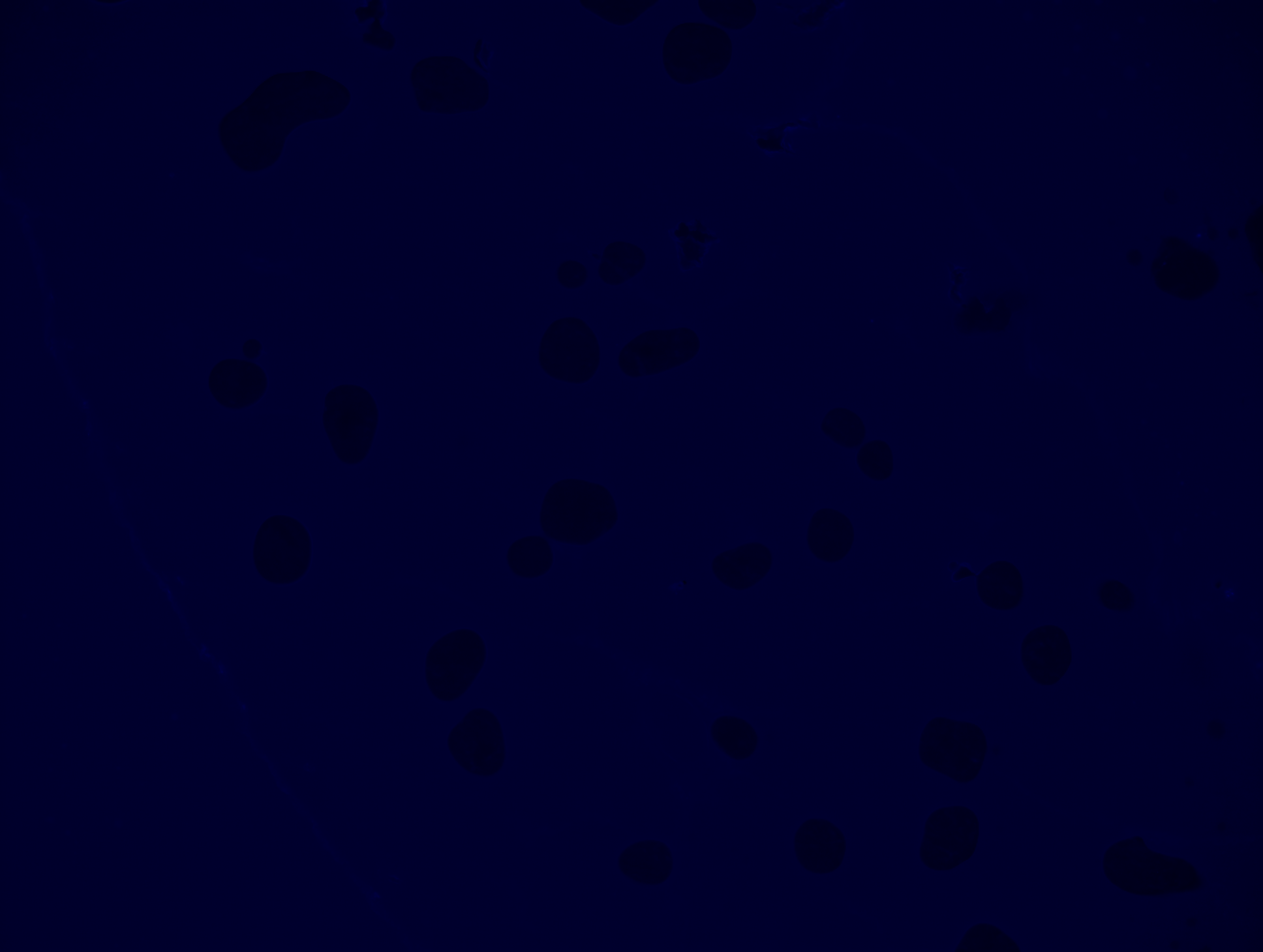

Supplement: Supplementary file 6 — Source Data Fig. 5 [file 44321_2024_49_MOESM6_ESM.zip › Figure 5/5A/C57/D DAPI.tif]
