# Supplementary material for: Generalizable anchor aptamer strategy for loading nucleic acid therapeutics on exosomes
Source: EMBO Mol Med. 2024 Mar 6;16(4):24. doi: 10.1038/s44321-024-00049-7 (PMC11018858; doi:10.1038/s44321-024-00049-7)
Supplement: Supplementary file 8 — Expanded View Figures [file 44321_2024_49_MOESM8_ESM.pdf]

Expanded View Figures

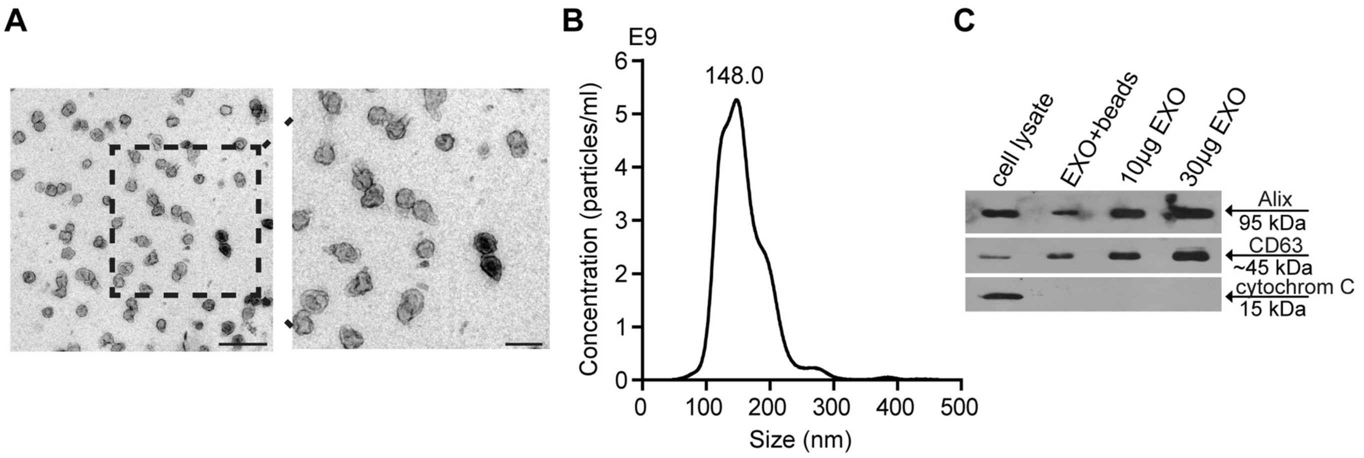

Figure EV1. Characterization of murine myotube-derived exosomes and CP05-based binding assay.

(**A**) Representative transmission electron microscopic (TEM) images of exosomes derived from murine myotubes (scale bar = 200 nm for right panel and 500 nm for the left). (**B**) Size distribution of exosomes derived from murine myotubes with nanoparticle tracking analysis (NTA). (**C**) Western blot to examine the binding of murine myotube-derived exosomes (EXO) to CP05-coated beads. The loading was specified in the figure.

© The Author(s)

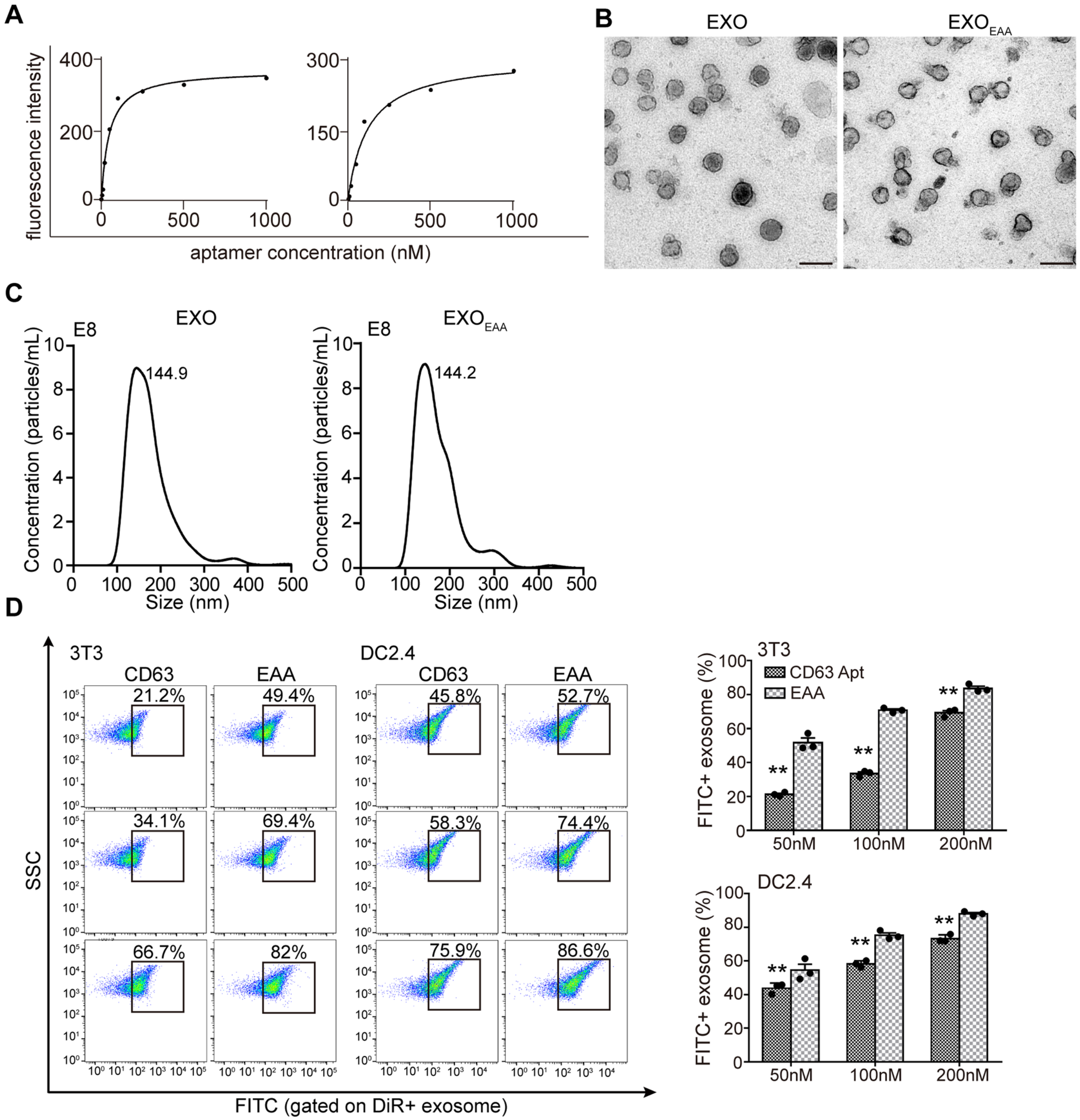

Figure EV2. Characterization of murine myotube-derived exosomes modified with EAA and binding assay for EAA with different exosomes.

(**A**) Measurement of binding affinity of EAA or CD63 aptamer to exosomes derived from mouse serum with flow cytometry. (**B**) Representative TEM images of murine myotube-derived exosomes modified with EAA (EXO_{EAA}) (scale bar = 200 nm). (**C**) Size distribution of EXO and EXO_{EAA} with nanoparticle tracking analysis (NTA). (**D**) Flow cytometric and quantitative analysis of binding efficiency of EAA or CD63 aptamer to exosomes across different concentrations ($n = 3$). Exosomes were derived from murine 3T3 and DC2.4 cells. Data information: Data represent different numbers (n) of biological replicates. The data with error bars are shown as mean ± SEM. (**D**) Statistical significance was determined using two-tailed t test. $^{**}P < 0.001$.

 © The Author(s)

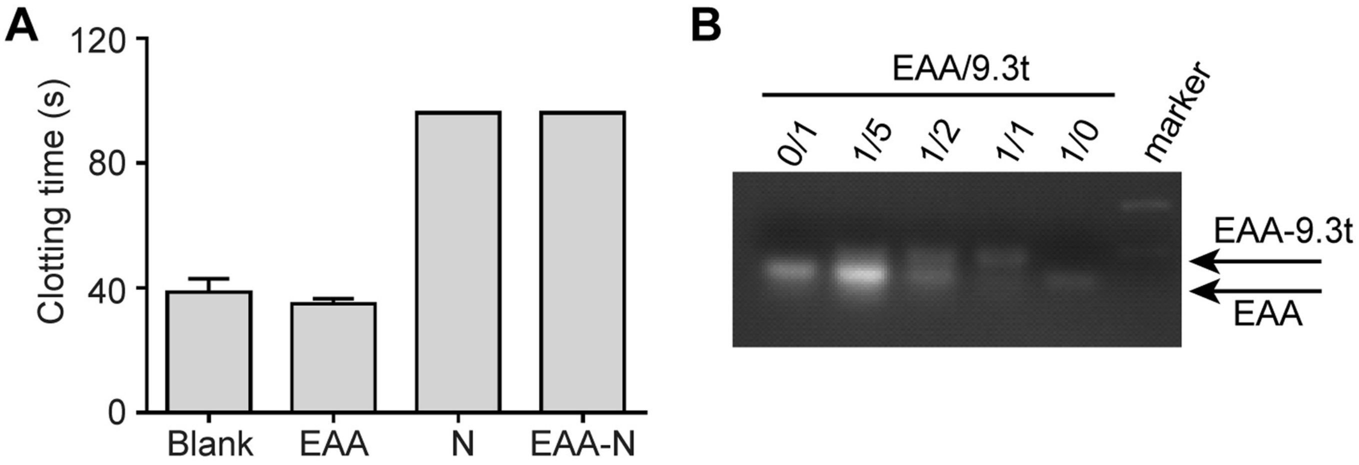

Figure EV3. In vitro evaluation of EAA-NU172's activity and optimization of binding ratio of EAA to 9.3t RNA aptamers.

(**A**) Measurement of the clotting time of rat plasma after the addition of thrombin in the presence of EAA, NU172 (N) or EAA-NU172 (EAA-N) ($n = 3$). Data represent different numbers (n) of biological replicates. The data with error bars are shown as mean ± SEM. (**B**) Agarose gel for mobility shift assay to determine the binding of EAA-linker to linker-9.3t at different molar ratios.

© The Author(s)

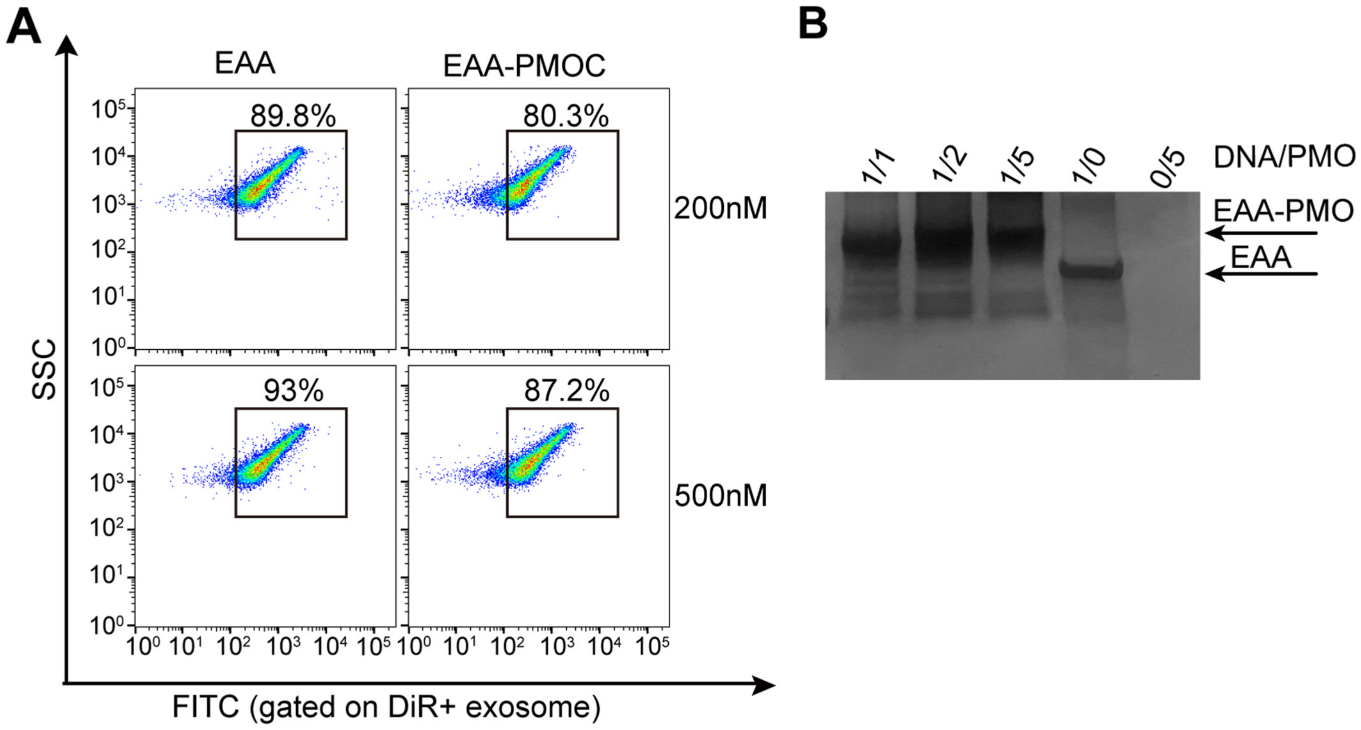

Figure EV4. Evaluation of binding efficiency of EAA-PMOC to exosomes and optimization of binding of EAA-PMOC to PMO.

(**A**) Flow cytometric analysis of binding efficiency of EAA-PMOC to exosomes derived from murine myotubes at two different concentrations. EAA-PMOC refers to EAA fused with a PMO- Complementary sequence. Aptamers were labeled with FITC and exosomes were labeled with DiR. (**B**) Silver staining for mobility shift assay to determine the binding of EAA-PMOC to PMO at different molar ratios.

© The Author(s)

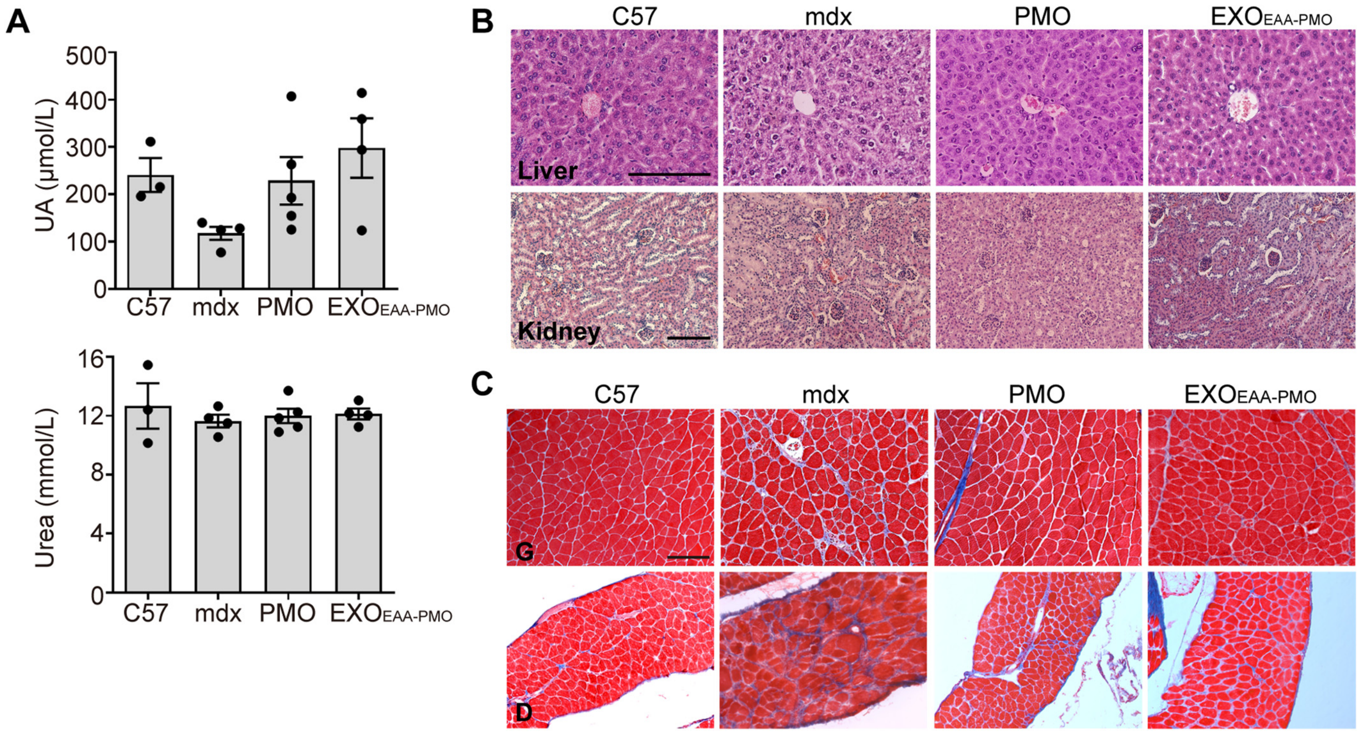

Figure EV5. Biochemical and histological examination of *mdx* mice treated with $EXO_{EAA\text{-}PMO}$.

$EXO_{EAA\text{-}PMO}$ at the PMO dose of 25 mg/kg were administered into adult *mdx* mice for three times weekly intravenously and tissues were harvested 2 weeks after last injection. (**A**) Analysis of biochemical indicators for kidney function in mdx mice treated with PMO (n = 5), $EXO_{EAA\text{-}PMO}$ (*n* = 4), untreated *mdx* (*n* = 4) and *C57BL/6* controls (*n* = 3). (**B**) H&E staining of liver and kidney from *mdx* mice treated with $EXO_{EAA\text{-}PMO}$ (scale bar = 100 μm). (**C**) Collagen deposition analysis in gastrocnemius (G) and quadriceps (Q) from treated *mdx* mice (scale bar = 100 μm). Data information: In (**A**), the data represent different numbers (*n*) of biological replicates. The data with error bars are shown as mean ± SEM.

© The Author(s)
